# Selective ion transport through hydrated micropores in polymer membranes

**DOI:** 10.1038/s41586-024-08140-2

**Published:** 2024-11-06

**Authors:** Anqi Wang, Charlotte Breakwell, Fabrizia Foglia, Rui Tan, Louie Lovell, Xiaochu Wei, Toby Wong, Naiqi Meng, Haodong Li, Andrew Seel, Mona Sarter, Keenan Smith, Alberto Alvarez‐Fernandez, Mate Furedi, Stefan Guldin, Melanie M. Britton, Neil B. McKeown, Kim E. Jelfs, Qilei Song

**Affiliations:** 1 Department of Chemical Engineering, Imperial College London, London, UK; 2 Department of Chemistry, Molecular Sciences Research Hub, Imperial College London, London, UK; 3 Department of Chemistry, University College London, London, UK; 4 School of Chemistry, University of Birmingham, Birmingham, UK; 5 ISIS Neutron and Muon Source, Science & Technology Facilities Council, Rutherford Appleton Laboratory, Harwell Science and Innovation Campus, Chilton, UK; 6 Department of Physics, Royal Holloway University of London, Egham, UK; 7 Department of Chemical Engineering, University College London, London, UK; 8 EaStCHEM, School of Chemistry, University of Edinburgh, Edinburgh, UK; 9 Present Address: Physical Science and Engineering Division, King Abdullah University of Science and Technology, Thuwal, Saudi Arabia

**Keywords:** Batteries, Porous materials, Soft materials, Polymer chemistry, Chemical engineering

## Abstract

Ion-conducting polymer membranes are essential in many separation processes and electrochemical devices, including electrodialysis^1^, redox flow batteries^2^, fuel cells^3^ and electrolysers^4,5^. Controlling ion transport and selectivity in these membranes largely hinges on the manipulation of pore size. Although membrane pore structures can be designed in the dry state^6^, they are redefined upon hydration owing to swelling in electrolyte solutions. Strategies to control pore hydration and a deeper understanding of pore structure evolution are vital for accurate pore size tuning. Here we report polymer membranes containing pendant groups of varying hydrophobicity, strategically positioned near charged groups to regulate their hydration capacity and pore swelling. Modulation of the hydrated micropore size (less than two nanometres) enables direct control over water and ion transport across broad length scales, as quantified by spectroscopic and computational methods. Ion selectivity improves in hydration-restrained pores created by more hydrophobic pendant groups. These highly interconnected ion transport channels, with tuned pore gate sizes, show higher ionic conductivity and orders-of-magnitude lower permeation rates of redox-active species compared with conventional membranes, enabling stable cycling of energy-dense aqueous organic redox flow batteries. This pore size tailoring approach provides a promising avenue to membranes with precisely controlled ionic and molecular transport functions.

## Abstract (web summary)

A method for design of polymer membranes uses strategically placed pendant groups with specific hydrophobicity to precisely tailor hydrated pore size, with applications in ion-conducting membranes for redox flow batteries.

## Main

Commercial ion-exchange membranes such as Nafion^7^ and sulfonated poly(ether ether ketone) (*s*PEEK)^8^ have complex microphase-separated structures, in which ionic domains form nanometre-sized water channels upon hydration. The ill-defined structure of these channels results in a ubiquitous trade-off between conductivity and selectivity for ion transport. Microporous materials with intrinsic cavities, particularly solution-processable polymers of intrinsic microporosity (PIMs)^9–11^ and polyphenylenes^12,13^, show potential as high-performance membranes but still have performance limitations^14^. Although charge-neutral PIMs retain suitable pore dimensions for selective ion transport, they often exhibit moderate to low ionic conductivity^15,16^. Efforts to enhance conductivity in PIMs by introducing charged moieties typically cause excessive pore swelling, which degrades ion selectivity^14,17^. Overcoming the conductivity–selectivity trade-off requires precise structural control of membrane hydrated pores.

The key challenge is how to modulate pore hydration to generate appropriate pore sizes in hydrated membranes. Confining sulfonate groups within a rigid, amorphous framework significantly reduces swelling and enhances ion–pore interactions, enabling near-frictionless ion transport^18,19^. Crystalline materials, such as metal^20,21^ and covalent^22,23^ organic frameworks and porous organic cages^24^, offer superior ion selectivity owing to their confined, ordered pores. Nevertheless, translating this nanoconfinement approach to linear polymers, which are more prone to swelling but offer advantageous solution processability, remains challenging.

**Fig. 1:**
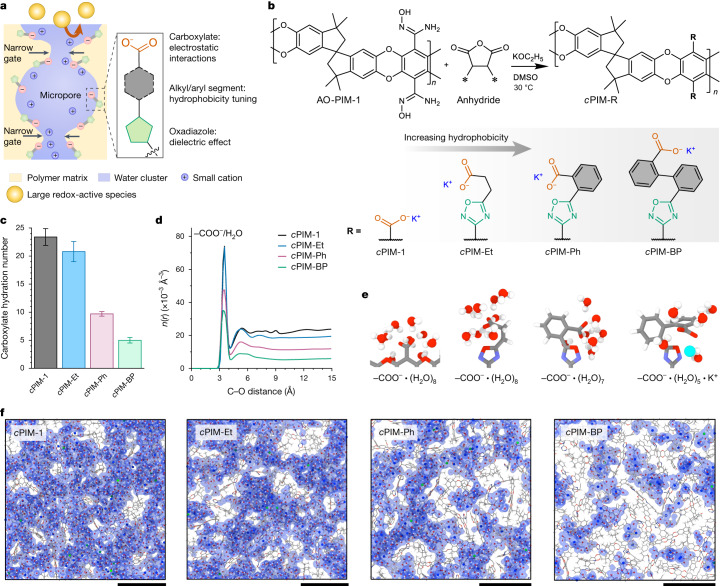
Design of polymer membranes with regulated pore hydration. **a**, Schematic showing the architecture of hydrated micropores. **b**, Synthetic route and chemical structures of *c*PIMs containing pendant groups with varying hydrophobicity. DMSO, dimethyl sulfoxide. **c**, Hydration numbers of carboxylate groups measured in 1 M aqueous KCl at 25 °C. **d**, Modelled radial number density distribution function, *n*(*r*), of water molecules with respect to carboxylate groups in each polymer. Error bars represent standard deviations from five independent simulated polymer boxes. **e**, Images of polymer pendant groups and the first hydration shells. These images were generated from the simulated boxes of hydrated polymers. **f**, Cross-sections (10 Å in thickness) of the simulated boxes of hydrated polymers. Polymer chains are shown in line-and-stick representation where grey, red, navy and white represent carbon, oxygen, nitrogen and hydrogen, respectively. Water and ions are shown in ball-and-stick representation where red, white, blue and green represent oxygen, hydrogen, potassium and chloride, respectively. Light blue shading highlights the isosurface of water to visualize water clusters. Box lengths are 67 Å, 68 Å, 62 Å and 62 Å for *c*PIM-1, *c*PIM-Et, *c*PIM-Ph and *c*PIM-BP, respectively. Scale bars, 2 nm.

Certain proteins and synthetic supramolecular hosts possess nonpolar cavities that stay dry in aqueous environments, owing to the thermodynamic penalty imposed by their hydrophobic structural segments^25,26^. Inspired by these systems, we propose tailoring the local hydrophobic environment around ion-conducting moieties as a thermodynamic strategy for tightly regulating pore hydration in solution-processable linear polymers. Our materials design involves (1) a network of interconnected micropores enforced by a rigid, contorted macromolecular structure; (2) controlling local hydrophobicity by varying the number of aromatic rings attached to charged groups; and (3) creating a targeted pore environment for cooperative polymer–ion interactions to ensure fast ion conduction (Fig. 1a). This approach enables effective tuning of pore gate sizes within the subnanometre range and control of the interconnectivity of confined water clusters. Properly regulated hydrophobicity creates narrow gates connecting micropores, while ensuring full percolation of aqueous solutions within the pore network. However, further enhanced hydrophobicity leads to isolated water clusters and excessively small pores, generating significant energy barriers that impede efficient ion transport. Properly sized pore gates are essential for maintaining the size-sieving capability of polymer membranes in aqueous electrochemical devices during long-term operation.

## Tuning pore size in hydrated membranes

We chose amidoxime-functionalized PIM (AO-PIM-1) as a precursor and exploited the high reactivity of amidoximes with anhydrides to introduce carboxylate groups, which formed negatively charged sites within membrane micropores (Fig. 1b and Supplementary Figs. 1–4). We synthesized three polymer variants by introducing ethyl-, phenyl- and biphenyl-containing pendant groups between polymer backbones and carboxylate functionalities, denoted by *c*PIM-Et, *c*PIM-Ph and *c*PIM-BP, respectively. Despite reduced nitrogen sorption capacities at 77 K, these *c*PIMs maintained moderate carbon dioxide uptake at 273 K, suggesting the presence of micropores after pendant group introduction (Supplementary Fig. 5). A carboxylated PIM-1 (*c*PIM-1) without any pendant linking groups was also synthesized by acid hydrolysis of PIM-1.

We postulated that increasing the number of aromatic rings in pendant groups would enhance local pore hydrophobicity, thereby providing opportunities to control the hydration of hygroscopic carboxylate groups. Measurements of water vapour and electrolyte solution uptake showed significant variations in carboxylate hydration number (*λ*) across the *c*PIM series (Supplementary Figs. 6–9). For example, for uptake of 1 M KCl solution, *λ* ranged from 23.4 ± 1.5 for *c*PIM-1 to only 5.0 ± 0.5 for *c*PIM-BP (Fig. 1c and Supplementary Table 1). Radial distribution functions (RDFs) derived from molecular dynamics simulations showed fewer water molecules around carboxylate groups across the whole hydration shells when more aromatic rings were present (Fig. 1d,e and Supplementary Figs. 10 and 11). Consequently, in *c*PIM-BP, water clusters seemed isolated from one another (Fig. 1f). By contrast, the hydrated phases in *c*PIM-Ph were mostly interconnected, whereas in *c*PIM-1 and *c*PIM-Et they were much larger and fully interconnected. Molecular dynamics simulations highlighted the crucial role of pendant group rigidity in controlling pore hydration. As visualized in Fig. 1e, the rigid phenyl and biphenyl segments in *c*PIM-Ph and *c*PIM-BP, along with the 1,2,4-oxadiazole linkages, created hydrophobic confined spaces near carboxylate groups, hindering water molecule access. By contrast, the flexible ethyl segment in *c*PIM-Et, which was capable of free rotation and bending, lowered the energy barrier for hydration (Supplementary Fig. 12). Collectively, these results indicate that the rigidity-reinforced hydrophobicity of pendant groups is the structural origin governing pore hydration.

**Fig. 2:**
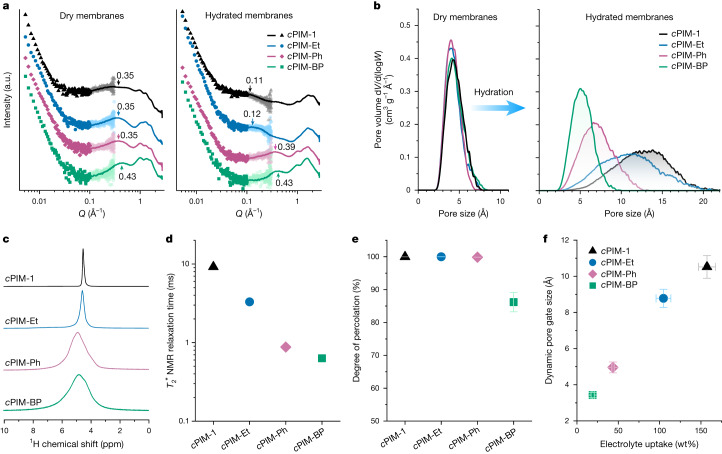
Characterization of pore structure evolution upon hydration. **a**, Small-angle and wide-angle X-ray scattering spectra of *c*PIM membranes. Symbols and lines represent small-angle and wide-angle X-ray scattering spectra, respectively. **b**, Pore size distribution in the dry and hydrated states derived from the simulated polymer models. **c**, ^1^H NMR spectra of water confined in membrane micropores. **d**, ${{T}}_{2}^{\ast }$ calculated from the full-width at half-maximum of water peaks in **c**. **e**, Degree of percolation calculated from hydrated polymer cells. **f**, Dynamic pore gate size of hydrated polymers. Error bars of the degree of percolation (**e**) and dynamic pore gate size (*y* axis in **f**) represent the standard deviation of five independent simulated polymer cells, and error bars of electrolyte update (*x* axis in **f**) represent the standard deviation of three or four independent samples. Electrolyte uptake was measured in 1 M aqueous KCl solution at 25 °C.

**Extended Data Fig. 1:**
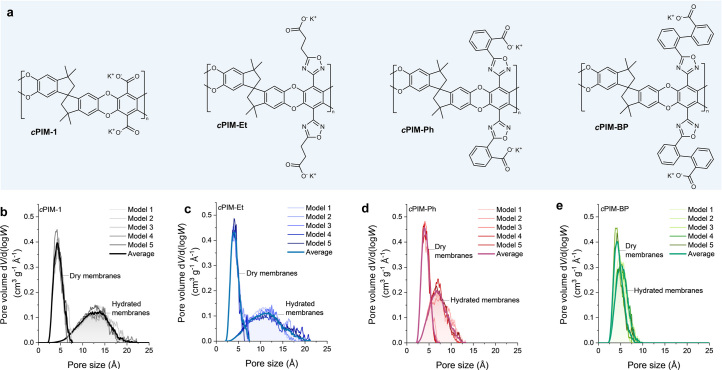
Pore size distributions. **a**, Chemical structures. **b**-**e**, Pore size distribution derived from simulated polymer boxes in the dry and hydrated states for *c*PIM-1 (**b**), *c*PIM-Et (**c**), *c*PIM-Ph (**d**), and *c*PIM-BP (**e**).

**Extended Data Fig. 2:**
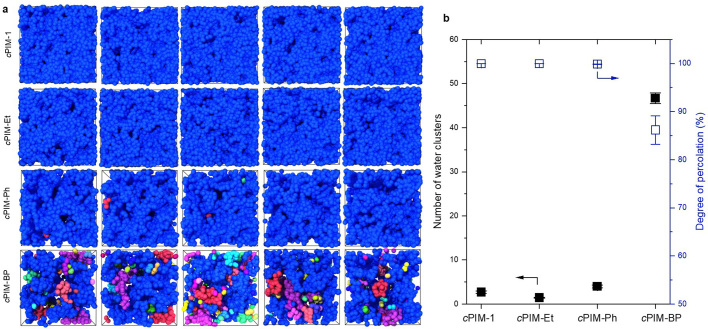
Water cluster analysis. **a**, Water molecules in simulated boxes of hydrated polymers with color-coding by interconnected cluster/pathway. Polymer chains are not shown in the simulation boxes for the sake of clarity. Water molecules are in different colors to show each water cluster. Two water molecules are considered in a connected pathway if the distance between their oxygen atoms is less than 3.5 Å. **b**, Degree of percolation refers to the fraction of water molecules in the largest interconnected water cluster/pathway.

We sought to understand how membrane pore structures evolved with varying hydration levels. Small-angle and wide-angle X-ray scattering spectra showed that regardless of the pendant group structure, all dry membranes exhibited a pronounced signal at a comparable momentum transfer (*Q*) of 0.35–0.43 Å^−^^1^, probably corresponding to the correlation length between membrane pores (Fig. 2a). Upon hydration, this signal shifted significantly for *c*PIM-1 and *c*PIM-Et but remained largely unchanged for *c*PIM-Ph and *c*PIM-BP, suggesting more significant pore swelling in the former. The associated broadening of this signal for *c*PIM-1 and *c*PIM-Et indicated increased polydispersity in pore size. Molecular dynamics simulations enabled the quantification of pore size distribution under both dry and hydrated conditions (Fig. 2b and Extended Data Fig. 1). In line with the X-ray scattering results, all dry *c*PIMs exhibited a similar pore size distribution ranging from 2 Å to 8 Å. After hydration, the distribution of *c*PIM-1 and *c*PIM-Et broadened and flattened, spanning from 3 Å to 20 Å, whereas *c*PIM-Ph and *c*PIM-BP underwent only slight pore enlargement, with most pores remaining in the subnanometre range. As quantified by the effective nuclear magnetic resonance (NMR) transverse relaxation time (${{T}}_{2}^{\ast }$), water molecules were subject to increased confinement in smaller pores (Fig. 2c,d). Analysis of interconnectivity among water clusters in hydrated polymer models indicated only partial percolation of the pore network in *c*PIM-BP, in contrast to the other *c*PIMs (Fig. 2e and Extended Data Fig. 2).

**Extended Data Fig. 3:**
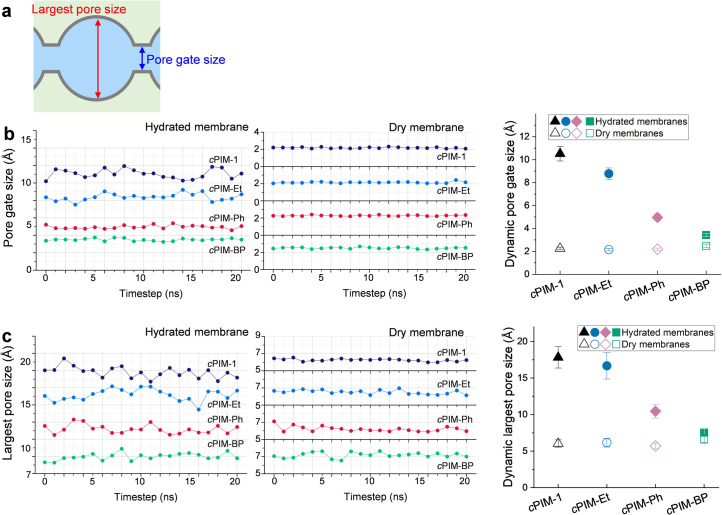
Dynamic pore size analysis. **a**, Schematic diagram showing the largest pore size and the pore gate size in the pore network of polymer membranes. **b**, Dynamic pore gate size. **c**, Dynamic largest pore size. Error bars represent the standard deviation of 5 independent simulation configurations.

Although pore size distribution provides insight into the porous structure of polymers, the bottlenecks or pore gates between interconnected micropores are the key structural feature that determine ion transport rate and selectivity (Fig. 1a). However, pore gate sizes are difficult to characterize using standard experimental techniques, whereas in molecular simulations, random segmental motions of polymer chains create transient pores that deviate from static models^27^. With these challenges in mind, we quantified the diameter of the largest probe capable of passing through the pore network in these polymer models by sampling over 20 ns of molecular dynamics in five independent configurations, defined as the dynamic pore gate size (DL_Gate_). This allowed us to capture a statistical representation of the polymer configurations as well as the dynamic flexibility of the systems (Fig. 2f and Extended Data Fig. 3). All dry polymers exhibited comparable DL_Gate_ values in the range of 2.2–2.5 Å. Upon hydration, DL_Gate_ increased more than fourfold to 10.5 ± 0.6 Å for *c*PIM-1 and 8.8 ± 0.5 Å for *c*PIM-Et but only slightly for *c*PIM-Ph (5.0 ± 0.3 Å). By contrast, DL_Gate_ in *c*PIM-BP remained largely unchanged at 3.4 ± 0.1 Å, close to its dry state. These quantitative analyses provide a comprehensive landscape of membrane porous structure and confirm effective control over membrane pore size upon hydration.

## Multiscale water and ion transport

**Fig. 3:**
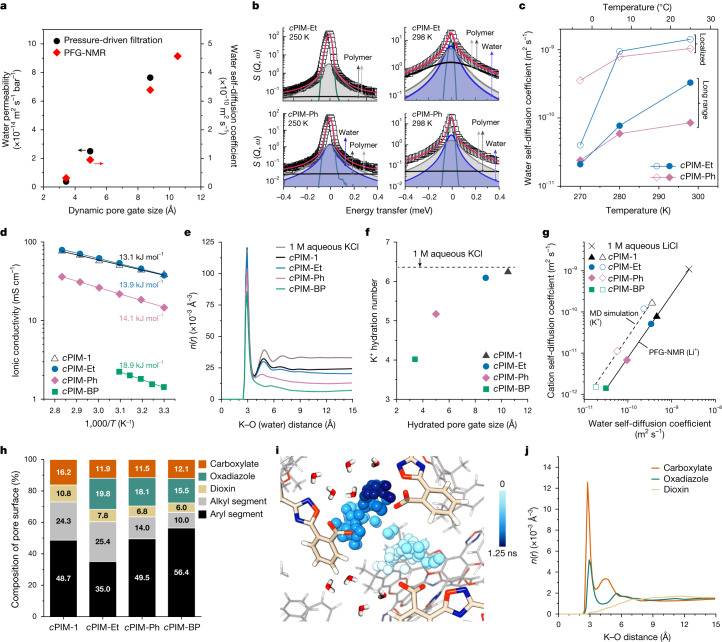
Water and ion transport. **a**, Macroscopic permeation and microscopic self-diffusion of water through membrane micropores. **b**, Analysis of QENS signal acquired with an energy resolution (*E*_res_) of 32.8 µeV. Data points (open squares) were fitted globally, shown as red lines, with the instrument resolution function in green. Polymer relaxation resulted in a broad Lorentzian contribution (grey shaded area) and a fast contribution (solid black lines). Water dynamics produced the main Lorentzian contribution with a narrower line width (light blue shaded area). **c**, Nanometric water self-diffusion as determined by QENS. **d**, Temperature-dependent ionic conductivity measured in 1 M aqueous KCl with activation energy labelled. *T* represents temperature. **e**, Modelled *n*(*r*) of K^+^ ions with respect to water molecules. **f**, Hydration number of K^+^ ions in the first hydration shell. **g**, Linear coupling of cation and water self-diffusion. Open symbols represent molecular dynamics simulation data with a timescale of 20 ns for polymers hydrated with 1 M aqueous KCl, whereas solid symbols represent those measured for 1 M aqueous LiCl by ^7^Li and ^1^H PFG-NMR, with observation times of 120 ms for Li^+^ ions and 30 ms for water. **h**, Relative percentages of each structural segment within 1 Å of the pore surface, calculated by atom count. **i**, K^+^ ion trajectory in *c*PIM-Ph over 1.25 ns of molecular dynamics simulation at 300 K, with K^+^ ions colour-coded by elapsed time. For clarity, some polymers, water molecules and ions are omitted (see Supplementary Fig. 26 for the original snapshot). **j**, Modelled *n*(*r*) between K^+^ ions and oxygen-containing components in *c*PIM-Ph.

**Extended Data Fig. 4:**
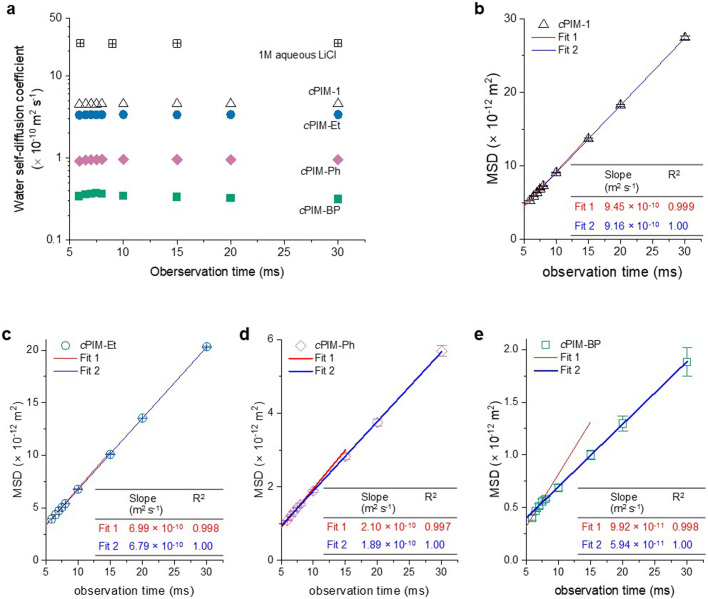
Water mobility revealed by PFG-NMR. **a**, Water self-diffusion coefficients as a function of observation time. **b**-**e**, Plots of mean squared displacement (MSD) versus observation time. Fits 1 and 2 correspond to linear fitting below and above the observation time of 8 ms, respectively. *c*PIM-BP exhibits a clear deviation from linearity between MSD and observation time within the studied timescale, suggesting increased diffusion restriction compared to other *c*PIMs. As PFG-NMR probes long-range diffusion, this greater restriction is likely the result of both smaller pore sizes and the deficiency in the interconnectivity of hydrated phases.

The *c*PIM series offers a unique platform for exploration of water transport and local motion across different membrane pore sizes. In pressure-driven filtration tests, the macroscopic water transport rate markedly decreased as DL_Gate_ was reduced (Fig. 3a and Supplementary Fig. 13). Similarly, pulsed field gradient (PFG) NMR spectroscopy showed a clear correlation between DL_Gate_ and microscopic water self-diffusion coefficients (Fig. 3a and Extended Data Fig. 4).

**Extended Data Fig. 5:**
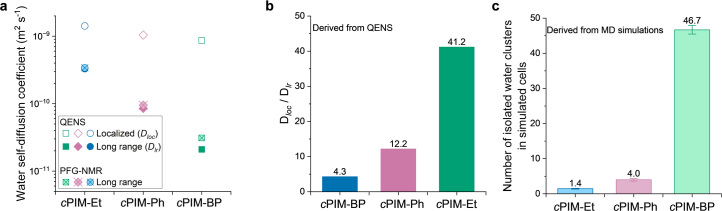
Water self-diffusion. **a**, Localized (*D*_*loc*_) and long-range (*D*_*lr*_) water self-diffusion. **b**, The ratio of *D*_*loc*_/*D*_*lr*_ as an indication of the degree of water cluster percolation. QENS data were acquired at 298 K on a picosecond timescale, and PFG-NMR data were obtained at 302.5 K with an observation time of 30 ms. **c**, Number of isolated water clusters in simulated boxes, derived from Extended Data Fig. 4. Pore size, however, may also influence the *D*_*loc*_/*D*_*lr*_ ratio. To elucidate this factor, we compared *D*_*loc*_ among *c*PIMs, as it directly reflects the degree of pore confinement. The variation in *D*_*loc*_ among the membranes is relatively small compared to the differences in the *D*_*loc*_/*D*_*lr*_. For instance, *D*_*loc*_ in *c*PIM-BP is only 39% lower than in *c*PIM-Et, yet the *D*_*loc*_/*D*_*lr*_ in *c*PIM-BP is nearly 10 times greater than that of *c*PIM-Et. This comparison confirms the direct correlation between *D*_*loc*_/*D*_*lr*_ ratio and water cluster interconnectivity.

To probe water mobility at the nanoscale, we used quasielastic neutron scattering (QENS) spectroscopy (Supplementary Figs. 14 and 15). QENS analysis (Fig. 3b and Supplementary Figs. 16–20) identified two types of water diffusive motion within *c*PIM membranes: localized (*D*_loc_) and long-range (*D*_lr_). *D*_loc_ is defined as the localized diffusion within a confined domain, influenced by the size and chemical environment of membrane pores, whereas *D*_lr_ represents diffusion between neighbouring domains. In *c*PIM-Ph, *D*_loc_ decreased gradually with temperature, in contrast to the sudden drop observed at 270 K for *c*PIM-Et (Fig. 3c and Supplementary Fig. 21). This greater temperature dependence in *c*PIM-Et also affected long-range water diffusion. We ascribe the faster water diffusion in *c*PIM-Et to the lower confinement within its larger hydrated phases, a difference that becomes more pronounced below the freezing point of bulk water. Notably, the nanometric long-range water diffusion coefficients identified by QENS at 298 K aligned closely with the micrometric ones measured by PFG-NMR (Extended Data Fig. 5), indicating consistent water diffusion across nanometric to micrometric scales within membrane hydrated phases.

The *D*_loc_/*D*_lr_ ratio reflects the barrier for water molecules moving across different hydrated domains within membrane pore networks. The low ratio for *c*PIM-Et (4.3) indicates a highly interconnected network of water clusters within its large pores. The ratio increased slightly to 12.2 for *c*PIM-Ph and further to 41.2 for *c*PIM-BP (Extended Data Fig. 5), suggesting reduced interconnectivity of water clusters, consistent with molecular dynamics simulation results (Fig. 2e).

Having quantitatively correlated water transport and membrane pore size, we next explored ion transport. Apparent ionic conductivity, measured by electrochemical impedance spectroscopy (EIS) in 1 M KCl electrolyte solutions, exhibited a positive correlation with membrane DL_Gate_ (Fig. 3d and Supplementary Figs. 22 and 23). The highest ionic conductivity was observed for *c*PIM-1 (38.4 mS cm^−1^ at 30 °C), whereas the lowest was for *c*PIM-BP (1.4 mS cm^−1^). Cation self-diffusion, measured by PFG-NMR, followed a similar trend (Supplementary Fig. 24). The Arrhenius-type temperature dependence of ionic conductivity demonstrated an activation energy of 18.9 kJ mol^−1^ for *c*PIM-BP, significantly higher than the 13.1–14.1 kJ mol^−1^ range for the other *c*PIMs. This probably stems from the greater partial dehydration as ions traverse the narrowly gated, poorly interconnected hydrated pores in *c*PIM-BP (Fig. 3e,f). The cation transference number, measured by linear sweep voltammetry, increased and approached unity as membrane DL_Gate_ decreased (Supplementary Table 2). Notably, the cation self-diffusion coefficient was directly proportional to that of water across the investigated pore size range, as demonstrated by PFG-NMR and confirmed by molecular dynamics simulations (Fig. 3g), indicating coupled diffusion of these mobile species within membrane pores through a water-mediated vehicular ion transport mechanism.

**Extended Data Fig. 6:**
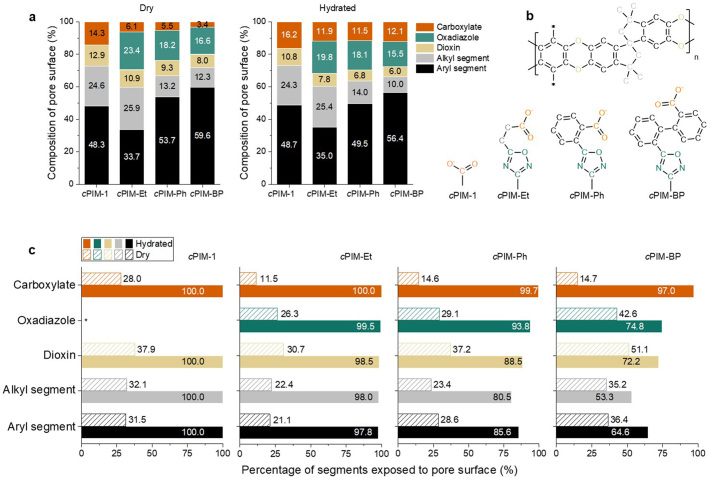
Chemical composition of pore surface. **a**, Relative percentage of each structural segment within 1 Å of the pore surface calculated by atom count. **b**, Chemical structures of *c*PIMs with atoms color-coded based on different segments. **c**, Percentage of structural segments exposed to pore surface versus those buried within the polymer matrix. Upon hydration, carboxylate groups and oxadiazole rings in *c*PIM-Ph and *c*PIM-BP re-orient towards the water-filled pores, while a portion of the backbones and pendant groups atoms remain buried within the matrix. In contrast, *c*PIM−1 and *c*PIM-Et lose their structural definition, showing no clear distinction between polymer matrix and pore surface.

To determine how ion–pore interactions influence ion transport under confinement, we quantified the chemical composition of pore surfaces and calculated RDFs of K^+^ ions surrounding each polar moiety of the polymers using molecular dynamics simulations. In hydrated polymer models, the relative percentage of carboxylates along the pore surface was consistent across *c*PIMs, except for *c*PIM-1 (Fig. 3h). However, in the dry state, a significant portion of carboxylates was buried within the polymer matrix (Extended Data Fig. 6). The hydration-driven rearrangement of polymer chain segments highlights the responsive nature of linear polymers, in contrast to the pore-filling mechanism in rigid framework materials. In essence, both carboxylate and oxadiazole groups contributed to K^+^ ion transport through electrostatic and dielectric interactions (Fig. 3i,j and Supplementary Fig. 25), creating a pore environment with finely tuned molecular interactions specific to cations. In *c*PIM-1 and *c*PIM-Et, ion–polymer coordination was more dynamic, with most K^+^ ions dissociating into the hydrated phases (Supplementary Fig. 11). By contrast, in *c*PIM-Ph, a greater portion of K^+^ ions were closely bound to carboxylates within the first hydration shell, forming ion pairs with reduced mobility, whereas bound K^+^ ions dominated in *c*PIM-BP. We attribute this ion pair formation to insufficient water for hydration and the lower dielectric constant of the hydrophobic pore surfaces^28,29^.

## Stable cycling of redox flow battery

The *c*PIM membranes, with tailored pore size, lend themselves to application in electrochemical devices requiring precise ion separation, exemplified here by redox flow batteries (RFBs). RFBs based on aqueous organic electrolytes are an emerging technology well suited to grid-scale energy storage owing to their distinct features of decoupled energy and power, abundant electrolyte materials and inherently safe operation^2,30^. In addition to providing fast ion conduction, ion-selective membranes are crucial for preventing undesired cross-over of subnanometre-sized redox-active species, a challenge that shortens battery lifespan and cannot be necessarily addressed by the traditional electrolyte rebalancing techniques^31^ commonly used in commercial vanadium RFBs.

**Fig. 4:**
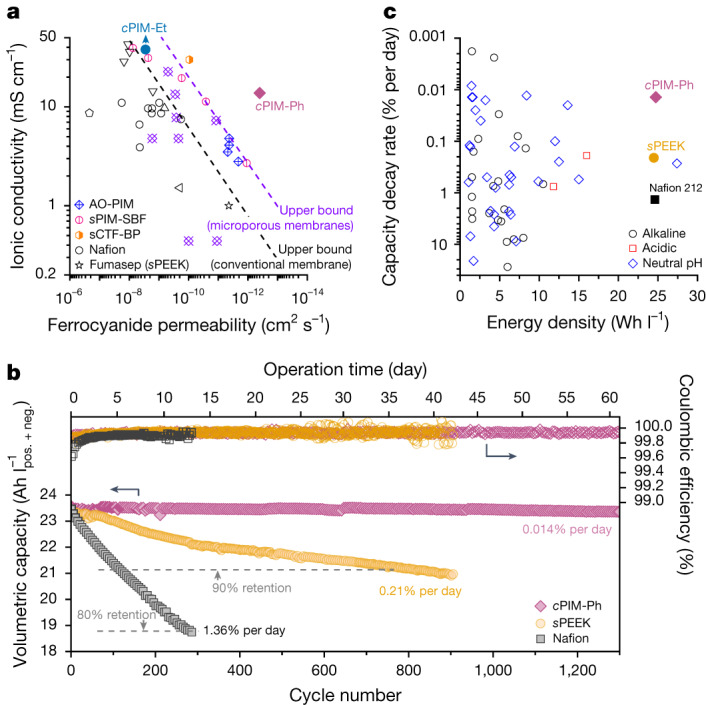
Stable cycling of energy-dense redox flow batteries. **a**, Upper bound plots of membrane ionic conductivity and ferrocyanide permeability (Supplementary Table 4). **b**, Cycling stability and coulombic efficiency of RFBs assembled with *c*PIM-Ph, Nafion 212 and *s*PEEK membranes. Catholyte: 6.67 ml mixed solution of 0.75 M K_4_Fe(CN)_6_ and 0.75 M Na_4_Fe(CN)_6_ in deionized water at pH 7; anolyte: 5 ml of 1.1 M 2,6-D2PEAQ in mixed supporting electrolyte of 0.5 M KCl and 0.5 M NaCl at pH 7. Volumetric capacity is based on the total volume of catholyte and anolyte. To access 100% depth of discharge and ensure accurate evaluation of decay rates, potentiostatic steps were added to each galvanostatic half cycle, with a current cutoff of 2 mA cm^−^^2^. **c**, Performance comparison of RFBs in **b** with those previously reported in the literature with respect to capacity decay rate and demonstrated energy density (Supplementary Table 6).

With an optimal pore gate size of 5.0 ± 0.3 Å, *c*PIM-Ph demonstrated both high ionic conductivity and ultralow permeability to representative redox-active molecules (Supplementary Table 3), in contrast to the far greater permeability of *c*PIM-Et, which had an 8.8 ± 0.5 Å gate size. Although the smaller pore size in *c*PIM-BP may offer even higher selectivity, its low ionic conductivity limits practical use in RFBs (for a discussion, see ‘Flow battery tests’ in Methods). The ion transport performance of *c*PIM-Ph greatly exceeds the upper bounds for both commercial ion-exchange membranes and microporous membranes reported in the literature (Fig. 4a).

**Extended Data Fig. 7:**
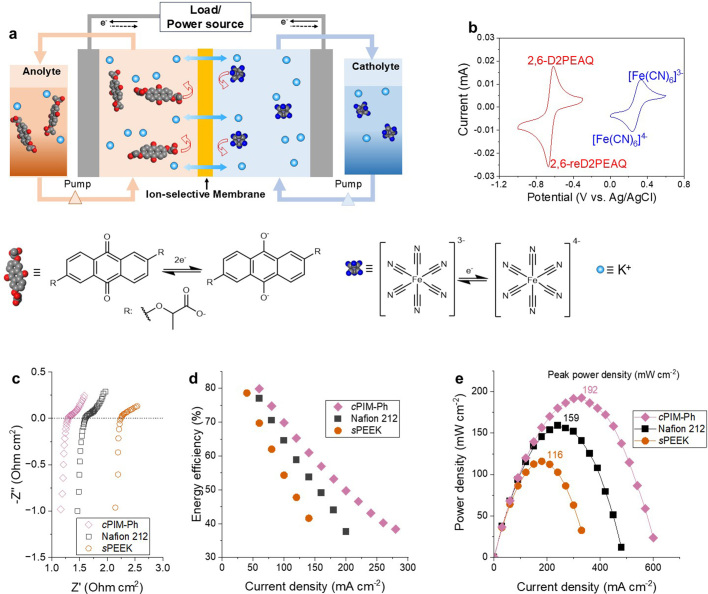
Redox flow battery. **a**, Schematic diagram showing key components of RFB. Ion-selective membranes allow fast conduction of small supporting ions for charge balancing, while blocking the undesired transport of redox-active species. Anions (Cl^−^) are not shown in the schematic. **b**, Cyclic voltammograms of 2,6-D2PEAQ and K_4_Fe(CN)_6_ redox couple. **c**, Electrochemical impedance spectra of RFB cells at 40 °C. **d**, Energy efficiency as a function of current density. **e**, Power density. Catholyte: 6.67 mL mixed solution of 0.75 M K_4_Fe(CN)_6_ and 0.75 M Na_4_Fe(CN)_6_ in deionized water at pH 7; anolyte: 5 mL 1.1 M 2,6-D2PEAQ in mixed supporting electrolyte of 0.5 M KCl and 0.5 M NaCl at pH 7.

To explore performance at the device level, we incorporated membranes into RFBs using 2,6-di-2-propionate ether anthraquinone (2,6-D2PEAQ) and ferrocyanide as the redox couple, chosen for their high solubility (exceeding 1 M) and stability^32^ (Extended Data Fig. 7). The *c*PIM-Ph membrane enabled stable cycling of the RFB with nearly capacity-balanced anolyte and catholyte, achieving a decay rate as low as 0.014% per operating day (Fig. 4b). The decay rate was 100 and 10 times lower than those observed in otherwise-identical RFBs with benchmark Nafion 212 and *s*PEEK membranes, respectively. Postmortem analysis confirmed that membrane cross-over was the primary mechanism leading to this difference (Supplementary Table 5). Combining high energy density and cycling stability, the *c*PIM-Ph-based RFB surpasses previously reported systems under similar conditions (Fig. 4c).

In conclusion, by manipulating the local hydrophobicity of polymer pendant groups, we synthesized microporous membranes with tailored pore gate sizes and chemical environments that promote specific polymer–ion interactions. These well-structured hydrated micropores enable effective control over the transport of water and ions, thereby providing both higher conductivity and selectivity than commercially available membranes and those previously reported in the literature. Our design approach may also be applicable to other functional materials targeting various challenging applications, such as aqueous and non-aqueous batteries, precise ion and chemical separations for water treatment, resource recovery, and circular economy^33^, in which pore swelling limits membrane performance, to achieve fit-for-purpose ionic and molecular sieving functions.

## Methods

### Synthesis of *c*PIMs

To an AO-PIM-1 solution (2 wt% in dimethyl sulfoxide) was added an anhydride in one portion (5 mol eq. relative to the repeating unit of AO-PIM-1). After full dissolution of the anhydride, the mixture was stirred at 30 °C for a further 4 h; then, potassium ethoxide was added (13 mol eq. relative to the repeating unit of AO-PIM-1). The mixture was vigorously stirred at room temperature for 1 h and then poured into 400 ml water. Hydrochloric acid was added dropwise to the solution until the pH reached 1–2. The precipitate was filtered, suspended in 0.5 M aqueous H_2_SO_4_ and heated to reflux for 4 h. The powder was collected by filtration, washed with deionized water and acetone, and briefly dried in air at 110 °C for 1 h to yield a free-flowing yellow powder. Extended drying was found to afford insoluble polymers, probably owing to anhydride formation among carboxylic acid groups.

This reaction was previously reported for synthesizing small drug molecules^34^ but had not been explored for polymer construction. When used in postpolymerization modifications, the reaction efficiency remained high under ambient conditions, with full conversion achieved in a few hours. Three commercially available, low-cost anhydrides (succinic anhydride, phthalic anhydride and diphenic anhydride) were used in the synthesis to attach ethyl-, phenyl- and biphenyl-containing pendant groups to the polymer backbones. More details are available in Supplementary Information.

### Membrane fabrication

Polymer powders were dissolved in dimethyl sulfoxide at concentrations of 4–10 wt% and centrifuged at 12,000 rpm for 10 min to remove insoluble impurities. Free-standing membranes were fabricated by casting polymer solutions on to glass plates or in glass Petri dishes, followed by solvent evaporation at 60 °C over 2 days. Polymer membranes were peeled off the glass substrates by immersion in water. Membranes in K^+^ ion form were obtained by deprotonation and ion exchange in 1 M aqueous KOH at room temperature overnight, followed by washing and immersion in deionized water or a suitable electrolyte solution three times, with each immersion lasting at least 6 h. Film thickness was measured with a micrometer.

For thin film composite (TFC) membranes, polymer solutions were prepared by dissolving polymer powders in tetrahydrofuran at concentrations of 0.5 or 3 wt% in an autoclave at 160 °C for 2 h. The solutions were dried over anhydrous MgSO_4_ and centrifuged at 12,000 rpm for 10 min to remove undissolved impurities. Porous polyacrylonitrile (PAN) ultrafiltration membranes were used as the substrate to provide mechanical support. To enhance hydrophilicity, PAN membranes were hydrolysed in 1 M KOH solution (H_2_O/EtOH, 1:1 by volume) for 1–2 h at 40 °C before use. TFC membranes were prepared by spin-coating 1 ml of polymer solution on to PAN membranes. This procedure was repeated once to ensure a defect-free surface morphology, resulting in a selective layer with a thickness of 70–100 nm for 0.5 wt% polymer solutions at 1,000 rpm and around 1 µm for 3 wt% polymer solutions at 500 rpm. The thickness of the selective layer was characterized by atomic force microscopy and scanning electron microscopy. TFC membranes fabricated with 0.5 wt% polymer solutions were used for pressure-driven water permeation tests, whereas those with 3 wt% *c*PIM-Ph solutions were used for cross-over tests. Before use, TFC membranes were pretreated in 1 M aqueous KOH at room temperature overnight for deprotonation and ion exchange of *c*PIM thin layers, followed by washing and immersion in deionized water or a suitable electrolyte solution three times, with each immersion time lasting at least 6 h.

### Gravimetric uptake and dimensional swelling ratio

Membrane samples were dried at 110 °C under vacuum for 12 h, quickly placed in a sealed glass vial and weighed with a high-precision analytical balance to obtain the dry mass. These samples were immersed in deionized water or an electrolyte solution at room temperature for 24 h. The mass of fully hydrated samples was measured after the excess surface water had been quickly wiped off with tissue paper. Water/electrolyte uptake was calculated according to equation (1):

1 $$\text{uptake}\,=\,\left[\frac{{W}_{{\rm{hydrated}}}}{{W}_{{\rm{dry}}}}-1\right]\times 100 \% $$

where *W*_hydrated_ and *W*_dry_ are the masses of fully hydrated and dry membrane samples, respectively. Hydration numbers of carboxylate groups were derived from the uptake normalized by ion-exchange capacity.

The linear swelling ratio in liquid electrolytes was determined from the difference in linear dimensions between the hydrated (*l*_hydrated_) and dry (*l*_dry_) free-standing membranes, measured using a micrometer, and was calculated according to equation (2):

2 $$\text{swelling ratio}\,=\,\left[\frac{{l}_{{\rm{hydrated}}}}{{l}_{{\rm{dry}}}}-1\right]\times 100 \% $$

Linear swelling ratios under different relative humidities were measured with a Semilab SE-2000 variable-angle spectroscopic ellipsometer within the spectral range of 248 nm to 1,653 nm in a controlled humidity chamber. Samples were prepared by spin-coating the polymer solution on to Au-coated silicon wafers to obtain a thickness of around 600 nm. All ellipsometry data were analysed with Semilab SEA software, using the Tauc–Lorentz and Gauss dispersion laws for optical model fitting. As ellipsometry measured the volumetric change of the membrane samples (*V*), the linear swelling ratio was calculated according to equation (3):

3 $$\text{swelling ratio}\,=\,\left(\sqrt[3]{\frac{{V}_{{\rm{hydrated}}}}{{V}_{{\rm{dry}}}}}-1\right)\times 100 \% $$

Hydration capacity is a thermodynamic property of polar and charged functionalities, and the overall membrane hydration is, in general, linearly proportional to the amount of such functional groups present in the polymer, while also being influenced by external salt concentration and temperature. Hence, various electrolyte concentrations and temperatures were deployed to evaluate gravimetric uptake and dimensional swelling.

### Ionic conductivity

The apparent through-plane ionic conductivity was measured by two-electrode EIS with an a.c. bias of 10 mV and a frequency range of 0.2 MHz to 10 Hz. Membrane samples were pretreated in 1 M aqueous KOH to fully deprotonate carboxylic acid groups and then soaked in 1 M aqueous KCl three times, with each immersion time lasting at least 6 h. The membranes were then sandwiched between two stainless steel electrodes and sealed with coin cells (type 2032), with extra electrolyte solution added. The assembled coin cells were placed in a temperature-controlled oven for conductivity measurement.

For highly conductive membranes with an areal resistance less than 2 Ω cm^2^ (*c*PIM-1, *c*PIM-Et, *c*PIM-Ph and pretreated Nafion), different layers of membranes were stacked to afford varied thickness, and the stacked membranes were subjected to EIS measurements^35,36^. The stack thickness was linearly fitted with areal resistance to derive the slope as the ionic conductivity, avoiding contributions to membrane resistance from contact and electrode resistance.

For membranes with an areal resistance greater than 2 Ω cm^2^ (*c*PIM-BP and as-received Nafion), the ionic conductivity was calculated on the basis of a single measurement according to the following equation:

4 $$\sigma =\frac{L}{({R}_{{\rm{m}}}-{R}_{0})\times A}$$

where *R*_m_ is the apparent resistance measured from Nyquist plots, *L* is the membrane thickness, *A* is the active membrane area (2.00 cm^2^) and *R*_m_ represents resistance from contact and electrode resistance (0.2 Ω cm^2^) measured from a shorted cell without membrane and verified by the stacking method.

### Apparent ion transference number

*V*–*I* curves were measured in an H-shaped cell using two Ag/AgCl electrodes (3.0 M KCl) and recorded with a potentiostat (Biologic SP-150). In the middle of the cell, a membrane was sandwiched using two O-rings to separate the two compartments. The apparent ion transference number (*t*) was calculated from the zero-current potential (*V*_0_), which is equal to the membrane potential, using the following equation:

5 $${V}_{0}=\left[\frac{{t}_{+}}{{z}_{+}}-\frac{{t}_{-}}{{z}_{-}}\right]\,\left[\frac{{k}_{{\rm{B}}}T}{e}\right]\,{\rm{l}}{\rm{n}}\,\left[\Delta C\frac{{\gamma }_{{\rm{h}}{\rm{i}}{\rm{g}}{\rm{h}}}}{{\gamma }_{{\rm{l}}{\rm{o}}{\rm{w}}}}\right]$$

where *k*_B_ is the Boltzmann constant, *z* is the ion charge number, *T* is the Kelvin temperature, *e* is the elementary charge, Δ*C* is the ratio of high concentration to low concentration (Δ*C* = 10), and *γ*_high_ and *γ*_low_ are the activity coefficients of the high-concentration solution and the low-concentration solution^37^, respectively.

### Cross-over

The cross-over of redox-active molecules was measured using stirred H-shaped cells. Membranes were sandwiched between two O-rings and placed in the middle of H-shaped cells with an effective membrane area of 1.54 cm^2^. Feed and permeate solutions were 0.1 M redox-active molecules dissolved in 1 M aqueous KCl (or KOH) solution and 1 M aqueous KCl (or KOH) solution, respectively. Constant stirring was applied to alleviate concentration polarization near membrane surfaces. The concentration change of the permeate solution for K_4_Fe(CN)_6_ cross-over was monitored by taking 0.1 ml aliquots to 9.9 ml 2 wt% aqueous HNO_3_ for inductively coupled plasma mass spectrometry (ICP-MS) measurements, whereas for the cross-over of organic molecules, aliquots were taken without dilution for ultraviolet–visible spectroscopy analysis and recycled back to the permeate solution. The permeation rate (flux) of ions and redox molecules across the membrane follows Fick’s first law:

6 $$J=\frac{V}{A}\left[\frac{\partial C}{\partial t}\right]$$

where *J* is the permeation rate (mol cm^−2^ s^−1^), *V* is the solution volume (ml), *A* is the effective membrane area (1.54 cm^2^), *C* is the concentration of the permeate solution (mol cm^−3^) and *t* is the diffusion time (s). As *C*_2_ ≪ *C*_1_ (hence *C*_1_ − *C*_2_ ≈ *C*_1_), under the assumption that boundary resistances next to the membrane are negligible, Fick’s first law can be simplified as:

7 $$J=\frac{p({C}_{1}-{C}_{2})}{l}=\frac{p{C}_{1}}{l}$$

where *p* is the permeability (cm^2^ s^−1^), *C*_1_ is the concentration of the feed solution (mol cm^−3^) and *l* is the membrane thickness (cm).

Free-standing membrane samples were used for cross-over tests with a typical membrane thickness of around 50 µm, except for *c*PIM-Et, which had a thickness of 120 µm. However, the cross-over rate for 50-µm-thick *c*PIM-Ph membranes was too slow to be detected within a reasonable testing period. For example, in the tests for K_4_Fe(CN)_6_ cross-over, the concentration on the permeate side remained below the ICP-MS detection limit even after 100 days. Although estimates based on equipment detection limits can provide a upper limit for cross-over rates, they lack accuracy. To address this, TFC membranes with a 1.1-µm-thick *c*PIM-Ph layer were fabricated for cross-over tests, allowing reliable quantification of the permeation rate within a reasonable timeframe. The results are summarized in Supplementary Table 3. Transport resistance from the porous PAN support in TFC membranes was negligible; for example, it exhibited a K_4_Fe(CN)_6_ permeation rate of 0.28 mmol l^−1^ h^−1^, several orders of magnitude faster than that of TFC membranes.

### Pressure-driven water permeation

Water permeation tests were performed using a dead-end stirred cell (Sterlitech) at various pressures in the range of 1–9 bar. The effective membrane area of the dead-end cell was 12.56 cm^2^. Before measurement of water flux under different pressures, a pressure of 20 bar was applied for at least 6 h until steady permeance was achieved. At least three independent TFC membrane samples were tested to confirm the reproducibility.

### NMR spectroscopy

NMR experiments were performed on a Bruker Avance III spectrometer equipped with a 7.0 T superconducting magnet operating at a ^1^H frequency of 300.13 MHz and at a sample temperature of 302.5 ± 0.3 K unless stated otherwise. ^1^H pulsed gradient stimulated echo (PGSTE) NMR was performed using a 5 mm ^1^H radiofrequency coil and ^7^Li PGSTE NMR using a 10 mm ^7^Li radio-frequency coil, both in a Bruker diff30 probe with a maximum gradient strength of 17.7 T m^−1^. ^1^H NMR spectra were acquired for each hydrated *c*PIM sample with a 10 kHz spectral width, four signal averages and a repetition time of 5 s. Chemical shifts were referenced externally to the water peak in a spectrum of 1 M LiCl in water, as this was sufficient for the intention of measuring the line widths, but chemical shift values may have varied owing to random drift during shimming and are not necessarily accurate. The ${{T}}_{2}^{\ast }$ relaxation times were calculated from the full width at half maximum values for each peak^38^, which were found by fitting to a Lorentzian function using dmfit software^39^.

#### Sample preparation

Polymer films were pretreated in 1 M aqueous LiOH overnight, followed by washing and immersion in 1 M aqueous LiCl more than three times, for at least 6 h each time. Fully hydrated polymer samples were quickly rolled up and placed into NMR glass tubes (Norell, 5 mm) after the excess surface water had been wiped off with tissue paper.

#### Self-diffusion coefficient

^1^H and ^7^Li PGSTE NMR experiments with bipolar gradients were used to measure the self-diffusion coefficients of water molecules and ions in hydrated polymer membrane samples. Of note, Li^+^ self-diffusion coefficients were measured, rather than K^+^, because of the relatively low receptivity, gyromagnetic ratio or sensitivity of ^39^K, ^40^K and ^41^K. Self-diffusion coefficients were calculated from a plot of signal intensity, *S*_G_, against the gradient strength, *G*, using the Stejskal–Tanner equation:

8 $${S}_{{\rm{G}}}={S}_{0}{{\rm{e}}}^{-{\gamma }^{2}{\delta }^{2}{G}^{2}D\left(\varDelta -\frac{\delta }{3}\right)}$$

where *S*_0_ is the signal intensity when *G* = 0, *γ* is the gyromagnetic ratio, *δ* is the gradient pulse length and *Δ* is the observation time between gradient pulses.

Typical parameters for ^1^H PGSTE NMR experiments were 16 gradient steps, spectral width (SWH) = 10,000 Hz, *δ* = 0.57 ms and a minimum of 28 signal averages. Typical 90° pulse lengths fell in the range 7.5–9.0 μs. Experiments were carried out over a range of observation times, *Δ* = 5.9, 6.5, 7.0, 7.5, 8.0, 10, 15, 20 and 30 ms. Typical parameters for ^7^Li PGSTE NMR experiments were eight gradient steps, SWH = 20,000 Hz and *δ* = 1 ms, with a minimum of 256 signal averages. Typical 90° pulse lengths were around 17.5 μs. Experiments were carried out at observation times of *Δ* = 8, 10, 12, 15, 20, 30, 50, 75, 100 and 120 ms. Maximum gradient strengths, *G*_max_, were chosen to minimize *S*_G_/*S*_0_. Error bars were determined from the average standard deviation of three repeats of experiments in which *Δ* = 10 and 50 ms. ^7^Li PGSTE NMR experiments were also performed on *c*PIM-Et and *c*PIM-Ph at temperatures 289.5, 293.5, 298.0, 302.5 and 306.5 ± 0.3 K. Eight gradient steps were used with *δ* = 1.5 ms, *Δ* = 30 ms and *G*_max_ = 17 T m^−1^, with SWH = 20,000 Hz and 256 signal averages.

To assess the presence of restricted diffusion, the MSD over time, ⟨[**r**′(*t*) − **r**(0)]^2^⟩, was determined from the measured diffusion coefficient, *D*, using the Einstein definition^40^

9 $$\langle {[{{\bf{r}}}^{{\prime} }(t)-{\bf{r}}(0)]}^{2}\rangle =2D\varDelta $$

and was plotted as a function of the observation time, *Δ*.

### Molecular simulation

All-atomistic molecular dynamics simulations were performed in the Large-scale Atomic/Molecular Massively Parallel Simulator^41^. Polymer and ion interactions were described by the OPLS-AA forcefield, and water was described by the TIP4P/EW model^42,43^. Water bonds and angles were restrained using the SHAKE algorithm^44^. A short-range cutoff of 12 Å was used for non-bonded interactions, and long-range coulombic interactions were implemented with the particle–particle particle–mesh technique. A timestep of 1.0 fs was used. The forcefield combination and equilibration scheme used in this work have been previously validated for ionic polymers, showing good agreement with experimental densities and X-ray scattering data^45,46^. The detailed procedure for construction of the polymer models is available in Supplementary Information.

#### Pore-size analysis

The pore networks formed in the dry and hydrated models were analysed using the Zeo++ package^47^. All structural analyses were the average of five independent models with frames captured every 1 ns over a total 20 ns of molecular dynamics. This was done to capture a statistical representation of polymer conformations as well as the dynamic flexibility of the hydrated models. For the hydrated models, water and ions were considered part of the mobile phase and were removed from each frame before geometric analysis. Pore size distributions were measured with a 1 Å probe and 60,000 Monte Carlo samples. The specific volume occupied by the polymer chains (*V*_vdW_) was determined by sampling the polymer box using a probe size of zero. Fractional free volume was calculated on the basis of the volume of the simulated box (*V*_box_) and *V*_vdW_ using the following equation^48^:

10 $$\text{fractional free volume}\,=\,1-1.3\frac{{V}_{{\rm{vdW}}}}{{V}_{{\rm{box}}}}$$

#### Degree of percolation

Water network characterization used the average of five independent models across 20 ns of molecular dynamics simulation sampled every 1 ns, using a distance-based clustering algorithm. Given the *xyz* coordinates of the water phase, any two water molecules were considered to be in an interconnected pathway if the distance between their oxygen atoms was within 3.5 Å. This distance was chosen to encompass the entire first peak in the oxygen–oxygen RDF for the TIP4P water model. We calculated both the average number of clusters in each system and the fraction of water molecules in the largest cluster. The degree of percolation was defined as the percentage of water molecules in the largest cluster over all water molecules.

#### Radial distribution functions

RDFs, *g*_ab_(*r*) between two groups of atoms, a and b, within the polymer models were calculated using the MDAnalysis package over trajectories of 20 ns with frames every 10 ps.

11 $${g}_{{\rm{ab}}}(r)={({N}_{\text{a}}{N}_{\text{b}})}^{-1}\mathop{\sum }\limits_{i=1}^{{N}_{\text{a}}}\mathop{\sum }\limits_{j=1}^{{N}_{\text{b}}}\langle \delta (| {r}_{i}-{r}_{j}| -r)\rangle $$

We also calculated the radial number density distribution function (*n*_ab_(*r*)) for a more direct comparison between systems with different numbers of atoms, where *ρ* is the number density of observed atoms:

12 $${n}_{{\rm{ab}}}(r)=\rho {g}_{{\rm{ab}}}(r)$$

#### Self-diffusion coefficient

Mean square displacement (MSD) was plotted every 10 ps over a trajectory of 20 ns. Self-diffusion coefficients (*D*_self_) were then extracted from the slope of the linear portion of the MSD according to the Einstein relation, where *d* is the dimensionality (in our case, 3), by the following equation:

13 $${D}_{{\rm{self}}}=\frac{1}{2d}\frac{\text{d}}{\text{d}t}\text{MSD}$$

#### Pore surface composition

To characterize the per-atom distribution along the pore surfaces, visual pore size distributions were generated using the Zeo++ package^47^ with a 1-Å probe, and the Euclidean distances between pore spheres and polymer atoms were calculated. Atoms located within 1 Å of any pore sphere were identified as pore surface atoms, whereas any outside this range were identified as being buried within the polymer matrix. This analysis was performed for a single snapshot; minor fluctuations will have occurred with motion of the systems.

### Neutron scattering

#### Fixed window scan

Fixed window scans (FWS) were acquired on BASIS (SNS, USA) from 30 to 333 K at a heating rate of 0.13 K min^−1^. The scattering signal was integrated at either Δ*E* = 0 (elastic; EFWS) or around an arbitrarily chosen energy range (inelastic; IFWS) with an integration width equivalent to *E*_res_.

EFWS can be used to identify the temperature at which relaxation processes become detectable within the spectroscopic timescale, indicated by a change in slope. Under the assumption of harmonic oscillations (*T* ≤ 100 K, for which the Debye–Waller approximation is valid), EFWS is also effective for calculating the temperature dependence of the MSD, ⟨*u*^2^⟩, of hydrogen atoms:

14 $$\frac{{I}_{{{\rm{inc}}}_{{\rm{elastic}}}}(Q,T)}{{I}_{{{\rm{inc}}}_{{\rm{elastic}}}}(Q,{T}_{\min })}=\exp \left[-\frac{1}{3}{Q}^{2}(\langle {u}^{2}\rangle {\langle {u}^{2}\rangle }_{{T}_{\min }})\right]$$

IFWS can differentiate between local (for example, rotational and/or nanoconfined) and diffusive motions. Local motions are characterized by *Q*-independent maxima in the inelastic intensity, whereas diffusive motions show *Q*-dependent maxima. IFWS analysis can also be used to estimate activation energy.

15 $${I}_{{\omega }_{{\rm{o}}{\rm{f}}{\rm{f}}}}^{{\rm{I}}{\rm{F}}{\rm{W}}{\rm{S}}}(T)\propto \frac{B}{{\rm{\pi }}}[1-{A}_{0}(Q)]\frac{\tau (T)}{1+{\omega }_{{\rm{o}}{\rm{f}}{\rm{f}}}^{2}\tau {(T)}^{2}}$$

16 $$\tau (T)\,={\tau }_{0}\exp \,\left[-\frac{{E}_{\text{A}}}{{k}_{{\rm{B}}}T}\right]$$

where *B* is a constant accounting for the resolution function, *ω*_off_ is the energy offset, *τ* is the relaxation time (with *τ*_0_ the high *T* limit), *A*_0_ is the elastic incoherent structure factor, *k*_B_ is the Boltzmann constant and *E*_A_ is the activation energy^49^.

#### Quasielastic neutron scattering

QENS profiles of *c*PIM-Et and *c*PIM-Ph were acquired at two facilities to capture dynamics across different timescales: (1) BASIS (SNS, USA)^50^ with an *E*_res_ of 3 μeV, covering the nanosecond timescale (~0.02 < *τ* < 2 ns); and (2) LET (ISIS, UK)^51^ to explore picosecond relaxation dynamics. The repetition rate multiplication method used with LET enabled simultaneous recording at three *E*_res_ values (14.6, 32.8 and 91.3 eV) with incident neutron energies of 1.03, 1.77 and 3.70 meV, respectively, covering the timescale range of ~1.5 < *τ* < 200 ps. By combining these timescales, a comprehensive relaxation profile was constructed to fully characterize the sample dynamics.

For *c*PIM-BP, QENS profiles were acquired on the IRIS spectrometer (ISIS, UK) using the PG002 analyser crystal set-up, which provided an energy resolution of 17.5 μeV and covered the momentum transfer range of 0.56 ≤ *Q* ≤ 1.84 Å^−1^, probing motions within the timescale range of 5 < *τ* < 100 ps. To ensure consistency across different instruments, QENS profiles of *c*PIM-Ph were also measured under similar conditions using IRIS, allowing for direct comparison with the BASIS and LET results.

The QENS signal appears as broadening in the energy transfer function due to relaxational and/or diffusional dynamics. Analysis of the scattering function, *S*(*Q, ω*), which reflects the time Fourier transform of the intermediate scattering function, *I*(*Q*, *t*), provides information on the static and dynamic correlations of different nuclei (*S*_coh_) and the spatiotemporal correlation between identical nuclei (*S*_inc_). The latter includes contributions from vibrational (*S*_v_), translational and/or diffusional (*S*_t_) and rotational/reorientation (*S*_r_) motions:

17 $${S}_{{\rm{inc}}}(Q,\omega )={S}_{{\rm{v}}}(Q,\omega ){\bigotimes S}_{{\rm{t}}}(Q,\omega )\bigotimes {S}_{{\rm{r}}}(Q,\omega )\bigotimes R$$

where *R* is the resolution function and is experimentally determined using a vanadium standard or sample at a base temperature of approximately 10 K on the assumption that dynamics are not detectable as all protons are in a quasistatic configuration and therefore contribute only to the elastic component. In isotropic cases, *S*_v_ becomes equal to ${{\rm{e}}}^{-\frac{1}{3}{Q}^{2}\langle {u}^{2}\rangle }$, where ⟨*u*^2^⟩ is the MSD accounting for vibrational excitations and proton delocalization occurring on timescales faster than the spectroscopical window.

Relaxation dynamics in QENS data are typically described by distinct Lorentzian functions and classified as accessible or non-accessible within a certain spectroscopic window. For accessible dynamics, translational and rotational components can be discriminated by the dispersive or non-dispersive behaviour, respectively, of the Lorentzian line width (*Γ*, half-width at half-maximum) as a function of *Q*^2^. Dynamics that exceed the instrument resolutions are classified as: (1) extremely fast dynamics, which produce an extraordinarily broad signal approximated by a relatively flat background function, *B*(*Q*); or (2) extremely slow dynamics (such as ‘immobile’ protons) incorporated within the elastic scattering signal [*δ*(*ω*)].

The Gaussian model describes molecular motion in a restricted geometry with ill-defined boundaries^52^ and is well suited to analysis of translational water diffusion within membrane hydrated pores. This model has been widely used in systems such as Nafion^53^ and polyamide^54^. Localized and long-range water diffusion coefficients (*D*_loc_ and *D*_lr_) in *c*PIM membranes were quantified on the basis of the following Gaussian model:

18 $${I}_{{\rm{F}}}(Q,t)=\exp \,\left[-{Q}^{2}{\sigma }^{2}(1-\exp \left(-\frac{{D}_{{\rm{l}}{\rm{o}}{\rm{c}}}t}{{\sigma }^{2}(1+2{D}_{{\rm{l}}{\rm{o}}{\rm{c}}}{Q}^{2}\tau )}\right))\right]\times \exp (\,-{D}_{{\rm{l}}{\rm{r}}}{Q}^{2}t)$$

where *τ* is the characteristic time of the local jump diffusion, and *σ* is the confinement domain size.

#### Sample preparation

Two isotopic contrasts, D_2_O and H_2_O, were used to disentangle water and polymer dynamics by fully hydrating membrane samples with each. In D_2_O-hydrated samples, only localized motions associated with polymer matrix dynamics were visible, enabling capture of polymer dynamics in the swollen state. Of note, for *c*PIM-Et, the high D_2_O content meant polymer dynamics acquired at high temperatures may also reflect D_2_O dynamics. In H_2_O-hydrated samples, both polymer dynamics and translational water dynamics were captured. Potassium-exchanged membranes were fully hydrated, pad-dried to remove surface water, and then stacked in four sheets. These sheets were quickly wrapped in aluminium foil and loaded into aluminium flat cells (4 cm × 5 cm). The aluminium cell, with an inner thickness of 0.5 mm, achieved around 90% neutron transmission. Indium was used to seal the cell. Scattering profiles were acquired between 200 and 333 K, covering a *Q* range of 0.3 to 2.1 Å^−^^1^. For normalization, complementary scattering profiles for the vanadium standard, an empty aluminium cell and samples at 5 K were also obtained. Data analysis was carried out on the *S*(*Q*, *ω*) spectra using Mantid^55^ and DAVE^56^. Data in the energy domain were analysed at fixed energy resolution, Fourier transformed to the time domain, scaled to obtain a unique relaxation profile, and analysed at fixed *Q* and temperature.

### Flow battery tests

Cell hardware (Scribner Associates) with POCO single serpentine pattern graphite plates was used to assemble the flow cells. A piece of membrane was sandwiched between electrodes with an effective geometric area of 7 cm^2^, comprising a stack of three sheets of carbon paper (Sigracet SGL 39AA). The remaining space between graphite plates was sealed with Viton gaskets. Electrolytes were fed into the cell at a flow rate of 40 ml min^−1^ through a Cole-Parmer peristaltic pump. All measurements were conducted in an argon-filled glovebox.

Carbon papers were pretreated by baking at 400 °C in the air for 24 h. Nafion 212 was pretreated following an established protocol^57^. Before full cell tests, membranes were soaked in 1 M aqueous KCl for 24 h. Membrane thicknesses were 45, 55 and 50 µm for *c*PIM-PA, Nafion 212 and sPEEK membranes, respectively, as measured in the hydrated state by a micrometer. The catholyte was prepared by dissolving 5 mmol K_4_Fe(CN)_6_ and 5 mmol Na_4_Fe(CN)_6_ in 6.7 ml deionized water. The anolyte was prepared by dissolving 5.05 mmol 2,6-D2PEAQ, 5.05 mmol KOH and 5.05 mmol NaOH in 5 ml mixed supporting electrolyte of 0.5 M KCl and 0.5 M NaCl. A trace amount of 1 M aqueous KOH was added to catholyte and anolyte solutions to adjust the pH to 7.0.

Galvanostatic cycling was performed at 40 °C with a constant current density of 80 mA cm^−2^, using cutoff voltages of 0.5 and 1.3 V. To access 100% depth of discharge and ensure accurate evaluation of decay rates, potentiostatic steps were added to each galvanostatic half cycle^58,59^, with a current cutoff of 2 mA cm^−2^. Data were recorded using a Biologic SP-150 potentiostat. After cycling, electrolyte aliquots were taken to quantify ferrocyanide cross-over, the capacity-limiting side, using ICP-MS. The degradation of redox-active molecules during cycling, which has been thoroughly investigated in previous studies^32,60,61^, was not explored in this work.

To evaluate rating performance, galvanostatic cycling was performed at varied current densities with cutoff voltages of 0.5 and 1.5 V at 40 °C. Electrochemical polarization curves were obtained by charging the cell to a desired state of charge and then polarizing using a linear galvanic sweep method at a rate of 200 mA s^−1^ from −6,000 to 6,000 mA at 40 °C. The corresponding power density at specific states of charge (20%, 50% and approximately 100%) was derived from the current–voltage curve. EIS was performed using a Biologic SP-150 potentiostat with an a.c. bias of 10 mV and a frequency range of 1 MHz to 100 Hz. Data were recorded using a Biologic VSP 300 potentiostat.

#### Suitability of *c*PIMs for acidic RFB systems

No tests in conventional acidic vanadium flow batteries were conducted in this work. The high p*K*_a_ of carboxylates (approximately 4) in *c*PIM membranes causes the loss of polymer charges in acidic environments, leading to excessive shrinkage of the designed pores, regardless of pendant group structures. This shrinkage falls outside the scope of our pore-size-tuning process. Although the resulting small pore size could enhance vanadium selectivity, the accompanying low proton conductivity represents a significant limitation of charge-neutral PIMs^62^.

#### Membrane resistance requirements

A previous techno-economic analysis^63^ suggested that membrane resistance needs to be below 1.5 Ω cm^2^ to ensure economical viability of flow battery systems. This resistance corresponds to a conductivity of 3.3 mS cm^−^^1^ assuming a membrane thickness of 50 μm. Consequently, pretreated Nafion membranes are predominantly used for flow batteries, including in this work to facilitate fair performance comparison and benchmarking (Supplementary Fig. 23). For the same reason, despite its potential for high selectivity, *c*PIM-BP is not suitable for RFB applications.

## Online content

Any methods, additional references, Nature Portfolio reporting summaries, source data, extended data, supplementary information, acknowledgements, peer review information; details of author contributions and competing interests; and statements of data and code availability are available at 10.1038/s41586-024-08140-2.

## Supplementary information

Supplementary Information Supplementary Methods, Figs. 1–26, Tables 1–6 and References.

## Data Availability

All data are available in the main text or Supplementary Information and also from the corresponding authors on reasonable request. The original QENS data are available at 10.5286/ISIS.E.RB2220214-1 and 10.5286/ISIS.E.RB2490083-1.

## References

[1] Uliana, A. A. et al. Ion-capture electrodialysis using multifunctional adsorptive membranes. *Science* **372**, 296–299 (2021). 33859036 10.1126/science.abf5991

[2] Park, M., Ryu, J., Wang, W. & Cho, J. Material design and engineering of next-generation flow-battery technologies. *Nat. Rev. Mater.* **2**, 16080 (2016).

[3] Jiao, K. et al. Designing the next generation of proton-exchange membrane fuel cells. *Nature* **595**, 361–369 (2021). 34262215 10.1038/s41586-021-03482-7

[4] Salvatore, D. A. et al. Designing anion exchange membranes for CO_2_ electrolysers. *Nat. Energy* **6**, 339–348 (2021).

[5] Park, E. J., Arges, C. G., Xu, H. & Kim, Y. S. Membrane strategies for water electrolysis. *ACS Energy Lett.* **7**, 3447–3457 (2022).

[6] Shen, J., Liu, G., Han, Y. & Jin, W. Artificial channels for confined mass transport at the sub-nanometre scale. *Nat. Rev. Mater.* **6**, 294–312 (2021).

[7] Kusoglu, A. & Weber, A. Z. New insights into perfluorinated sulfonic-acid ionomers. *Chem. Rev.* **117**, 987–1104 (2017). 28112903 10.1021/acs.chemrev.6b00159

[8] Shin, D. W., Guiver, M. D. & Lee, Y. M. Hydrocarbon-based polymer electrolyte membranes: importance of morphology on ion transport and membrane stability. *Chem. Rev.* **117**, 4759–4805 (2017). 28257183 10.1021/acs.chemrev.6b00586

[9] Budd, P. M. et al. Polymers of intrinsic microporosity (PIMs): robust, solution-processable, organic nanoporous materials. *Chem. Commun.*10.1039/B311764B (2004). 10.1039/b311764b 14737563

[10] Carta, M. et al. An efficient polymer molecular sieve for membrane gas separations. *Science* **339**, 303–307 (2013). 23329042 10.1126/science.1228032

[11] Baran, M. J. et al. Design rules for membranes from polymers of intrinsic microporosity for crossover-free aqueous electrochemical devices. *Joule* **3**, 2968–2985 (2019).

[12] Litt, M. & Wycisk, R. Poly(arylenesulfonic acids) with frozen-in free volume as hydrogen fuel cell membrane materials. *Polym. Rev.* **55**, 307–329 (2015).

[13] Adamski, M., Peressin, N. & Holdcroft, S. On the evolution of sulfonated polyphenylenes as proton exchange membranes for fuel cells. *Mater. Adv.* **2**, 4966–5005 (2021).

[14] Wang, A. et al. Ion-selective microporous polymer membranes with hydrogen-bond and salt-bridge networks for aqueous organic redox flow batteries. *Adv. Mater.* **35**, 2210098 (2023). 10.1002/adma.202210098 36634684

[15] Tan, R. et al. Hydrophilic microporous membranes for selective ion separation and flow-battery energy storage. *Nat. Mater.* **19**, 195–202 (2020). 31792424 10.1038/s41563-019-0536-8

[16] Ye, C. et al. Long-life aqueous organic redox flow batteries enabled by amidoxime-functionalized ion-selective polymer membranes. *Angew. Chem. Int. Ed.* **134**, e202207580 (2022). 10.1002/anie.202207580 PMC9541571 35876472

[17] Ye, C. et al. Development of efficient aqueous organic redox flow batteries using ion-sieving sulfonated polymer membranes. *Nat. Commun.* **13**, 3184 (2022). 35676263 10.1038/s41467-022-30943-y PMC9177609

[18] Zuo, P. et al. Near-frictionless ion transport within triazine framework membranes. *Nature* **617**, 299–305 (2023). 37100908 10.1038/s41586-023-05888-x PMC10131500

[19] Xu, W. et al. Sub-2-nm channels within covalent triazine framework enable fast proton-selective transport in flow battery membrane. *Adv. Funct. Mater.* **33**, 2300138 (2023).

[20] Lu, J. et al. Efficient metal ion sieving in rectifying subnanochannels enabled by metal-organic frameworks. *Nat. Mater.* **19**, 767–774 (2020). 32152561 10.1038/s41563-020-0634-7

[21] Liu, G. et al. Eliminating lattice defects in metal–organic framework molecular-sieving membranes. *Nat. Mater.* **22**, 769–776 (2023). 37169972 10.1038/s41563-023-01541-0

[22] Meng, Q.-W. et al. Enhancing ion selectivity by tuning solvation abilities of covalent-organic-framework membranes. *Proc. Natl Acad. Sci. USA* **121**, e2316716121 (2024). 38349874 10.1073/pnas.2316716121 PMC10895279

[23] Wang, H. et al. Covalent organic framework membranes for efficient separation of monovalent cations. *Nat. Commun.* **13**, 7123 (2022). 36402788 10.1038/s41467-022-34849-7 PMC9675805

[24] Xu, T. et al. Highly ion-permselective porous organic cage membranes with hierarchical channels. *J. Am. Chem. Soc.* **144**, 10220–10229 (2022). 35586909 10.1021/jacs.2c00318

[25] Fujisaki, H. et al. Selective methane oxidation by molecular iron catalysts in aqueous medium. *Nature* **616**, 476–481 (2023). 37020016 10.1038/s41586-023-05821-2

[26] Erdosy, D. P. et al. Microporous water with high gas solubilities. *Nature* **608**, 712–718 (2022). 36002487 10.1038/s41586-022-05029-w

[27] Koros, W. J. & Zhang, C. Materials for next-generation molecularly selective synthetic membranes. *Nat. Mater.* **16**, 289–297 (2017). 28114297 10.1038/nmat4805

[28] Matsumoto, H., Yamamoto, R. & Tanioka, A. Membrane potential across low-water-content charged membranes: effect of ion pairing. *J. Phys. Chem. B* **109**, 14130–14136 (2005). 16852774 10.1021/jp051585s

[29] Mafé, S., Ramírez, P., Tanioka, A. & Pellicer, J. Model for counterion-membrane-fixed ion pairing and Donnan equilibrium in charged membranes. *J. Phys. Chem. B* **101**, 1851–1856 (1997).

[30] Zhang, L., Feng, R., Wang, W. & Yu, G. Emerging chemistries and molecular designs for flow batteries. *Nat. Rev. Chem.* **6**, 524–543 (2022). 37118006 10.1038/s41570-022-00394-6

[31] Perry, M. L., Saraidaridis, J. D. & Darling, R. M. Crossover mitigation strategies for redox-flow batteries. *Curr. Opin. Electrochem.* **21**, 311–318 (2020).

[32] Kerr, E. F. et al. High energy density aqueous flow battery utilizing extremely stable, branching-induced high-solubility anthraquinone near neutral pH. *ACS Energy Lett.* **8**, 600–607 (2023).

[33] Sholl, D. S. & Lively, R. P. Seven chemical separations to change the world. *Nature* **532**, 435–437 (2016). 27121824 10.1038/532435a

[34] Baykov, S. et al. A convenient and mild method for 1,2,4-oxadiazole preparation: cyclodehydration of *O*-acylamidoximes in the superbase system MOH/DMSO. *Tetrahedron Lett.* **57**, 2898–2900 (2016).

[35] Cooper, K. R. Progress toward accurate through-plane ion transport resistance measurement of thin solid electrolytes. *J. Electrochem. Soc.* **157**, B1731 (2010).

[36] Díaz, J. C. & Kamcev, J. Ionic conductivity of ion-exchange membranes: measurement techniques and salt concentration dependence. *J. Membr. Sci.* **618**, 118718 (2021).

[37] Hamer, W. J. & Wu, Y. C. Osmotic coefficients and mean activity coefficients of uni-univalent electrolytes in water at 25 °C. *J. Phys. Chem. Ref. Data* **1**, 1047–1100 (1972).

[38] Bloembergen, N., Purcell, E. M. & Pound, R. V. Relaxation effects in nuclear magnetic resonance absorption. *Phys. Rev.* **73**, 679–712 (1948).

[39] Massiot, D. et al. Modelling one-and two-dimensional solid-state NMR spectra. *Magn. Reson. Chem.* **40**, 70–76 (2002).

[40] Callaghan, P. T. *Translational Dynamics and Magnetic Resonance: Principles of Pulsed Gradient Spin Echo NMR* (Oxford Univ. Press, 2011).

[41] Thompson, A. P. et al. LAMMPS—a flexible simulation tool for particle-based materials modeling at the atomic, meso, and continuum scales. *Comp. Phys. Commun.* **271**, 10817 (2022).

[42] Jorgensen, W. L., Maxwell, D. S. & Tirado-Rives, J. Development and testing of the OPLS all-atom force field on conformational energetics and properties of organic liquids. *J. Am. Chem. Soc.* **118**, 11225–11236 (1996).

[43] Horn, H. W. et al. Development of an improved four-site water model for biomolecular simulations: TIP4P-Ew. *J. Chem. Phys.* **120**, 9665–9678 (2004). 15267980 10.1063/1.1683075

[44] Ryckaert, J.-P., Ciccotti, G. & Berendsen, H. J. Numerical integration of the cartesian equations of motion of a system with constraints: molecular dynamics of *n*-alkanes. *J. Comput. Phys.* **23**, 327–341 (1977).

[45] Buitrago, C. F. et al. Direct comparisons of X-ray scattering and atomistic molecular dynamics simulations for precise acid copolymers and ionomers. *Macromolecules* **48**, 1210–1220 (2015).

[46] Sorte, E. G. et al. Impact of hydration and sulfonation on the morphology and ionic conductivity of sulfonated poly(phenylene) proton exchange membranes. *Macromolecules* **52**, 857–876 (2019).

[47] Willems, T. F., Rycroft, C. H., Kazi, M., Meza, J. C. & Haranczyk, M. Algorithms and tools for high-throughput geometry-based analysis of crystalline porous materials. *Microporous Mesoporous Mater.* **149**, 134–141 (2012).

[48] Hart, K. E. & Colina, C. M. Estimating gas permeability and permselectivity of microporous polymers. *J. Membr. Sci.* **468**, 259–268 (2014).

[49] Frick, B., Combet, J. & Van Eijck, L. New possibilities with inelastic fixed window scans and linear motor Doppler drives on high resolution neutron backscattering spectrometers. *Nucl. Instrum. Methods Phys. Res. Sect. A* **669**, 7–13 (2012).

[50] Mamontov, E. & Herwig, K. W. A time-of-flight backscattering spectrometer at the Spallation Neutron Source, BASIS. *Rev. Sci. Instrum.*10.1063/1.3626214 (2011). 10.1063/1.3626214 21895277

[51] Bewley, R., Taylor, J. & Bennington, S. LET, a cold neutron multi-disk chopper spectrometer at ISIS. *Nucl. Instrum. Methods Phys. Res. Sect. A* **637**, 128–134 (2011).

[52] Perrin, J.-C., Lyonnard, S. & Volino, F. Quasielastic neutron scattering study of water dynamics in hydrated nafion membranes. *J. Phys. Chem. C* **111**, 3393–3404 (2007).

[53] Berrod, Q., Hanot, S., Guillermo, A., Mossa, S. & Lyonnard, S. Water sub-diffusion in membranes for fuel cells. *Sci. Rep.* **7**, 8326 (2017). 28827621 10.1038/s41598-017-08746-9 PMC5567110

[54] Foglia, F., Frick, B., Nania, M., Livingston, A. G. & Cabral, J. T. Multimodal confined water dynamics in reverse osmosis polyamide membranes. *Nat. Commun.* **13**, 2809 (2022). 35589719 10.1038/s41467-022-30555-6 PMC9120036

[55] Arnold, O. et al. Mantid—data analysis and visualization package for neutron scattering and μSR experiments. *Nucl. Instrum. Methods Phys. Res. Sect. A* **764**, 156–166 (2014).

[56] Azuah, R. T. et al. DAVE: a comprehensive software suite for the reduction, visualization, and analysis of low energy neutron spectroscopic data. *J. Res. Natl Inst. Stand. Technol.* **114**, 341–358 (2009). 27504233 10.6028/jres.114.025 PMC4646530

[57] Lin, K. et al. Alkaline quinone flow battery. *Science* **349**, 1529–1532 (2015). 26404834 10.1126/science.aab3033

[58] Brushett, F. R., Aziz, M. J. & Rodby, K. E. On lifetime and cost of redox-active organics for aqueous flow batteries. *ACS Energy Lett.* **5**, 879–884 (2020).

[59] Kwabi, D. G., Ji, Y. & Aziz, M. J. Electrolyte lifetime in aqueous organic redox flow batteries: a critical review. *Chem. Rev.* **120**, 6467–6489 (2020). 32053366 10.1021/acs.chemrev.9b00599

[60] Fell, E. M. et al. Long-term stability of ferri-/ferrocyanide as an electroactive component for redox flow battery applications: on the origin of apparent capacity fade. *J. Electrochem. Soc.* **170**, 070525 (2023).

[61] Hu, M., Wang, A. P., Luo, J., Wei, Q. & Liu, T. L. Cycling performance and mechanistic insights of ferricyanide electrolytes in alkaline redox flow batteries. *Adv. Energy Mater.* **13**, 2203762 (2023).

[62] Chae, I. S. et al. Ultra-high proton/vanadium selectivity for hydrophobic polymer membranes with intrinsic nanopores for redox flow battery. *Adv. Energy Mater.* **6**, 1600517 (2016).

[63] Dmello, R., Milshtein, J. D., Brushett, F. R. & Smith, K. C. Cost-driven materials selection criteria for redox flow battery electrolytes. *J. Power Sources* **330**, 261–272 (2016).

