# Supplementary material for: Selective ion transport through hydrated micropores in polymer membranes
Source: Nature. 2024 Nov 6;635(8038):353–8. doi: 10.1038/s41586-024-08140-2 (PMC11560840; doi:10.1038/s41586-024-08140-2)
Supplement: Supplementary file 1 — Supplementary Methods, Figs. 1–26, Tables 1–6 and References. [file 41586_2024_8140_MOESM1_ESM.pdf]

nature portfolio

https://doi.org/10.1038/s41586-024-08140-2

Supplementary information

Selective ion transport through hydrated micropores in polymer membranes

In the format provided by the authors and unedited

Supplementary Information for

Selective ion transport through hydrated micropores in polymer membranes

Anqi Wang[1,9]*, Charlotte Breakwell[2], Fabrizia Foglia[3], Rui Tan[1], Louie Lovell[4], Xiaochu Wei[1], Toby Wong[1], Naiqi Meng[1], Haodong Li[1], Andrew Seel[5,6], Mona Sarter[5], Keenan Smith[3], Alberto Alvarez-Fernandez[7], Mate Furedi[7], Stefan Guldin[7], Melanie M. Britton[4], Neil B. McKeown[8], Kim E. Jelfs[2], Qilei Song[1]*

[1]Department of Chemical Engineering, Imperial College London; London, SW7 2AZ, UK.
[2]Department of Chemistry, Molecular Sciences Research Hub, Imperial College London; London, W12 0BZ, UK.
[3]Department of Chemistry, University College London; London, WC1H 0AJ, UK.
[4]School of Chemistry, University of Birmingham; Birmingham B15 2TT, UK.
[5]ISIS Neutron and Muon Source, Science & Technology Facilities Council, Rutherford Appleton Laboratory, Harwell Science and Innovation Campus, Chilton, UK.
[6]Department of Physics, Royal Holloway University of London; Egham TW20 0EX, UK.
[7]Department of Chemical Engineering, University College London; London, WC1E 7JE, UK.
[8]EaStCHEM, School of Chemistry, University of Edinburgh; Edinburgh, EH9 3FJ, UK
[9]Present address: Physical Science and Engineering Division, King Abdullah University of Science and Technology; Thuwal, 23955, Saudi Arabia

*Corresponding author. Email: anqi.wang16@imperial.ac.uk, q.song@imperial.ac.uk

Supplementary Methods

1. General methods and equipment

Commercially available reagents were used without further purification unless otherwise noted. 3,3′,3′-tetramethyl-1,1′- spirobisindane-5,5′,6,6′-tetrol was purified by recrystallization in THF/Hexane. 2,3,5,6-tetrafluoroterephthalonitrile was purified by recrystallization in methanol/water. PIM-1, AO-PIM-1 and *c*PIM-1 were synthesized following procedures reported elsewhere[15,64]. *s*PEEK (IEC of 1.5 meq g^{-1}) was synthesized following procedures reported elsewhere[65]. Potassium ethoxide was stored and weighed in an Argon-filled glove box.

Fourier-transform infrared spectroscopy (FT-IR) was performed on a Perkin-Elmer Spectrum 100 FTIR spectrometer with polymer membranes mounted on a zinc–selenium/diamond plate.

Liquid state ^{1}H and ^{13}C nuclear magnetic resonance (NMR) spectra were collected using Bruker Avance III HD 600 MHz or Jeol 400 MHz spectrometers at 25 °C and 60 °C for samples in $CDCl_3$ and *d*-DMSO, respectively. 4 vol.% TFA was added to shift the water peak in ^{1}H NMR.

Small-angle and wide-angle X-ray scattering (SAXS and WAXS) was performed on a Ganesha 300XL (SAXSLAB) instrument, equipped with a high brilliance microfocus Cu source (λ = 1.54 Å). SAXS and WAXS patterns were recorded using a Pilatus 300 K solid-state photon-counting detector with a 2 mm beam stop with a sample-to-detector distance of 1041 mm for SAXS and 101 mm WAXS. The beam center and the sample-to-detector distance were calibrated using the position of diffraction peaks from a standard silver behenate powder. SAXS and WAXS patterns were radially averaged around the direct beam position using SAXSGUI software. Film trips fully hydrated with 1M aqueous KCl were held in sealed capillary tubes.

Tensile tests of dry membrane strips were carried out using a Lloyd-Ametek EZ50 Material Testing Machine at room temperature and relative humidity of ~ 50% at a rate of 10 % min^{-1}.

Surface and cross-section morphology of membrane and electrode samples was characterized by scanning electron microscopy (SEM) using a Karl Zeiss LEO 1525 microscope. Before testing, the membranes were manually fractured in liquid nitrogen and coated with a thin layer of chromium.

Atomic force microscopy (AFM) was carried out with Bruker Scanasyst Dimension Icon AFM facility using a peak force tapping mode in ambient air.

Skeletal densities of polymer films were measured using a Micromeritics Accupyc II 1340 helium pycnometer equipped with a 3.5 cm^3 sample chamber at 25 °C. Each sample was degassed at 110 °C under vacuum for 12 h before measurement. A cycle of 10 measurements was performed to derive the mean value and standard deviation.

Low-pressure gas physisorption was performed using a Micromeritics 3Flex surface characterization analyzer. Each sample was degassed at 110 °C under vacuum for 12 h, and then loaded into the apparatus and in situ degassed at 110 °C for another 12 h. Nitrogen sorption isotherms were measured at 77 K, and CO_2 sorption isotherms were measured at 273 K.

Water vapor sorption was performed using (1) an IGA-001 gravimetric analyzer (Hidden Isochema) at 30 °C and (2) a DVS analyzer (Surface Measurement Systems) at 40 °C.

In crossover tests, metal ion concentration was detected by inductively coupled plasma mass spectrometry (ICP-MS) with aliquots diluted in 2 wt.% HNO_3. UV–Vis spectra were measured using a UV–Vis spectrometer (UV-1800, Shimadzu) at a wavelength interval of 0.5 nm.

2. Synthesis of model compound

3,13-dicyanobenzo-1,2,4',5'-bis(1,4-benzodioxane) (DCBD)

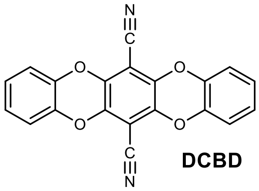

DCBD

Anhydrous DMF (120 mL) was added to 1,2-dihydroxybenzene (4.50g, 40.7 mmol), tetrafluoro-terephthalonitrile (3.71g, 18.5 mmol) and anhydrous potassium carbonate (16.8g, 122 mmol) under N_2. The mixture was heated to 65 ^{o}C for 24 h and then poured into water. The precipitate was collected by filtration, washed with water and acetone, and dried at 110 ^{o}C under vacuum to afford DCBD (6.36 g, 100%) as a yellow powder. ^{1}H NMR (600 MHz, $CDCl_3$): δ (ppm) 7.09-6.98 (m, 8H). ^{13}C NMR (150 MHz, $CDCl_3$): δ (ppm) 143.3, 140.7, 135.7, 124.6, 116.3, 112.5.

3,13-diamidoximebenzo-1,2,4′,5′-bis(1,4-benzodioxane) (DABD)

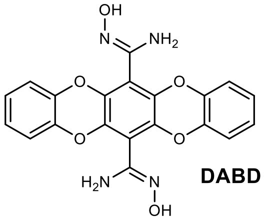

DABD

Hydroxylamine aqueous solution (50 wt.%, 45mL, 734 mmol) was added to a suspension of DCBD (3.08 g, 9.1 mmol) in THF (300 mL) under N_2 and the mixture was heated to 70 ^{o}C for 24 h. After cooling to room temperature, the precipitate was filtered, washed with THF and water, and dried at 110 ^{o}C under vacuum to afford DABD (3.62g, 98%) as a pale-yellow powder. ^{1}H NMR (600 MHz, $(CD_3)_2SO$): δ (ppm) 9.53 (s, 2H), 7.00-6.97 (m, 4H), 6.94-6.91 (m, 4H), 5.88 (s, 4H).

3,3'-((2,3,5,6-bisbenzodioxane-1,4-phenylene)-bis(1,2,4-oxadiazole-3,5-diyl))dipropionic acid (DABD-Et)

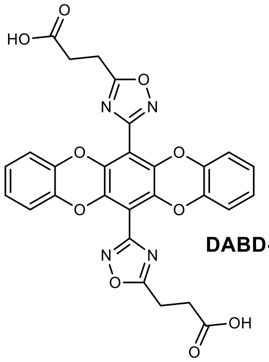

DABD-Et

DMSO (20 mL) was added to DABD (0.60 g, 1.48 mmol) and succinic anhydride (0.64 g, 6.4 mmol). The mixture was stirred at room temperature for 3 h and potassium ethoxide (1.06g, 12.6 mmol) was added to the solution. The mixture was vigorously stirred for 1.5 h and then poured into 400 mL water. Hydrochloric acid was added dropwise to adjust the pH to 1-2. The precipitate was filtered, washed with water, and dried at 110 ^{o}C under vacuum to afford a cream-color powder (0.76g, 91%). ^{1}H NMR (600 MHz, $(CD_3)_2SO$): δ (ppm) 12.48 (br s, 2H), 6.99 (s, 4H), 6.86 (s, 4H), 3.29 (s, 4H) and 2.89 (s, 4H). ^{13}C NMR (150 MHz, $(CD_3)_2SO$): δ (ppm) 179.9, 172.7, 160.0, 140.1, 136.1, 125.1, 116.5, 106.6, 30.0 and 21.7.

3. Synthesis of *c*PIMs

*c*PIM-Et

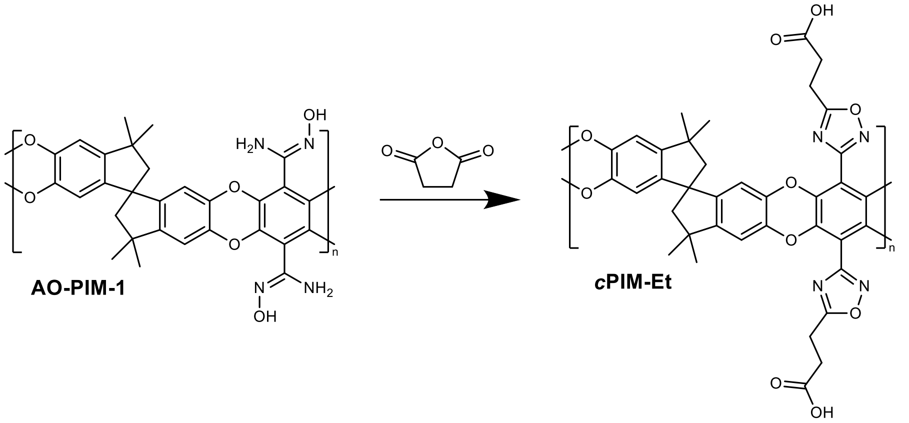

General procedure was followed using AO-PIM-1 (2.00 g, 3.8 mmol repeat unit), succinic anhydride (1.9 g, 19 mmol), potassium ethoxide (4.15 g, 49.4 mmol), DMSO (100 mL) to afford *c*PIM-Et (2.54 g, 92%) as a yellow powder. ^{1}H NMR (400 MHz, $(CD_3)_2SO$): δ (ppm) 6.65 (s, 2H), 6.15 (s, 2H), 2.90 (m, 4H), 2.81 (m, 4H), 2.24 (s, 2H), 2.06 (s, 2H) and 1.23 (d, 12H). ^{13}C NMR (150 MHz, $(CD_3)_2SO$): δ (ppm) 179.4, 172.2, 159.8, 148.4, 145.4, 139.6, 135.9, 111.0, 106.2, 58.3, 56.5, 42.7, 30.8-29.3, and 21.6

*c*PIM-Ph

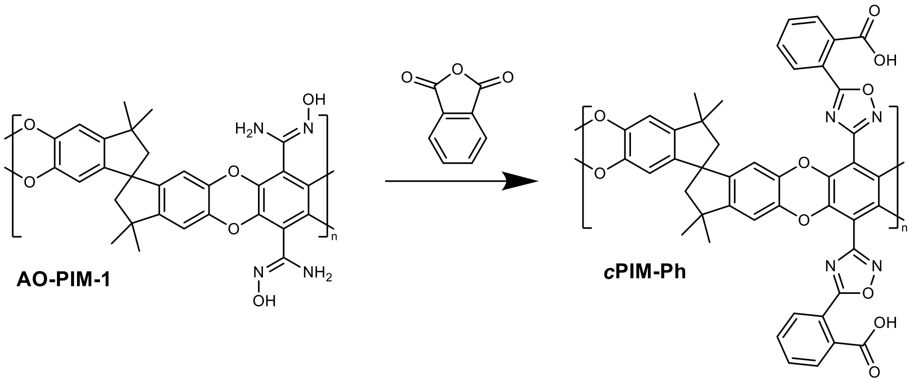

General procedure was followed using AO-PIM-1 (2.00 g, 3.8 mmol repeat unit), phthalic anhydride (2.8 g, 19 mmol), potassium ethoxide (4.15 g, 49.4 mmol), DMSO (100 mL) to afford *c*PIM-Ph (2.84 g, 91%) as a light-yellow powder. ^{1}H NMR (400 MHz, $(CD_3)_2SO$): δ (ppm) 8.10-7.66 (m, 8H), 6.72 (s, 2H), 6.22 (s, 2H), 2.23 (s, 2H), 2.11 (s, 2H) and 1.24 (d, 12H). ^{13}C NMR (150 MHz, $(CD_3)_2SO$): δ (ppm) 205.8, 175.5, 167.3, 160.3, 148.3, 145.4, 139.6, 136.0, 133.5, 132.4, 131.2, 130.1, 129.3, 122.4, 111.1, 109.9, 106.1, 58.3, 56.5, 42.7, 30.8, 30.3 and 29.3

cPIM-BP

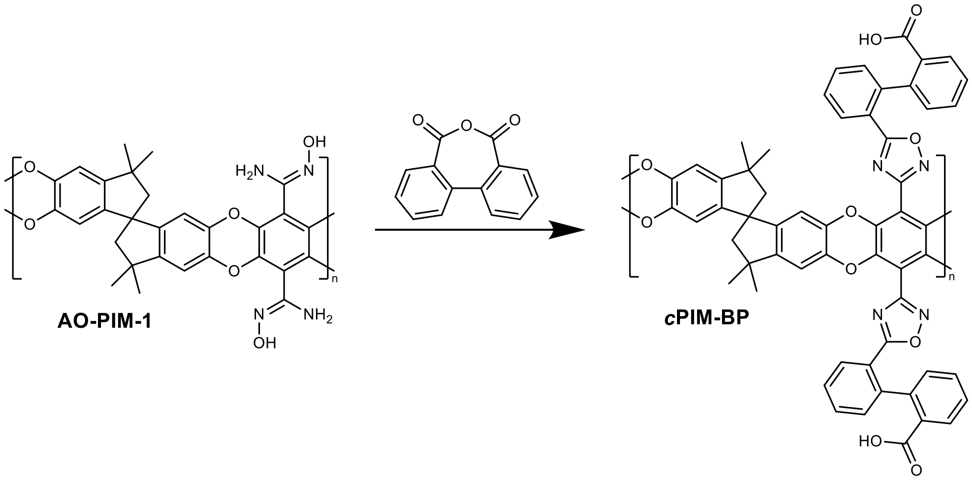

General procedure was followed using AO-PIM-1 (2.00 g, 3.8 mmol repeat unit), diphenic anhydride (4.256 g, 3.8 mmol), potassium ethoxide (4.15 g, 49.4 mmol), DMSO (100 mL) to afford *c*PIM-BP (3.44 g, 93%) as a light-yellow powder. ^{1}H NMR (400 MHz, $(CD_3)_2SO$): δ (ppm) 9.78-7.23 (m, 16H), 6.68 (s, 2H), 6.26 (s, 2H), 2.26 (s, 2H), 2.12 (s, 2H) and 1.25 (d, 12H). ^{13}C NMR (150 MHz, $(CD_3)_2SO$): δ (ppm) 205.8, 176.1, 167.2, 159.9, 148.4, 145.5, 142.3, 140.6, 139.4, 135.7, 130.5, 129.5, 129.1, 127.4, 122.5, 111.2, 109.9, 106.1, 58.3, 56.6, 42.8, 30.8, 30.3 and 29.2

4. Synthesis of 2,6-di-2-propionate ether anthraquinone (2,6-D2PEAQ)

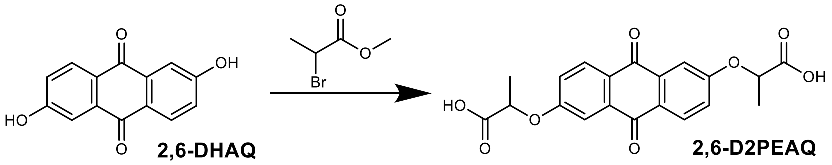

The synthesis procedure is adapted from the literature[32]. Anhydrous DMF (60 mL) was added to 2,6-dihydroxyanthraquinone (20g, 83.3 mmol), methyl 2-bromopropionate (41.7 g, 250 mmol, 3 mol eq.) and anhydrous potassium carbonate (92 g, 122 mmol, 8 mol eq.) under N_2. The mixture was heated to 60 ^{o}C for 1 h and then poured into water. The precipitate was collected by filtration and washed with water to yield sandy yellow powder. The collected powder was suspended in 1M KOH solution in water and 2-propanol (1:1 by volume). The mixture was heated to 80 ^{o}C for 3 hours and filtered to remove undissolved particles. 1M aqueous HCl was added to the dark red liquor until pH = 1-2. The yellow precipitate was collected by vacuum filtration, washed with water and methanol, and dried at 110 ^{o}C under vacuum overnight to afford 2,6-D2PEAQ [23 g, 72%, Lit.[32] 57%]. ^{1}H NMR (400 MHz, $CDCl_3$): δ (ppm) 8.15 (d, 2H), 7.53 (d, 2H), 7.38 (dd, 2H), 5.14 (t, 2H) and 1.59 (d, 6H). ^{13}C NMR (100 MHz, $(CD_3)_2SO$): δ (ppm) 181.6, 172.5, 163.1, 135.9, 130.0, 127.6, 121.7, 112.0, 73.2 and 18.5.

5. Procedure for the construction of polymer models

i. A polymer chain of 2 repeating units was first optimized in Gaussian 16 (Ref[66]) at the Hartree–Fock (HF) theory level using the 6-31G(d) basis set. Single-point energy calculations were then performed using the Møller–Plesset second-order perturbation (MP2) method with the correlation-consistent polarized valence cc-pVTZ(-f) basis set and partial charges extracted using an electrostatic surface potential methodology (CHelpG). These were readjusted for the carboxylate group to ensure a net charge of exactly -*e*.

ii. Representative monomer geometries were extracted from the middle of the optimized polymer chain and 150 monomer units were packed randomly inside a periodic box at a low density (~0.2 – 0.3 g cm^{-3}). 5 initial starting configurations were generated for each system.

iii. The previously published Polymatic simulated polymerization algorithm was then used to generate long polymer chains inside the box by creating new bonds between monomer units within an 8 Å cut-off from one another in between energy minimization and cycles of NVT MD[67]. During the polymerization, artificial charges were placed on 'reactive' atom types (± 0.3 *e*) to increase bias towards bond formation. These were removed after each bond formation. The partial charges on carboxylate group atoms were also set to 0 during the polymerization so as not to obstruct chain growth by repulsive interactions between negatively charged monomer units. The partial charges were readded to carboxylate groups upon completion of the polymerization scheme using a Python script.

iv. Following the polymerization, any unreacted chain ends were saturated with a capping group (in this case, a hydroxy group or a fluorine atom) to mirror the experimental precursors, with one K^+ ion inserted per carboxylate group for charge neutrality. At this point, for the hydrated models, water molecules, K^+ and Cl^- ions were randomly inserted into the voids around the polymer chains. The amount of water and ions added was based on experimental electrolyte uptakes.

v. The models were then compressed to a density ~0.6 g cm^{-3} over 600 ps of NVT MD at 300K. We then followed the equilibration scheme set out by Abbott and Frischknecht[68] which included a 21-step MD protocol of NVT and NPT cycles to gradually compress and decompress the system to an experimentally comparable density, followed by a further 13-step thermal annealing in the NVT ensemble. Finally, long NVT production runs were carried out for 20 ns at 300 K with frames saved every 10 ps.

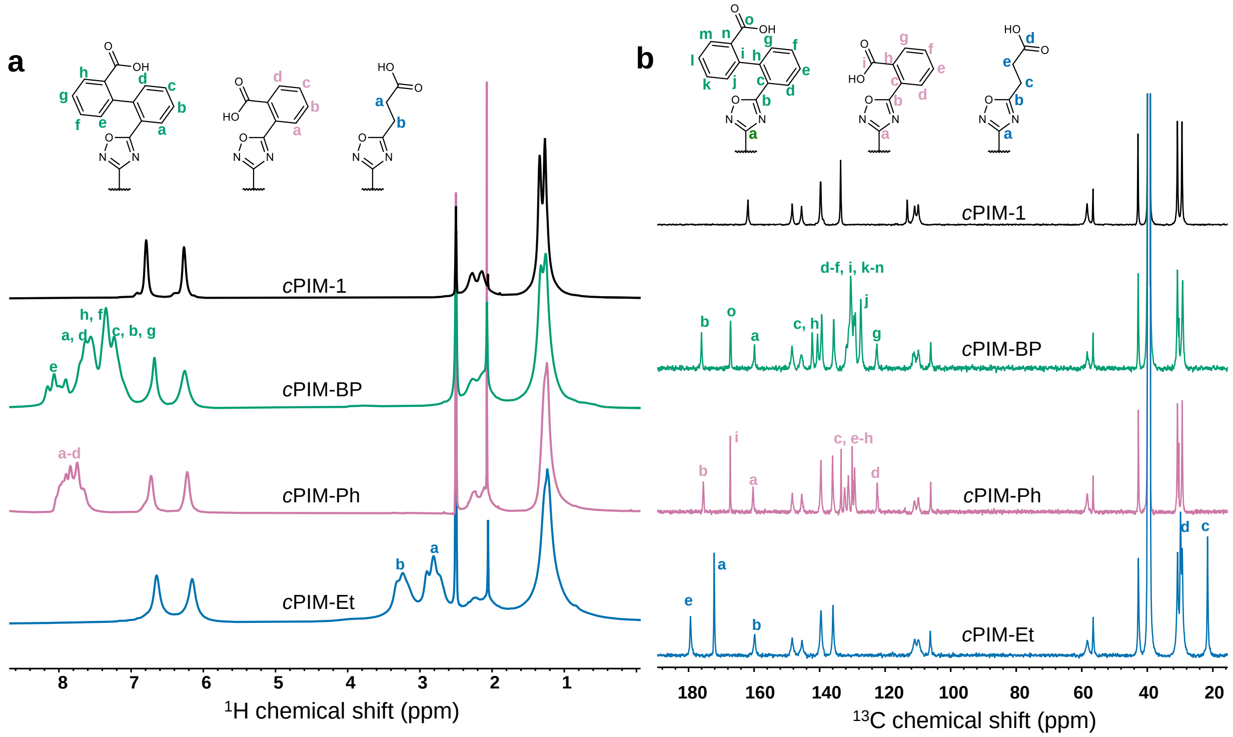

Supplementary Fig. 1 | NMR spectra. **a**, ^{1}H NMR spectra. **b**, ^{13}C NMR spectra. Peak assignment of pendant groups is labeled in the figures, while the polymer main chain assignment is omitted for the sake of clarity and can be referenced in the literature[14]. NMR spectra were recorded in *d*-DMSO at 60 ^{o}C. To shift the water peak in ^{1}H NMR, 4 vol.% TFA was added to polymer solutions, while a neat solution without TFA was used for collecting ^{13}C NMR spectra. Chemical shifts were recorded in parts per million and corrected according to solvent peak (2.50 for ^{1}H and 39.51 for ^{13}C).

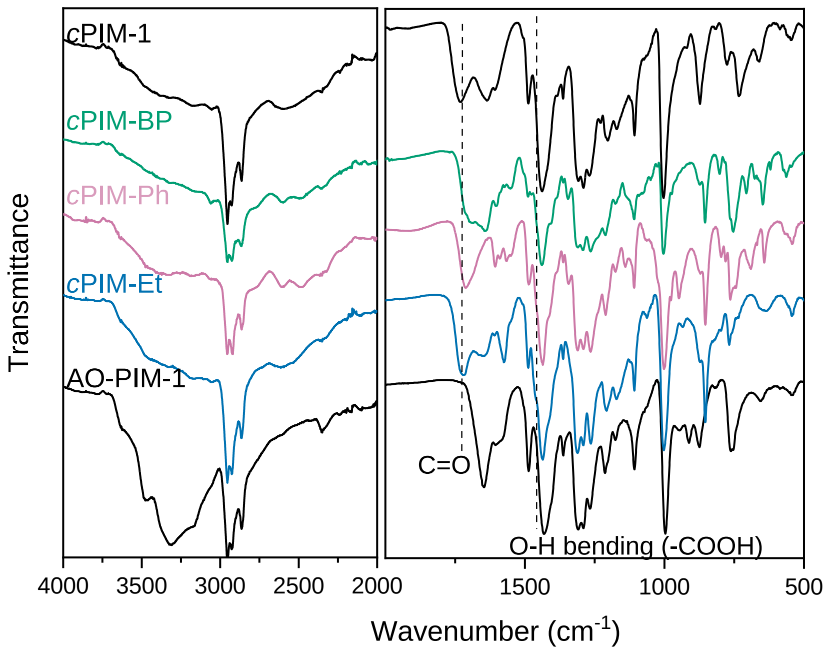

Supplementary Fig. 2 | FT-IR spectra. Characteristic peaks corresponding to carboxylate groups are labeled in the figure. The spectrum of AO-PIM-1, the precursor polymer for making *c*PIMs, was also added for comparison.

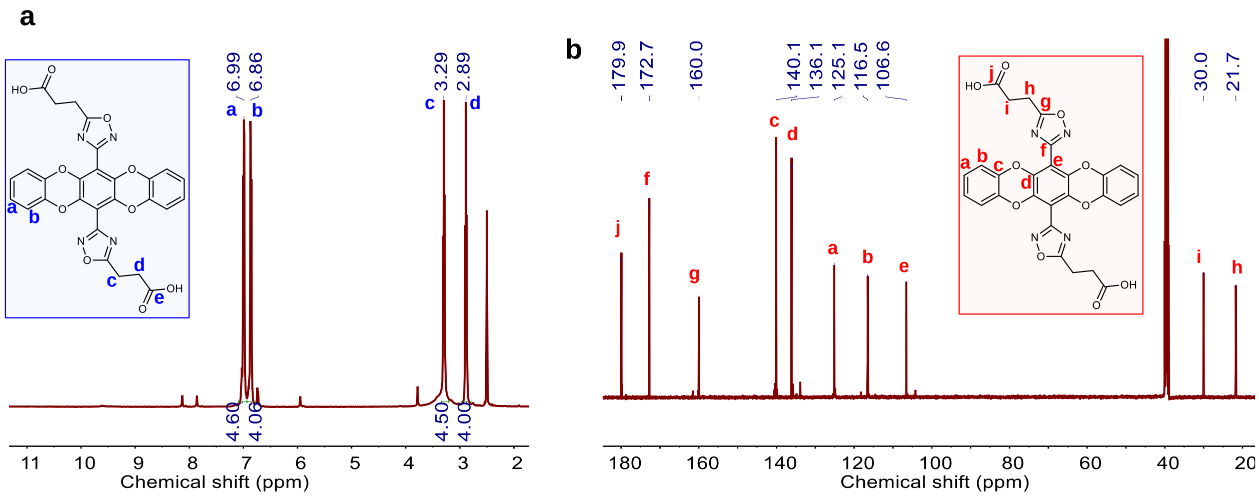

Supplementary Fig. 3 | Synthesis and characterization of model compound DABD-Et. **a**, ^{1}H NMR spectra, and **b**, ^{13}C NMR spectra. Peak assignment is labeled in the figures.

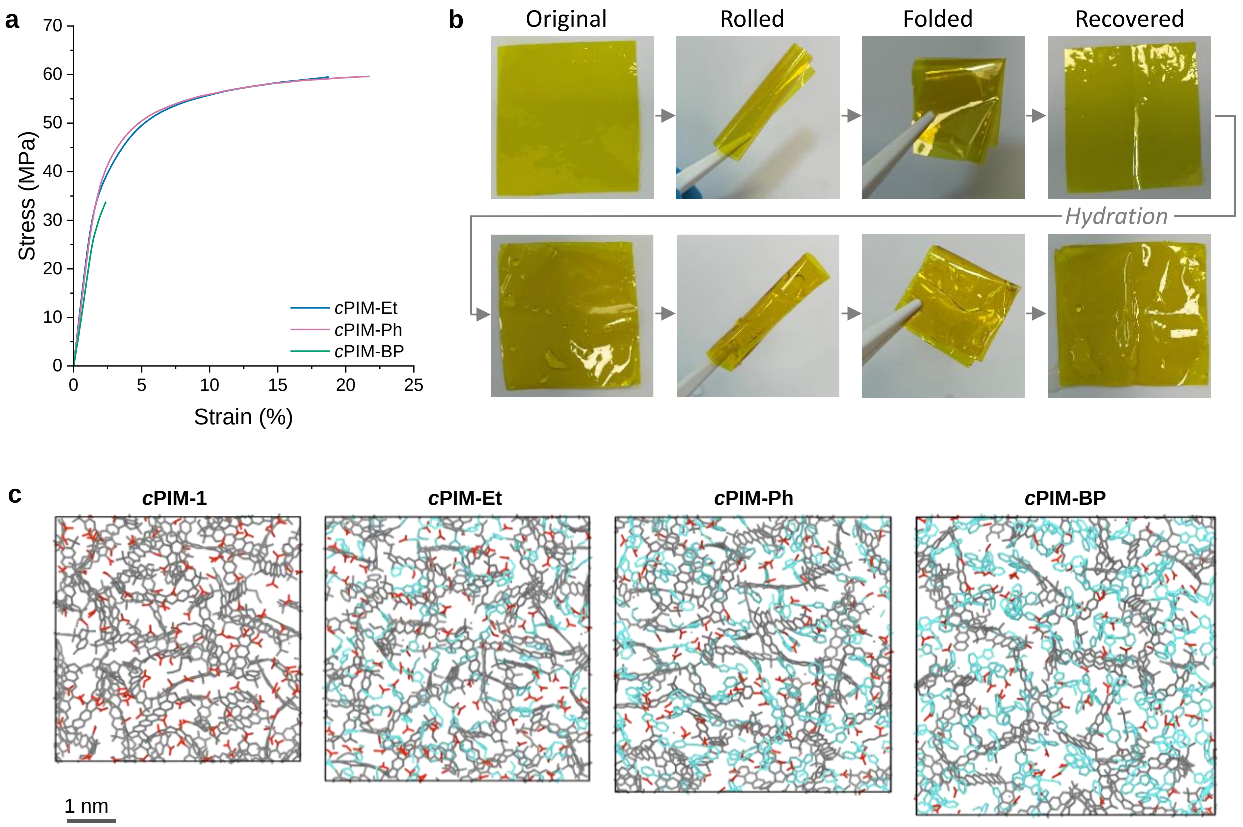

Supplementary Fig. 4 | Mechanical properties. a, Tensile tests of dry films. **b**, Images showing membrane mechanical robustness during rolling and folding tests. **c**, 2 nm-thick cross sections of the simulated polymer models in the dry state. The chain backbone, pendant groups, and carboxylates are highlighted in grey, cyan, and red, respectively. *c*PIM-Et and *c*PIM-Ph were mechanically robust, with an ultimate tensile strength of around 60 MPa, while *c*PIM-BP was brittle. The brittleness of *c*PIM-BP is still under investigation, but simulation results in **c** suggest a larger intersegment distance in *c*PIM-BP, which may indicate inhibited polymer chain entanglement due to the bulky biphenyl-containing pendant groups. The variation in box dimensions is due to the different atom numbers per repeat unit among the *c*PIMs.

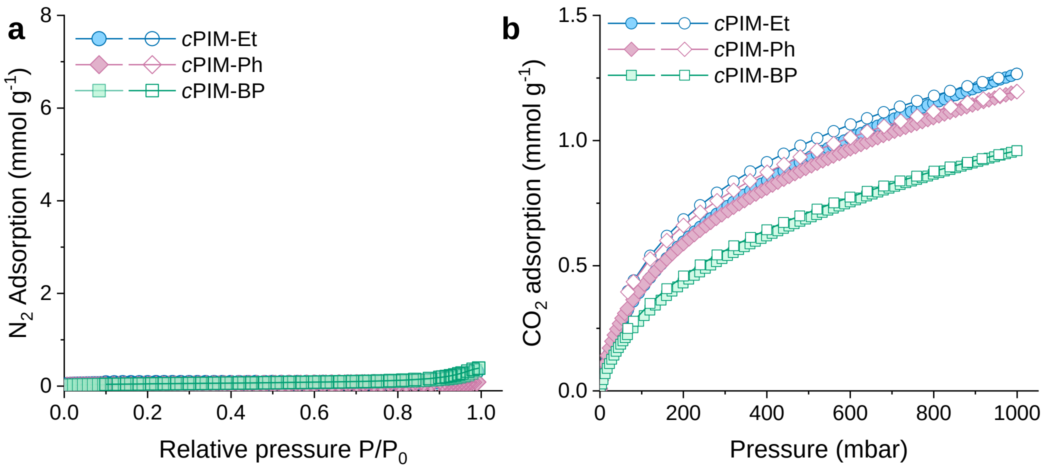

Supplementary Fig. 5 | Gas physisorption. **a**, N_2 sorption isotherms at 77 K and **b**, CO_2 sorption isotherms at 273 K were measured for K^+ ion-exchanged *c*PIM samples. K^+ ion-exchanged *c*PIM-1 was not included in the measurement due to difficulties in sample preparation. Specifically, soaking in DI water to remove residual salts introduced during ion exchange led to the full dissolution of K^+ ion-exchanged *c*PIM-1, a challenge that could potentially be mitigated by using dialysis bags but was not explored in this work. Sorption isotherms of AO-PIM-1 and *c*PIM-1 are available in previous studies[14].

N_2 sorption and Brunauer–Emmett–Teller (BET) surface area analysis are standard methods to characterize porous materials used in sorption, catalysis, and gas separation. However, a new paradigm favors porous materials with smaller pore sizes and lower pore interconnectivity, advantageous in challenging separations, such as isotope separation[69], crude oil fractionation[70], and redox flow batteries. N_2 sorption fails to characterize these materials as their porosity becomes inaccessible to N_2 at 77 K, while CO_2 sorption at 273 K is more effective because of CO_2's smaller size and higher operating temperature.

Indeed, *c*PIMs show minimal N_2 sorption but reasonably high CO_2 sorption capacity. CO_2 sorption capacity of *c*PIMs is much higher than that of non-porous glassy polymers and about half that of conventional PIMs[9,10,71] due to the bulkiness of pendant groups and intermolecular interactions that lead to tighter chain packing[72]. The sorption behavior of *c*PIMs is similar to that of *N*-Aryl–linked spirocyclic polymers[70], previously reported to have "non-interconnected" microporosity.

Supplementary Table 1 | Physical properties of *c*PIM membranes. Error bars represent standard derivations of 5 independent simulation configurations and 10 experimental measurements.

Membrane[1]	IEC (meq g^{-1})	Skeletal Density (g cm^{-3})		Simulated fractional free volume (FFV)	Electrolyte uptake (wt.%)[2]	Hydration number[3], λ
		Simulated	Experimental			
*c*PIM-1	3.5	1.16 ± 0.02	-	0.194 ± 0.010	157.3 ± 10.2	23.4 ± 1.5
*c*PIM-Et	2.6	1.20 ± 0.02	1.1825 ± 0.0018	0.200 ± 0.006	104.6 ± 9.0	20.8 ± 1.8
*c*PIM-Ph	2.3	1.20 ± 0.01	1.2263 ± 0.0038	0.208 ± 0.007	43.5 ± 1.9	9.7 ± 0.4
*c*PIM-BP	2.0	1.13 ± 0.03	1.1153 ± 0.0034	0.246 ± 0.018	19.0 ± 1.9	5.0 ± 0.5

[1]Counterion: K^+ ions.
[2]Electrolyte of 1M KCl.
[3]Number of water molecules per carboxylate group, calculated from electrolyte uptake and IEC.

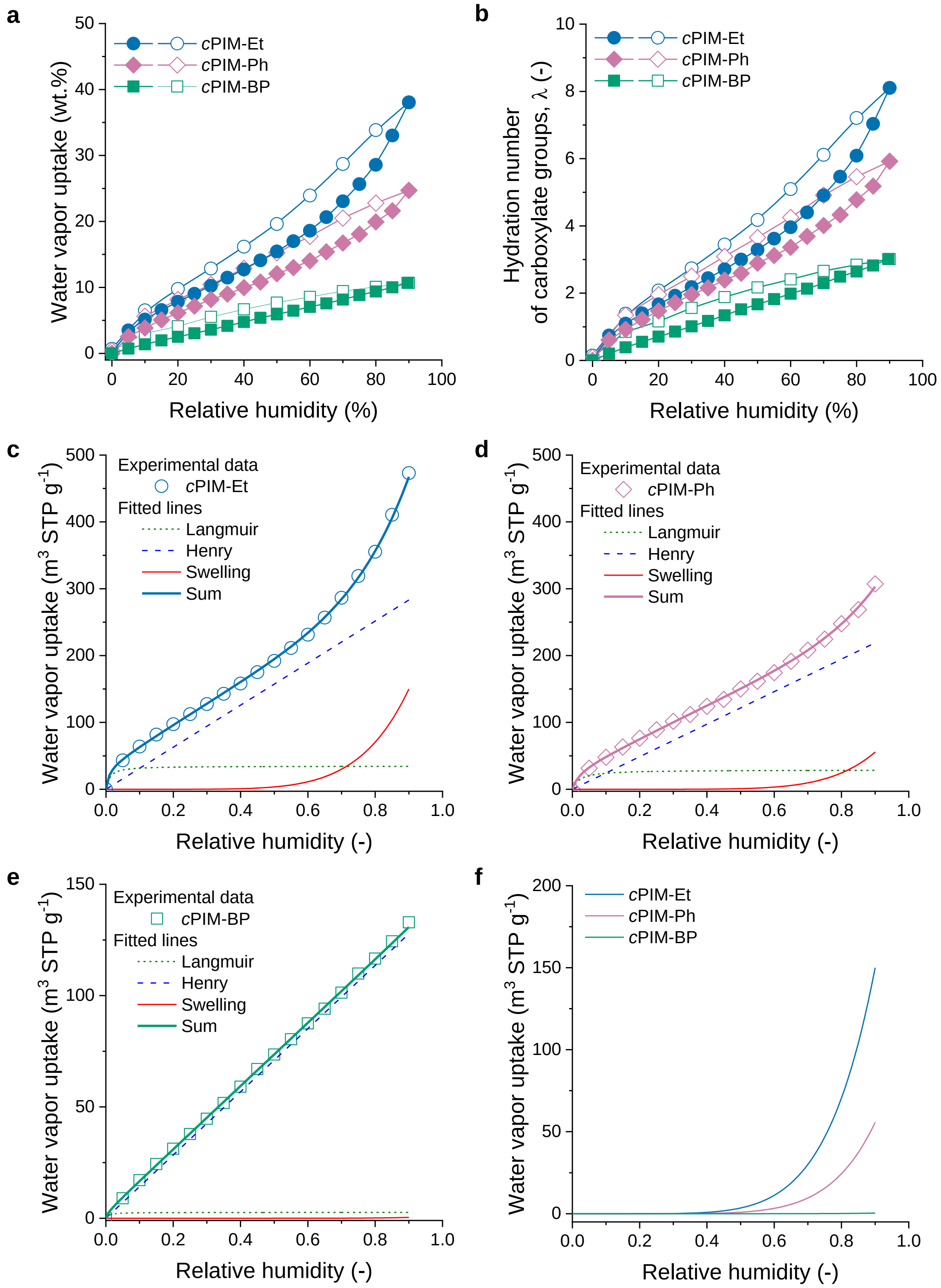

Supplementary Fig. 6 | Water vapor uptake at 25 ºC. a, Sorption isotherms based on weight percentage. **b**, Sorption isotherms based on the number of water molecules per carboxylate group. Analysis of sorption isotherm based on Langmuir, Henry and swelling mechanisms[73] for (**c**) *c*PIM-Et, (**d**) *c*PIM-Ph and (**e**) *c*PIM-BP. **f**, Comparison of fractional water sorption due to polymer swelling. The degree of swelling decreases with increasing hydrophobicity of polymer pendant groups.

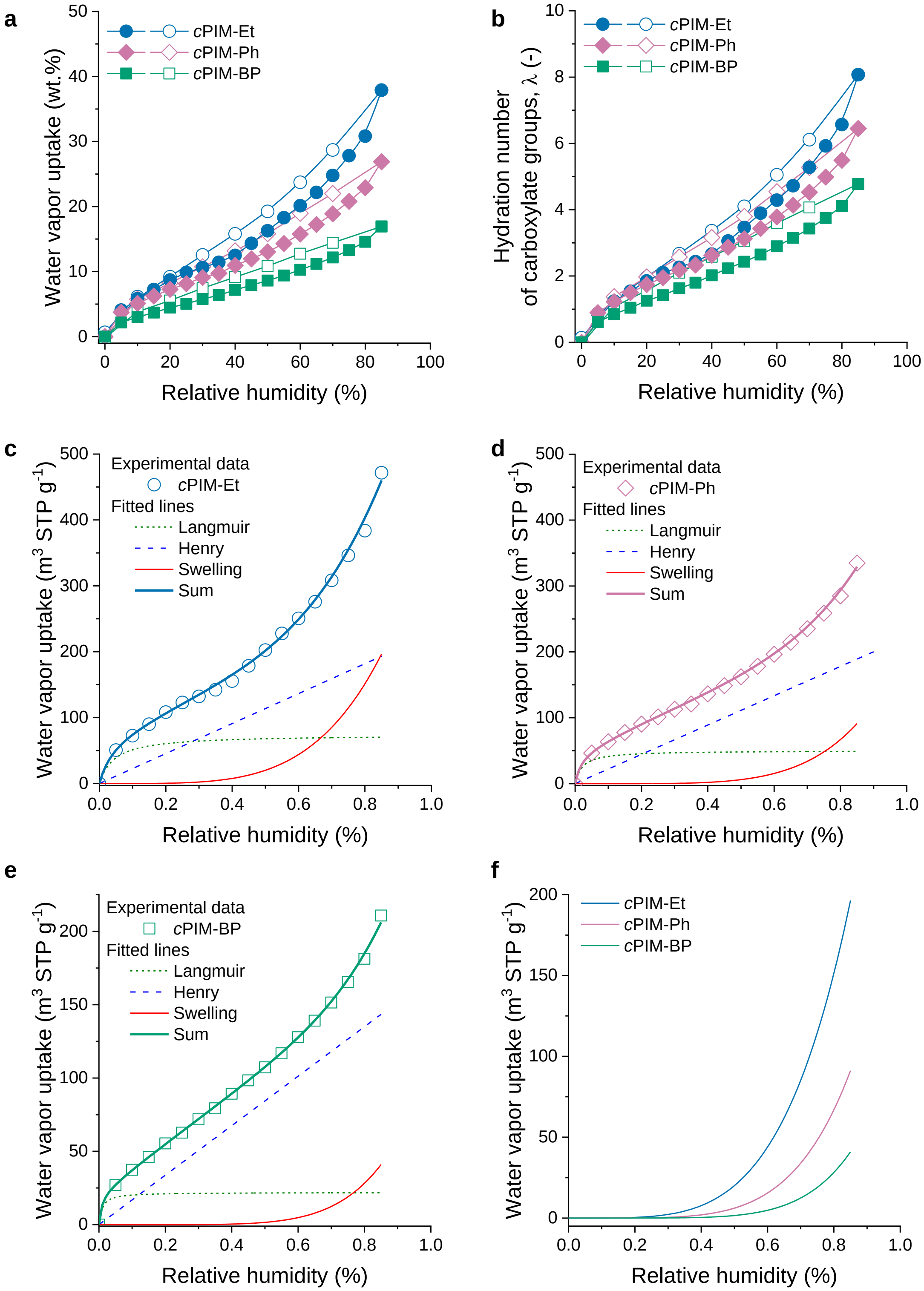

Supplementary Fig. 7 | Water vapor uptake at 40 ºC. a, Sorption isotherms based on weight percentage. **b**, Sorption isotherms based on the number of water molecules per carboxylate group. Analysis of sorption isotherm based on Langmuir, Henry and swelling mechanisms[73] for (**c**) *c*PIM-Et, (**d**) *c*PIM-Ph and (**e**) *c*PIM-BP. **f**, Comparison of fractional water sorption due to polymer swelling. The degree of swelling decreases with increasing hydrophobicity of polymer pendant groups.

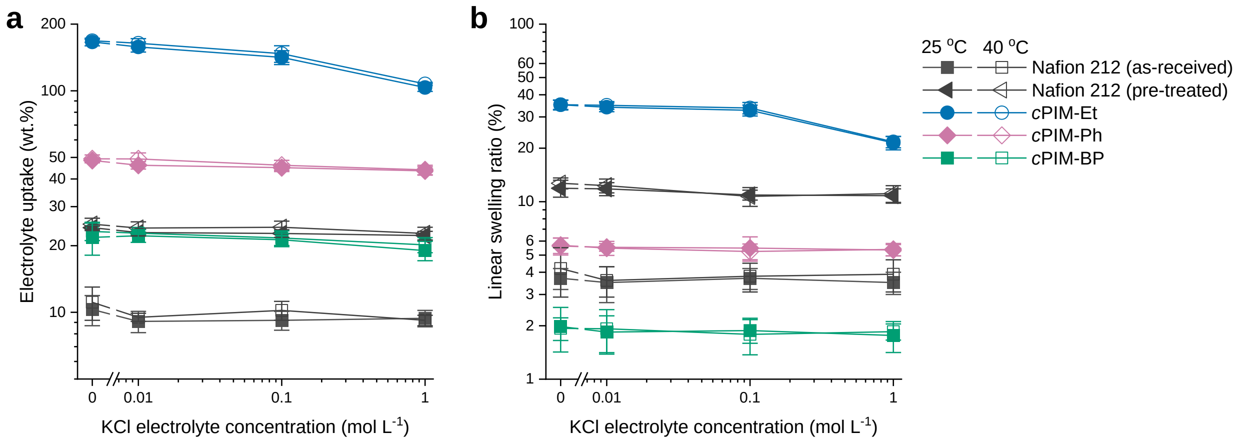

Supplementary Fig. 8 | Membrane swelling ratio. a, Gravimetric swelling ratio and **b**, linear swelling ratio. Measurements were conducted at 25 °C (solid symbols) and 40 °C (open symbols). Error bars represent the standard deviation of 3-4 independent samples.

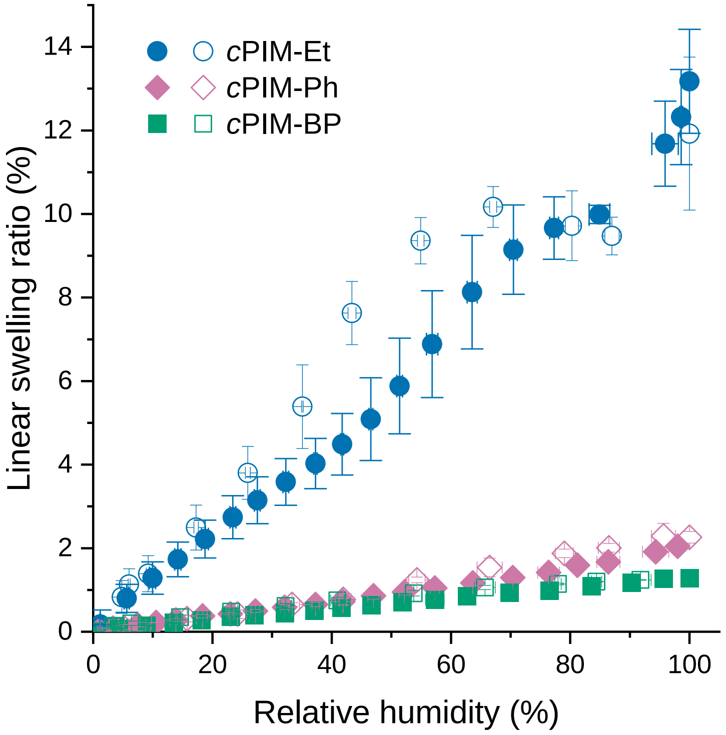

Supplementary Fig. 9 | Dimensional swelling ratio at various relative humidities measured by ellipsometry. Solid symbols represent swelling measured during adsorption and open symbols during desorption. Error bars represent the standard deviation of two independent samples which were measured twice.

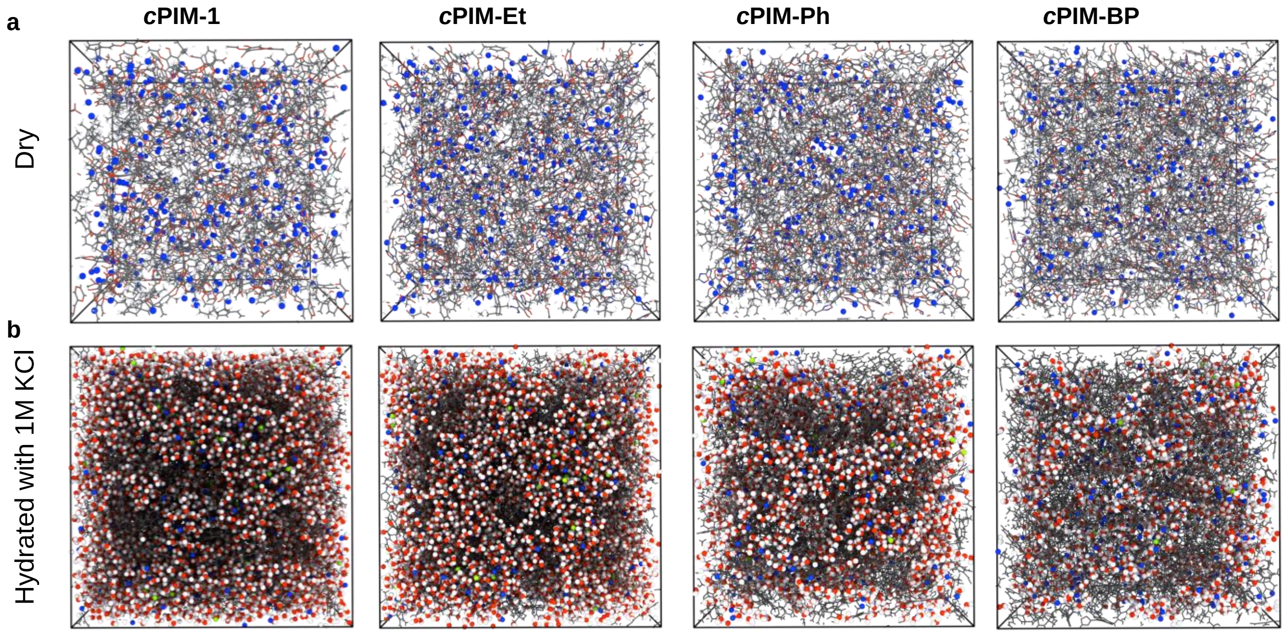

Supplementary Fig. 10 | Final frames of chain packing simulations. **a**, Dry polymers, **b**, polymers at experimentally comparable hydration levels. Carbon, oxygen, nitrogen, hydrogen, potassium ions and chloride ions are represented by grey, red, navy, white, blue and lime green, respectively.

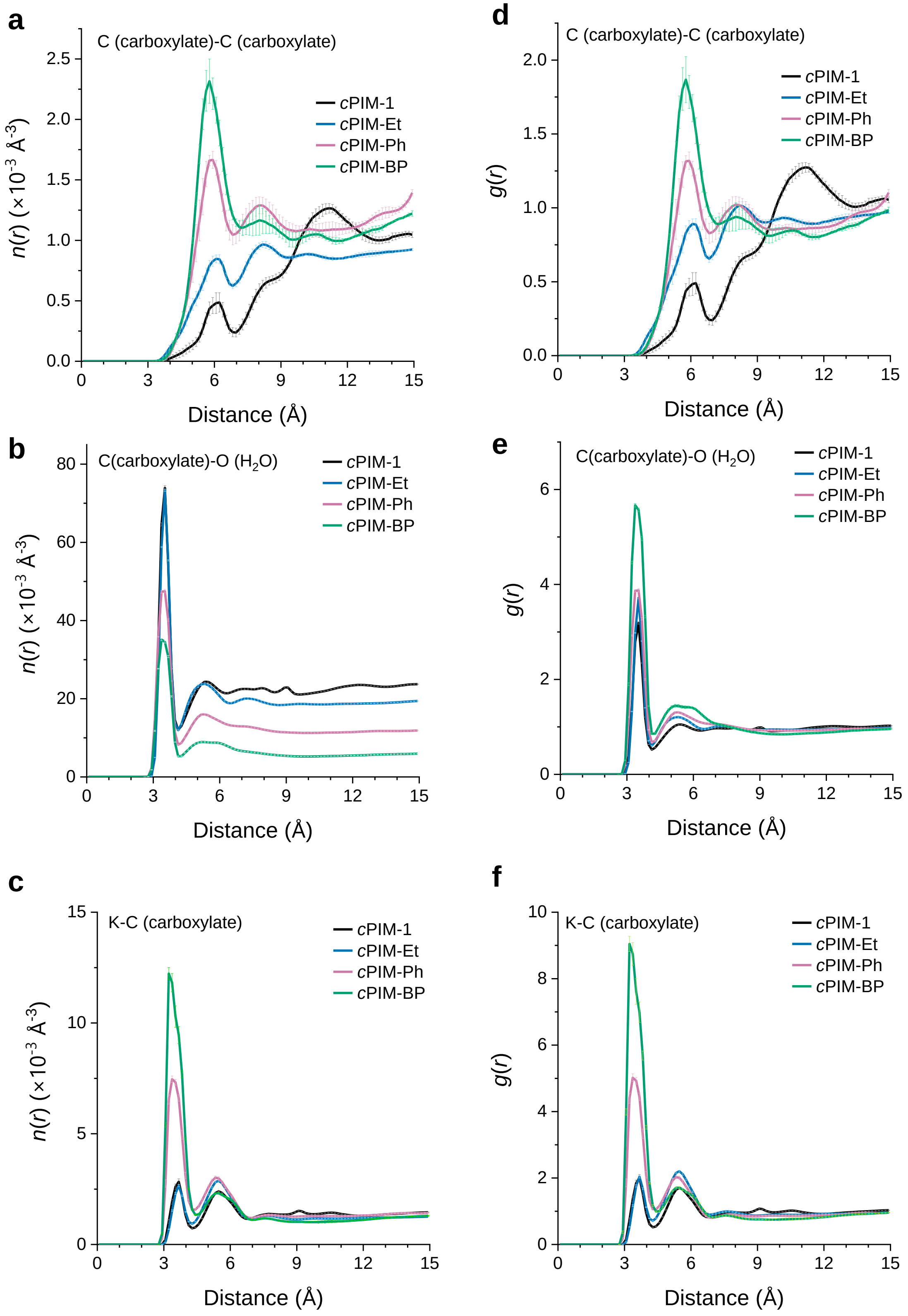

Supplementary Fig. 11 | Radial distribution functions (RDFs) derived from polymer models. a-c, Radial number density distribution function. **d-f**, Radial distribution function. The distance is relative to the carbon atom in carboxylate groups. Error bars represent standard deviation based on 5 independent polymer models.

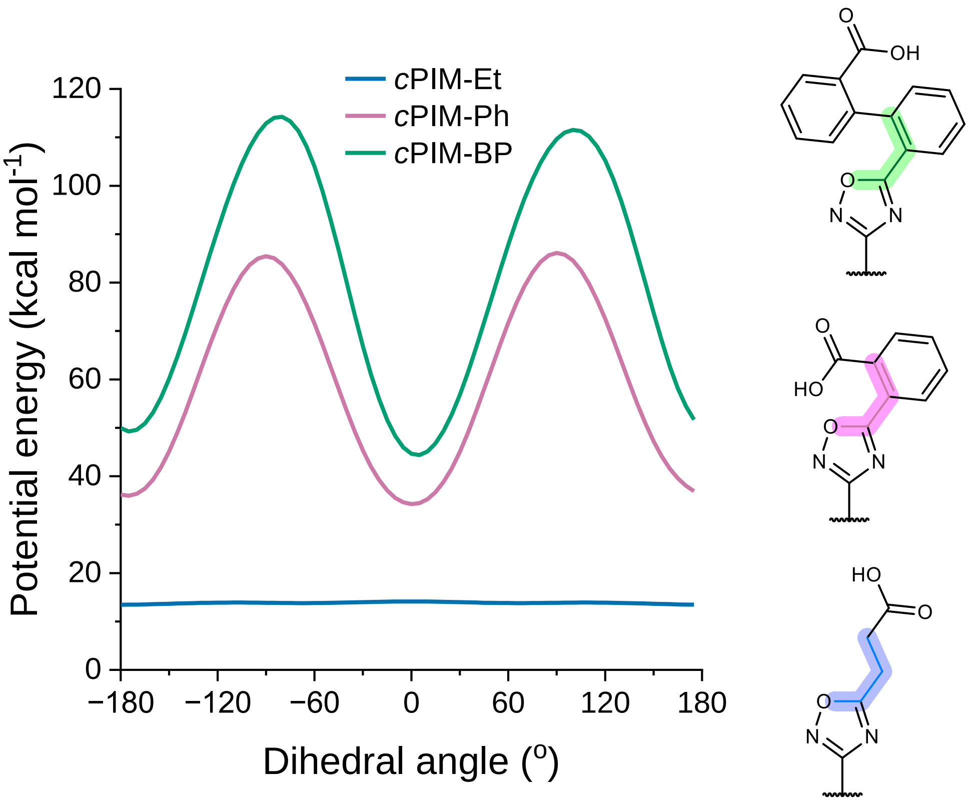

Supplementary Fig. 12 | Analysis of pendant group rigidity. Plots showing the increase in energy associated with the deviation in the marked dihedral angle within pendant groups of *c*PIM-Et, *c*PIM-Ph and *c*PIM-BP. The much lower potential energy for *c*PIM-Et suggests its high flexibility.

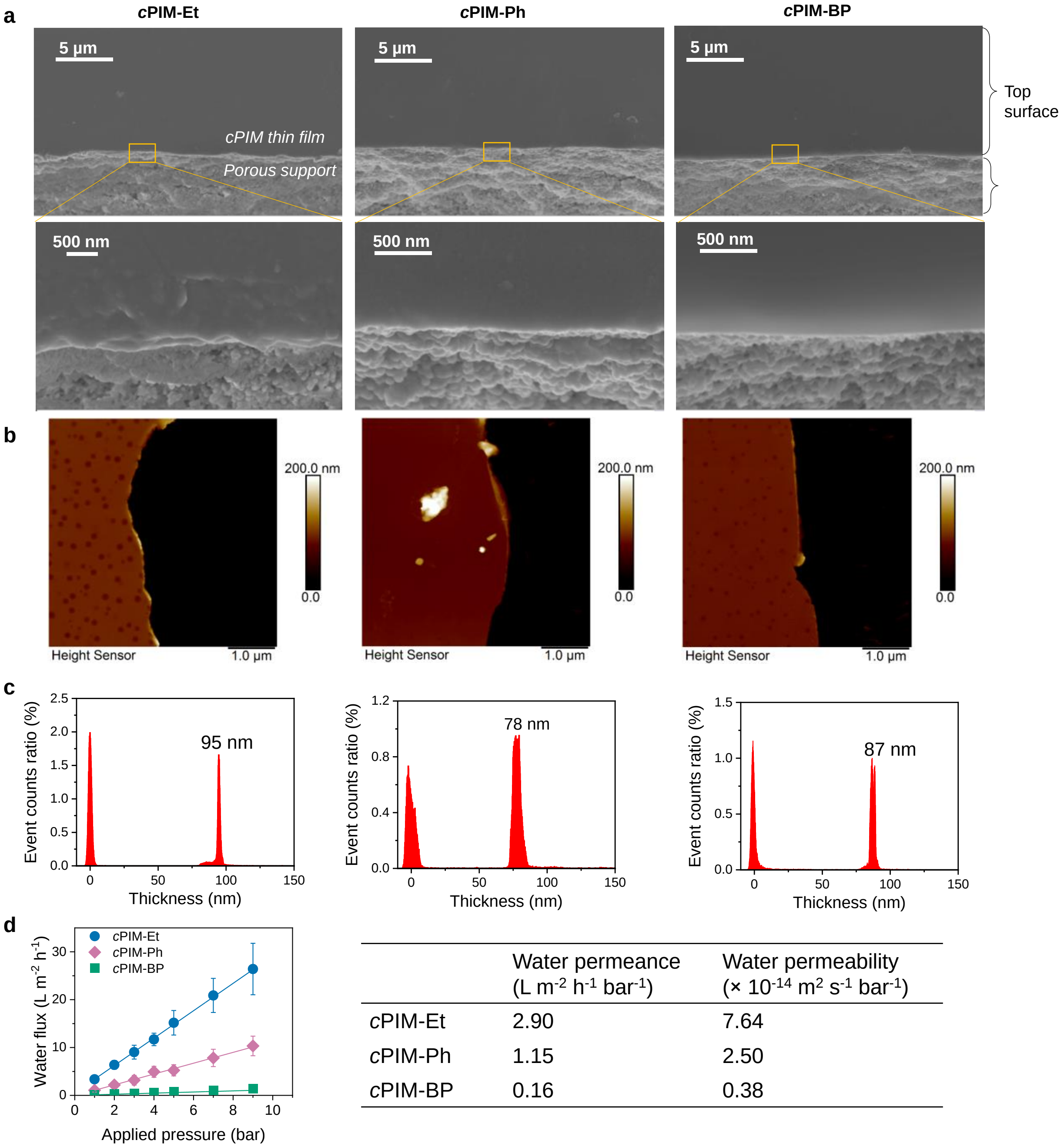

	Water permeance (L m^{-2} h^{-1} bar^{-1})	Water permeability (× 10^{-14} m^2 s^{-1} bar^{-1})
*c*PIM-Et	2.90	7.64
*c*PIM-Ph	1.15	2.50
*c*PIM-BP	0.16	0.38

Supplementary Fig. 13 | Pressure-driven water permeation. **a**, Cross-sectional SEM images of thin film composite (TFC) membranes comprising *c*PIM thin films coated on a porous polyacrylonitrile ultrafiltration membrane. **b**, AFM images and **c**, height profile of *c*PIM TFC membranes for characterization of the thickness of *c*PIM layer. **d**, Pure water flux as a function of applied cross-membrane pressure. A summary of water permeance and thickness-normalized permeability is provided in the figure.

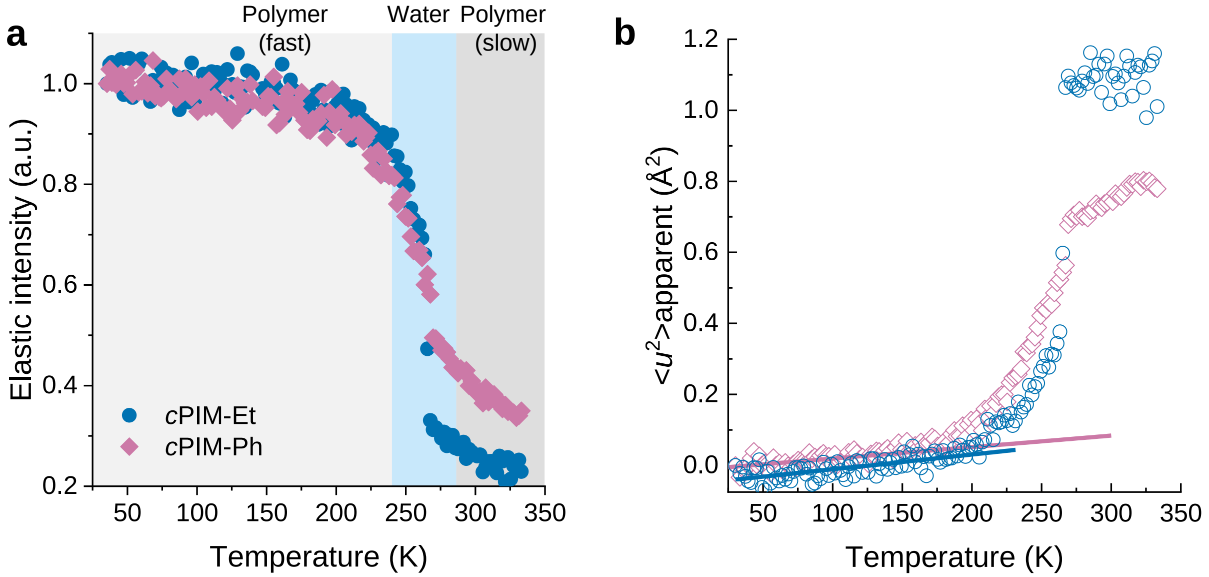

Supplementary Fig. 14 | Elastic fixed window scan (EFWS). **a**, Temperature-dependent elastic intensity. **b**, Apparent MSD ($<u^2>_{apparent}$) extracted from EFWS spectra in (**a**) as a function of temperature. MSD slopes extrapolated at $T < 100$ K are shown as lines. This region was selected for linear fitting since vibration becomes anharmonic at higher *T*. EFWS were acquired on BASIS at E_{res} = 3.5 μeV and Q = 0.9 Å with H_2O hydrated membrane samples.

The reduction in elastic intensity correlates with the number of protons becoming mobile within the investigated spectroscopic window. EFWS analysis indicates 3 processes:

(1) Below ~230 K, fast polymer dynamics (e.g., rotation of methyl groups) enter the spectroscopic window. MSD slope values are (0.28 ± 0.08) and (0.31 ± 0.07) × 10^{-3} Å^2 K^{-1} for *c*PIM-Et and *c*PIM-Ph, respectively. This minor difference is likely due to their different water content. Fast polymer dynamics at low *T* has a relaxation time in the tens of ps (~25 and ~6 ps at 200 and 333 K, respectively) with an activation energy of ~5.7 and ~6.0 kJ mol^{-1} for *c*PIM-Et and *c*PIM-Ph, respectively, suggesting no statistically relevant difference.

(2) From ~230 to ~280 K, a rapid reduction in elastic intensity is associated with confined water molecules becoming mobile, similar to the melting process of ice, in the investigated spectroscopic window. This transition appears more sharp for *c*PIM-Et than for *c*PIM-Ph, qualitatively indicating more bulk-like behavior in the former.

(3) Above 260 K, slow polymer dynamics become visible, leading to a plateau at high *T*. This slow process has a relaxation time in the range of a few hundred ps (~180 and ~100 ps at 265 and 333 K, respectively) with an activation energy of ~6.1 and ~4.2 kJ mol^{-1} for *c*PIM-Et and *c*PIM-Ph, respectively.

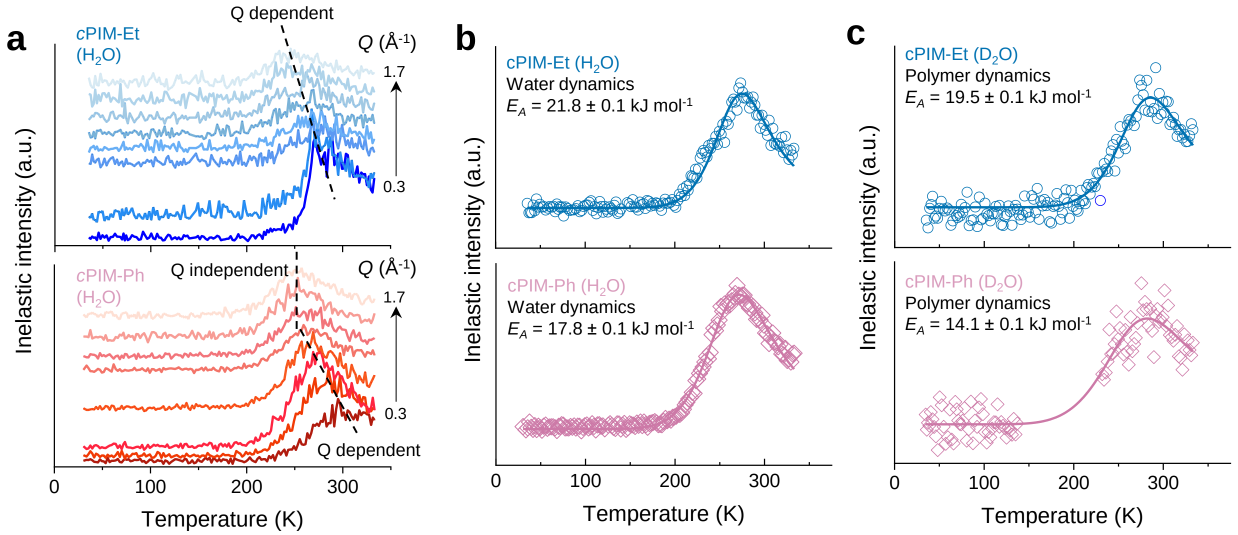

Supplementary Fig. 15 | Inelastic fixed window scan (IFWS). **a**, Temperature-dependent inelastic intensity of H_2O-hydrated membranes at varied Q (0.3-1.7 $Å^{-1}$ with a step of 0.2 $Å^{-1}$; ΔE = 8 μeV). **b**, Inelastic intensity of H_2O-hydrated membranes at Q = 1.1 $Å^{-1}$. Activation energy of water dynamics derived from the profile is labeled in the figure. **c**, Inelastic intensity of D_2O-hydrated membranes at Q = 1.1 $Å^{-1}$. Activation energy corresponding to polymer dynamics is labeled. Error bars of activation energy represent fitting error.

For *c*PIM-Et, a clear *Q*-dependence in IFWS indicates the translational motion of mobile water molecules. For *c*PIM-Ph, IFWS appears *Q*-independent at $Q > 1.1$ $Å^{-1}$, indicating nanoconfined water molecules. This difference is also supported by the activation energy of *c*PIM-Et (21.8 kJ mol^{-1}) comparable to that of supercooled bulk water[74], while that of *c*PIM-Ph is 17.8 kJ mol^{-1}.

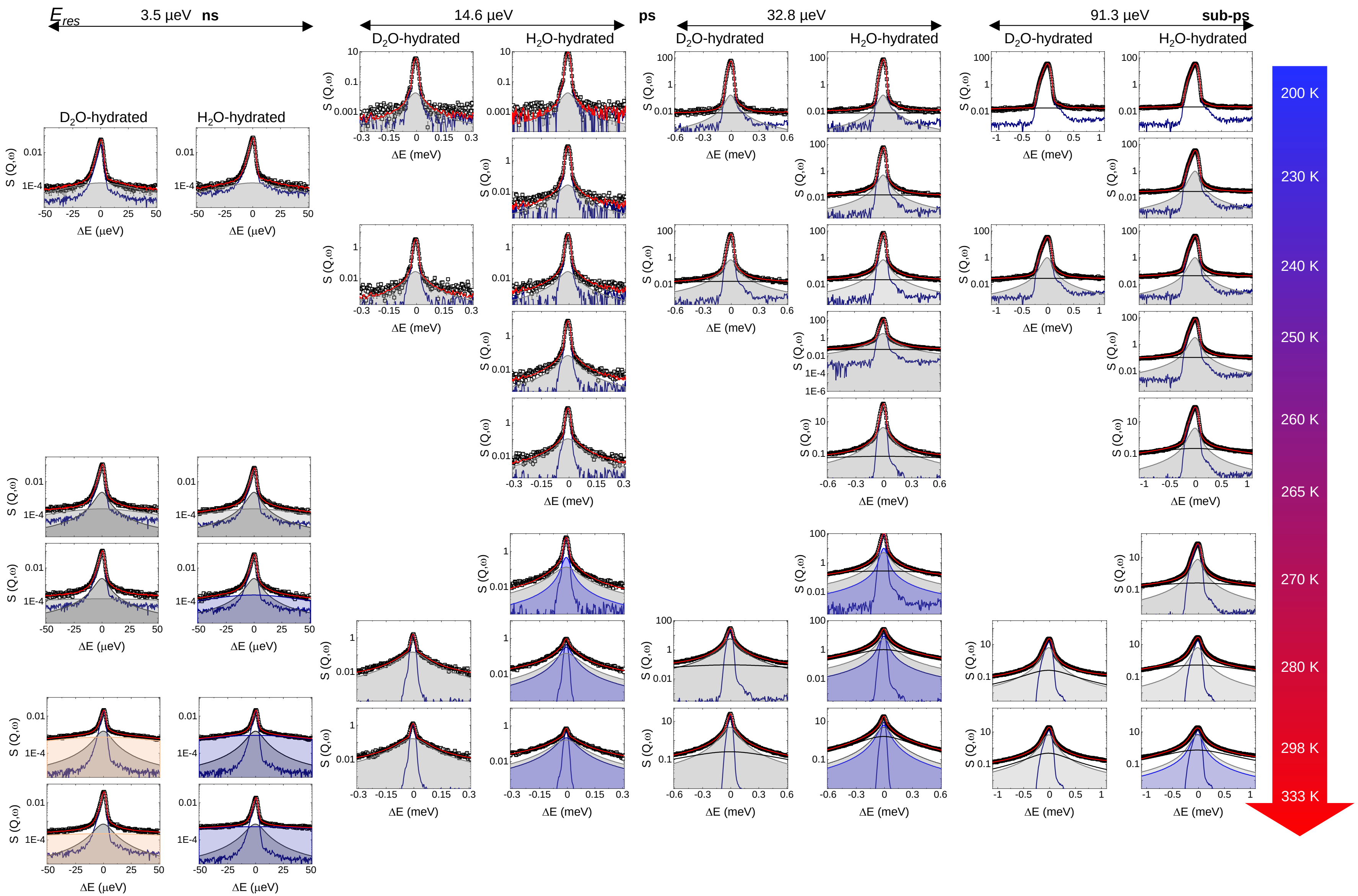

Supplementary Fig. 16 | Analysis of quasi-elastic neutron scattering (QENS) for *c*PIM-Et. Energy resolution, temperature and isotopic contrast are labeled at the top row and the left column. The instrument resolution function is shown as a solid blue line underneath the data points (open square) with the global fit in red lines. The polymer relaxation gives rise to a broad Lorentzian contribution (grey-shaded area) underlying the data. Water dynamics gives rise to the main Lorentzian contribution with a narrower line width (light blue shaded area); D_2O dynamics is represented as light orange shaded area. Solid black lines are associated with extremely fast contribution (e.g., polymer methyl rotation).

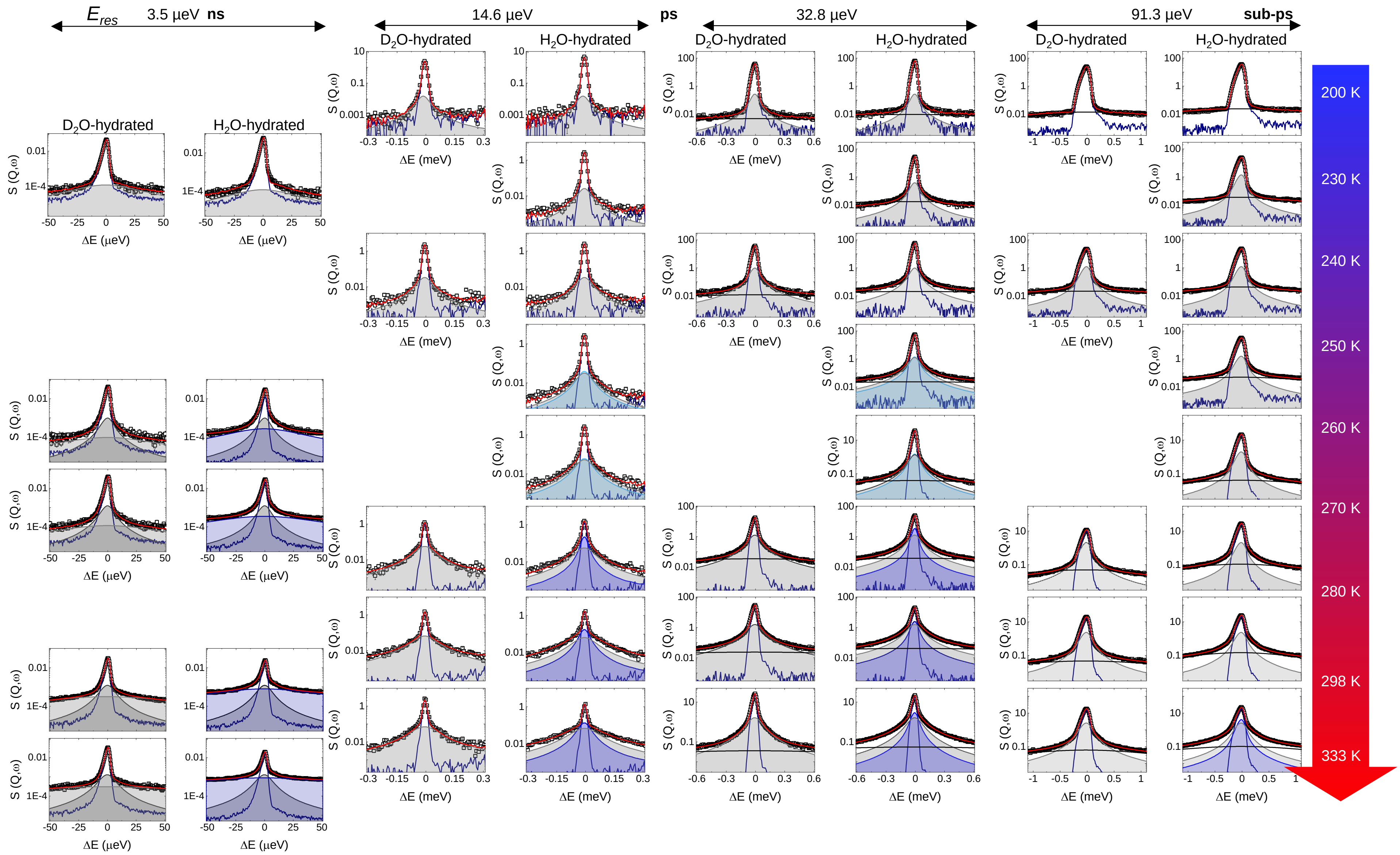

Supplementary Fig. 17 | Analysis of quasi-elastic neutron scattering (QENS) for *c*PIM-Ph. Energy resolution, temperature and isotopic contrast are labeled at the top row and the left column. The instrument resolution function is shown as a solid blue line underneath the data points (open square) with the global fit in red lines. The polymer relaxation gives rise to a broad Lorentzian contribution (grey-shaded area) underlying the data. The water dynamics give rise to the main Lorentzian contribution with a narrower line width (light blue shaded area). Solid black lines are associated with extremely fast contribution (e.g., polymer methyl rotation).

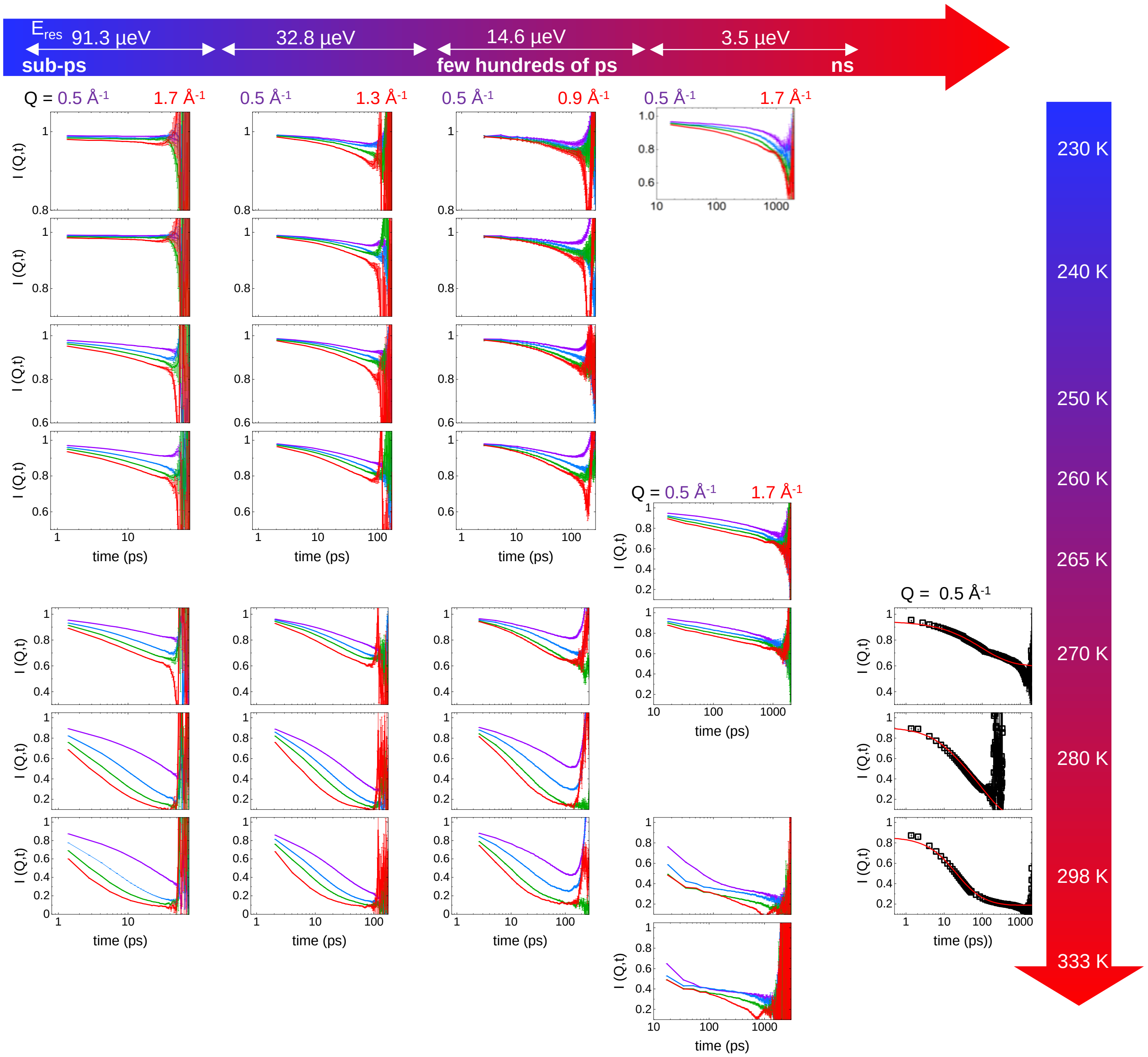

Supplementary Fig. 18 | Scattering profiles in the time domain for *c*PIM-Et. *Q*-dependency of *I*(*Q*,*t*) over the investigated timescales. The global fit (red continuous curve) in the figures next to the temperature bar is overlaid on the data points (black open squares).

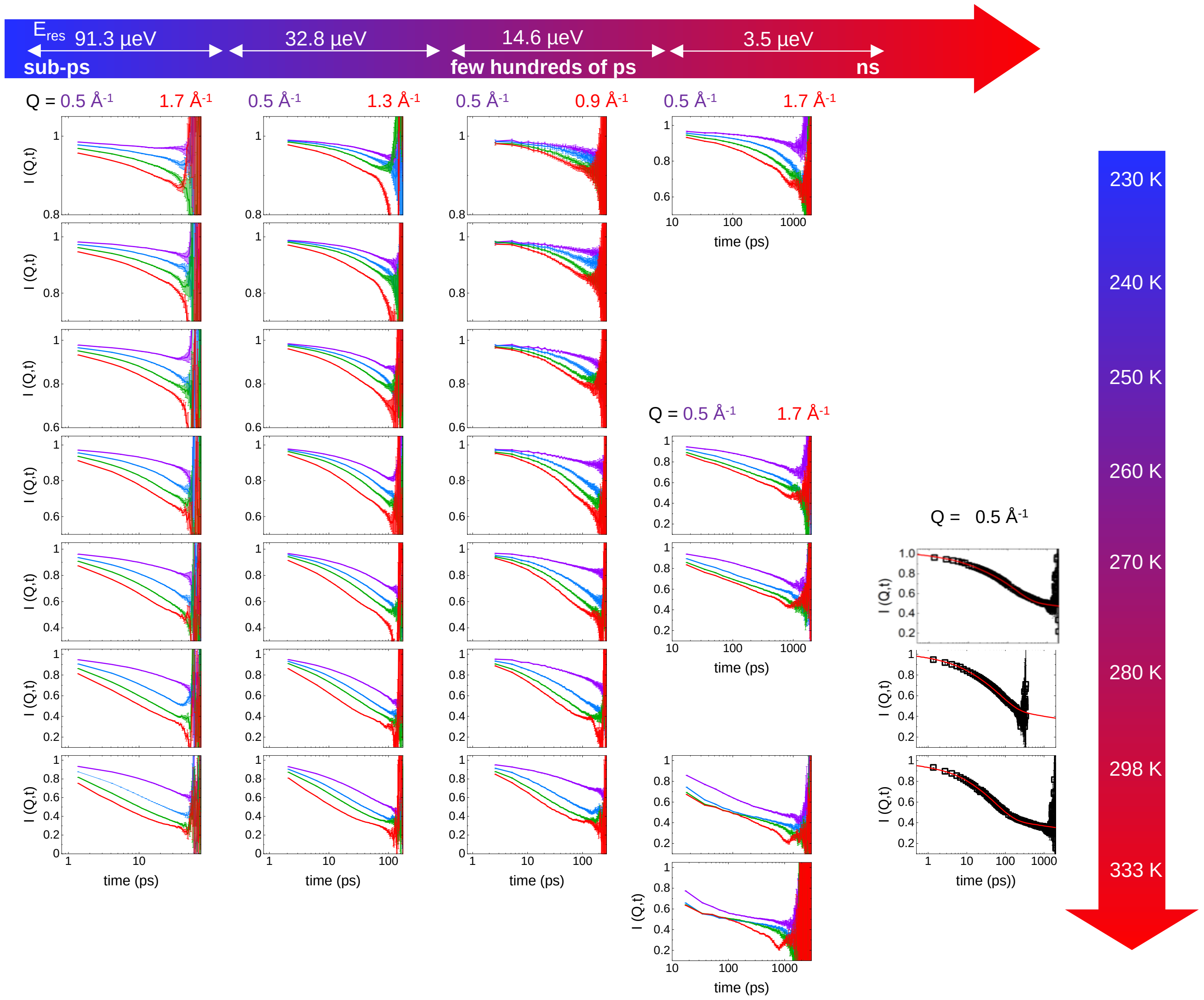

Supplementary Fig. 19 | Scattering profiles in the time domain for *c*PIM-Ph. *Q*-dependency of *I*(*Q*,*t*) over the investigated timescales. The global fit (red continuous curve) in the figures next to the temperature bar is overlaid on the data points (black open squares).

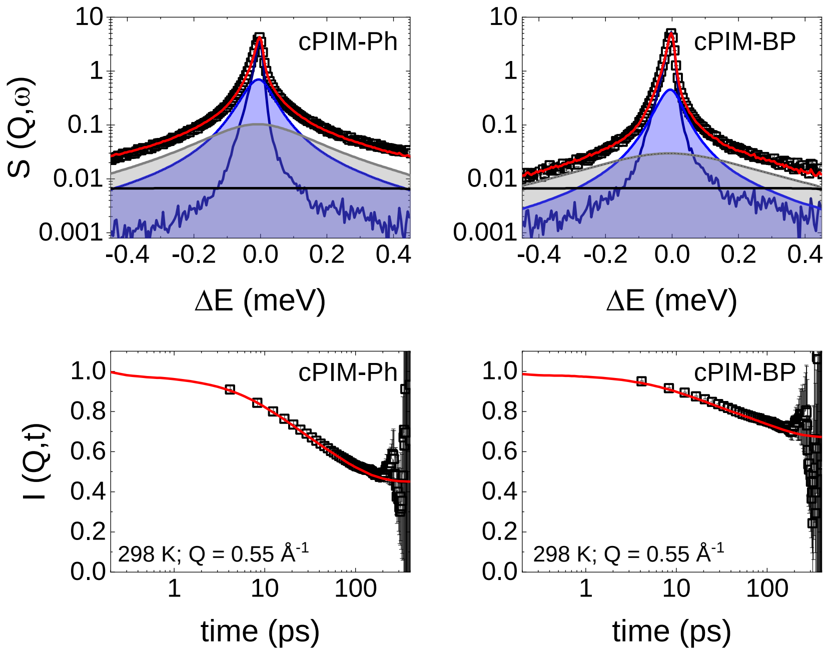

Supplementary Fig. 20 | Analysis of quasi-elastic neutron scattering (QENS). H_2O hydrated samples at an E_{res} of 17.5 μeV and temperature of 298 K. The instrument resolution function is shown as a solid blue line underneath the data points (open square) with the global fit in red lines. The polymer relaxation gives rise to a broad Lorentzian contribution (grey-shaded area) underlying the data. The water dynamics give rise to the main Lorentzian contribution with a narrower line width (light blue shaded area). Solid black lines are associated with extremely fast contribution (e.g., polymer methyl rotation). For scattering profiles in the time domain, the global fit (red continuous curve) in the figures next to the temperature bar is overlaid on the data points (black open squares).

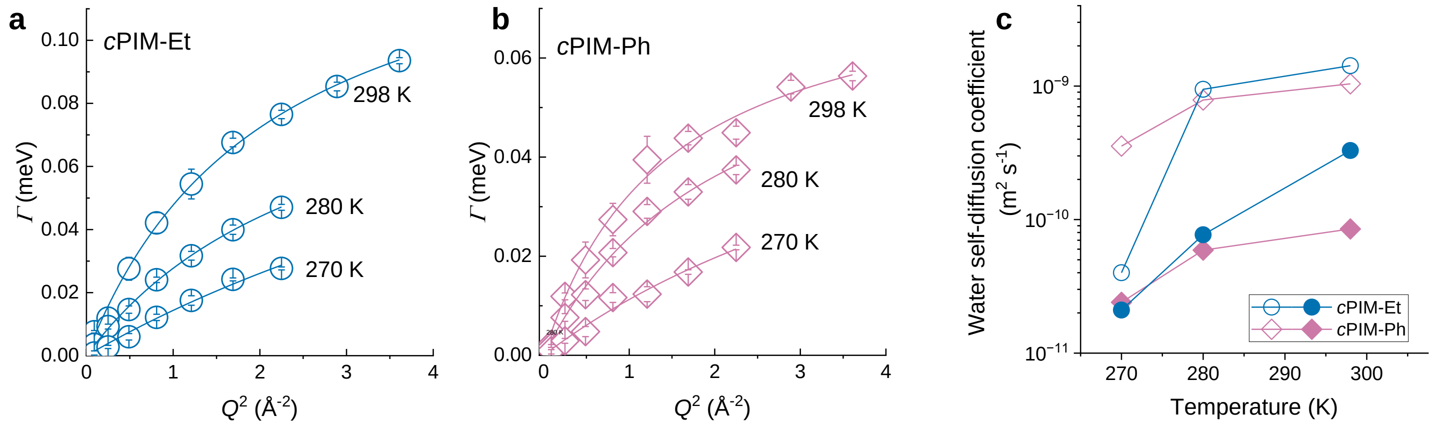

Supplementary Fig. 21 | Water dynamics derived from QENS based on the Gaussian model. Half width at half maximum (HWHM), $\Gamma(Q^2)$, of water translational dynamics as a function of Q^2 for (**a**) *c*PIM-Et and (**b**) *c*PIM-Ph membranes, both hydrated with H_2O. HWHM was derived from the main Lorentzian profile in Fig. S14 and S15 recorded at 14.6 and 32.8 μeV resolution and at selected temperatures 270, 280 and 298 K. The error bars correspond to the fitting uncertainties of the linewidth. **c**, Localized and long-range water self-diffusion coefficient calculated from (**a**) and (**b**) based on the Gaussian model. Open symbol: localized; solid symbol: long-range.

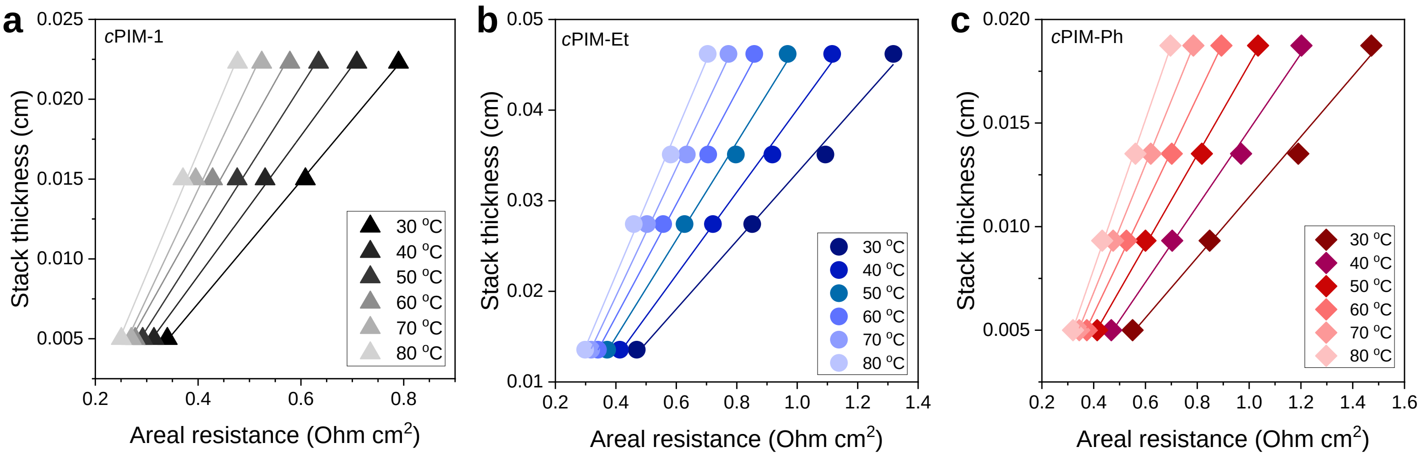

d

Temperature (°C)	*c*PIM-1		*c*PIM-Et		*c*PIM-Ph	
	Slope (S cm^{-1})	R^2	Slope (S cm^{-1})	R^2	Slope (S cm^{-1})	R^2
30	0.0384	1.00	0.0376	0.993	0.0146	0.994
40	0.0439	0.999	0.0457	0.996	0.0184	0.995
50	0.0506	0.998	0.0538	0.997	0.0218	0.998
60	0.0575	0.992	0.0621	0.997	0.0262	0.999
70	0.0681	0.991	0.0707	0.997	0.0309	0.999
80	0.0766	0.997	0.0790	0.996	0.0361	0.999

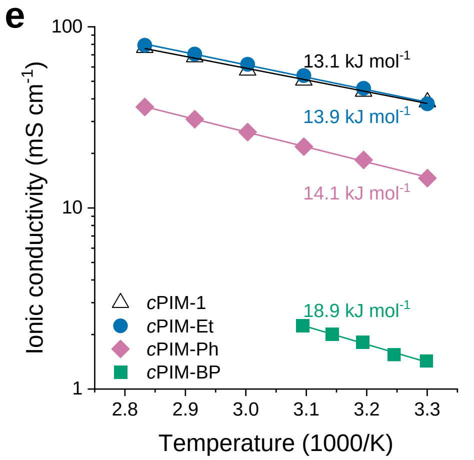

Supplementary Fig. 22 | Ionic conductivity of *c*PIMs. a-c, membrane stack thickness versus total areal resistance. **d**, Table summarizing ionic conductivity derived from the slope of the linear fitting in **a-c**. **e**, Ionic conductivity as a function of temperature.

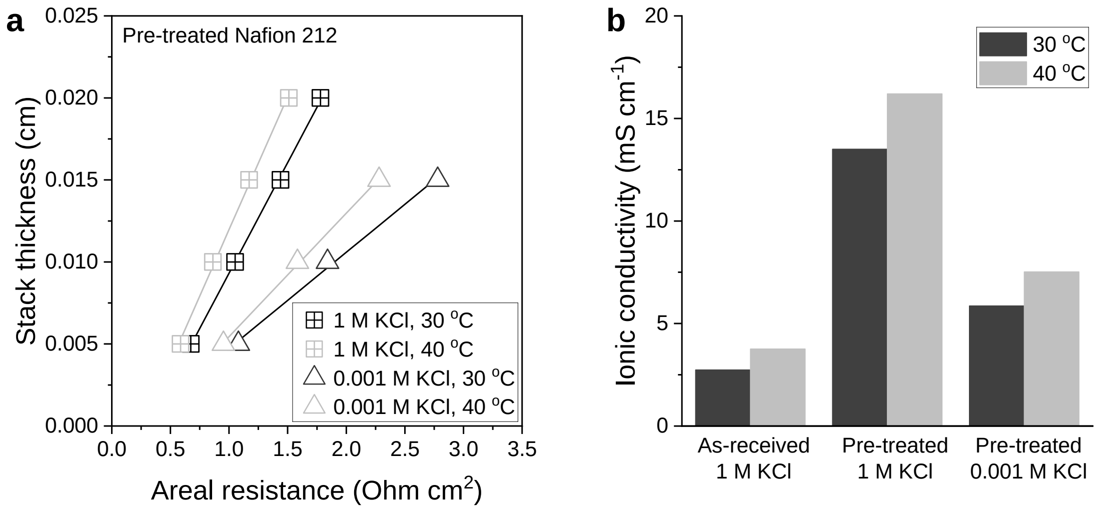

Supplementary Fig. 23 | Ionic conductivity of Nafion 212. a, membrane stack thickness versus total areal resistance. **b**, Ionic conductivity as a function of temperature.

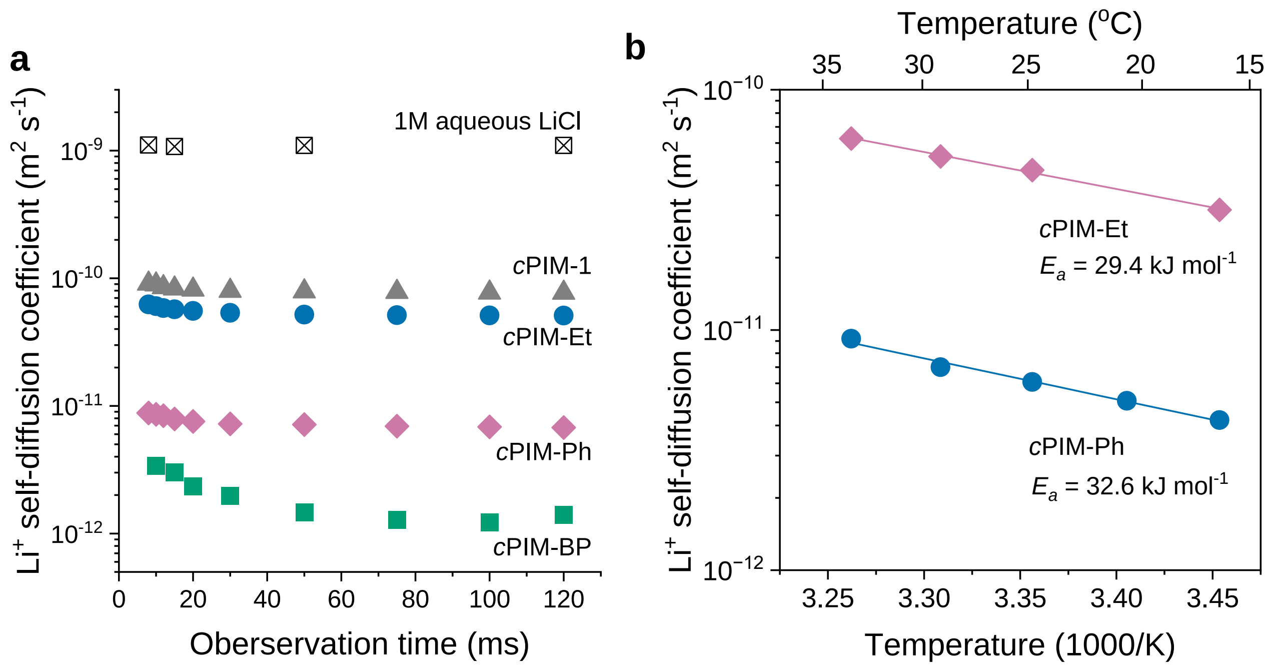

Supplementary Fig. 24 | Cation self-diffusion coefficient measured by ^{7}Li PFG-NMR. a, Li^+ self-diffusion coefficient as a function of observation time. **b**, Activation energy associated with Li^+ self-diffusion. Of note, Li^+ self-diffusion coefficients were measured instead of K^+ due to the relatively low receptivity, gyromagnetic ratio or sensitivity of ^{39}K, ^{40}K and ^{41}K.

Supplementary Table 2 | Apparent K^+ transference number. 4 independent membrane samples were measured and the standard deviation (not shown in the table) was in the range of 0.001 - 0.01.

KCl Concentration gradient (M | M)	Nafion 212 (Pristine)	Nafion 212 (Pre-treated)	*c*PIM-1	*c*PIM-Et	*c*PIM-Ph	*c*PIM-BP
0.01 | 0.1	0.97	0.91	0.82	0.83	0.93	0.98
0.1 | 1	0.98	0.84	0.82	0.82	0.92	0.97
0.3 | 3	0.96	0.73	0.56	0.57	0.75	0.89

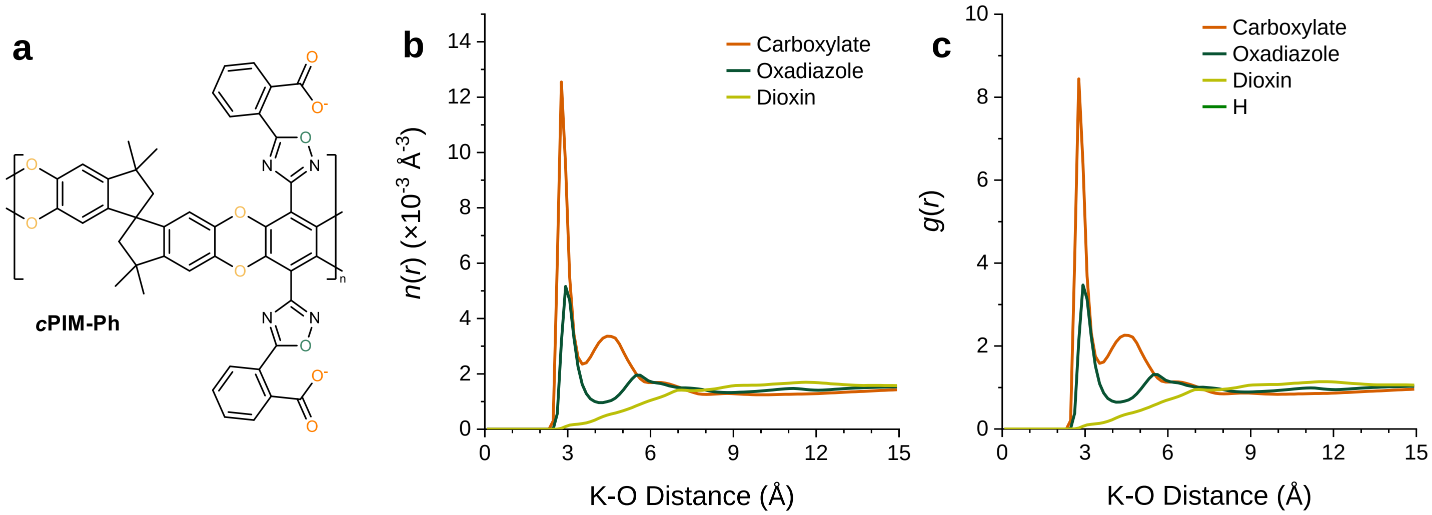

Supplementary Fig. 25 | Interactions between *c*PIM-Ph structural components with potassium ions. a, Chemical structure of *c*PIM-Ph with oxygen atom colored differently in each component. **b**, Radial number density distribution function. **c**, Radial distribution function. The calculation was based on the simulation box of *c*PIM-Ph polymer in the hydrated state.

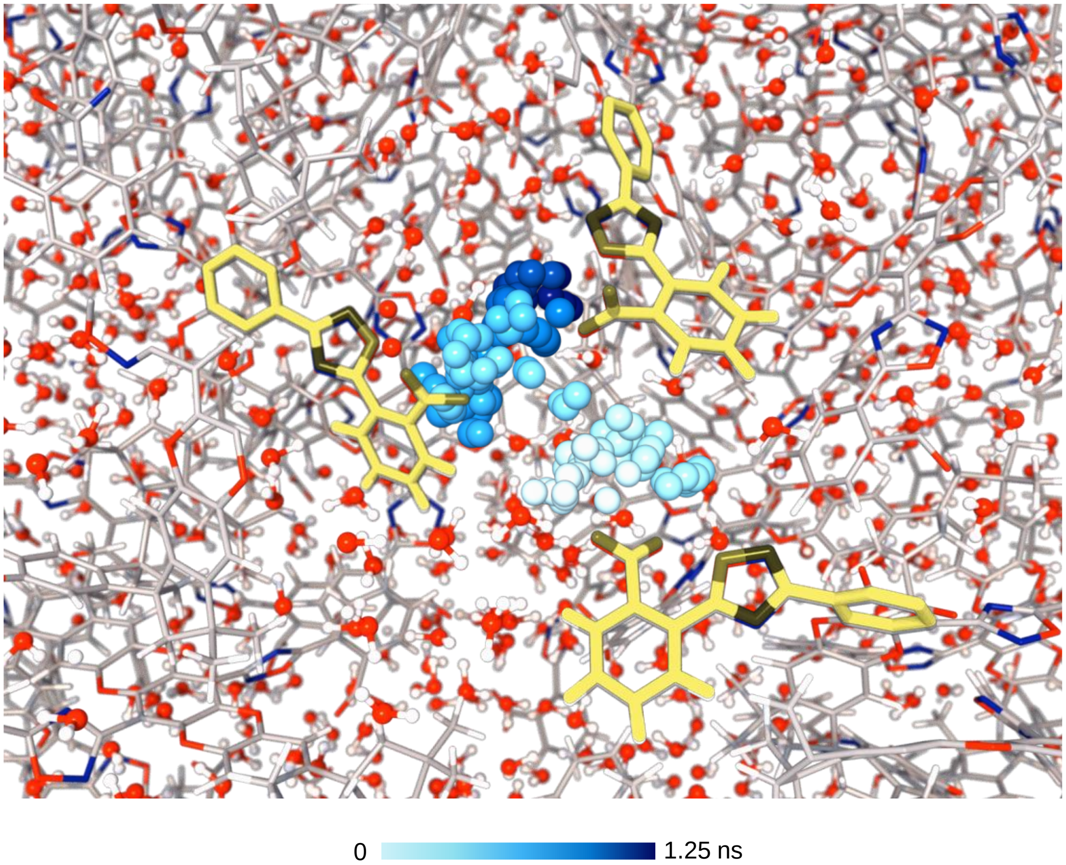

Supplementary Fig. 26 | Potassium ion trajectory in *c*PIM-Ph. The trajectory was derived from MD simulation over 1.25 ns at 300 K. The potassium ion is color-coded according to elapsed time. Pendant groups coordinating with the selected potassium ion across the observation time are highlighted in yellow.

Supplementary Table 3 | Properties and permeability of redox-active molecules. Pretreated Nafion 212 and *c*PIM-Ph TFC membranes (1.1 µm) were used in permeability tests. The chemical structures and optimized geometry of redox-active molecules investigated in this study are shown above the table. Geometry optimization was performed using the DMol3 program in BIOVIA Materials Studio. Box sizes were calculated based on Bondi's vdW radii. Although these dimensions provide a size estimate of bare molecules, the effective size and transport rates of these molecules are also influenced by their hydration, shape and flexibility.

2,6-DHAQ

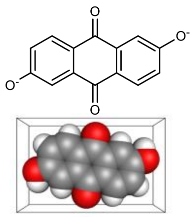

2,6-D2PEAQ

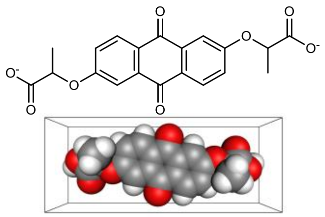

DHPS

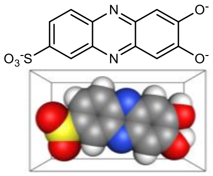

FMN

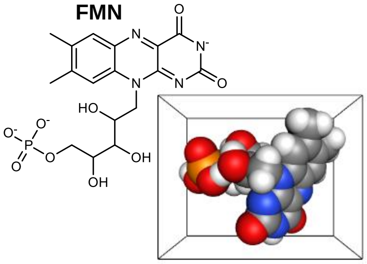

DHFL

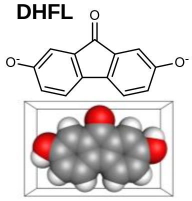

S-TEMPO

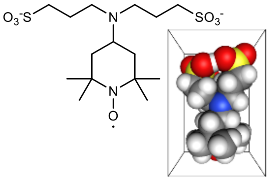

$(NPr)_2V$

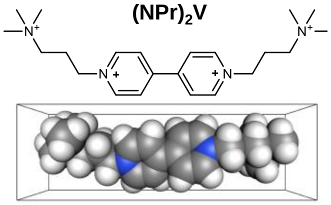

$(SPr)_2V$

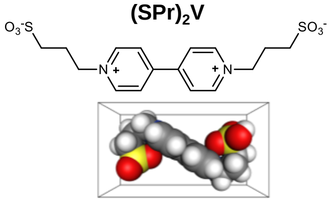

Redox-active molecules	Abbreviation	Molecular weight ($g\ mol^{-1}$)	Box size (Å)			Permeability ($cm^2\ s^{-1}$)		Ref.
			Width	Depth	Height	Nafion 212	*c*PIM-Ph	
Organometallic	$[Fe(CN)_6]^{4-}$	212	- (hydrated diameter 8.44)			1.8×10^{-8}	4.0×10^{-13}	15,18,32,57,75-80
Quinone	2,6-DHAQ	238	12.8	8.2	3.2	6.1×10^{-10}	2.0×10^{-11}	15,18,57,75,76
	2,6-D2PEAQ	382	20.3	8.1	5.4	3.6×10^{-10}	9.0×10^{-14}	32
Phenazine	DHPS	289	13.8	7.9	5.4	1.2×10^{-8}	9.3×10^{-13}	77
Alloxazine	FMN	453	15.0	12.0	7.5	7.4×10^{-10}	1.5×10^{-12}	78,79
Fluorenone	DHFL	210	12.6	8.0	3.2	1.8×10^{-8}	1.3×10^{-12}	80
Radical	S-TEMPO	414	13.9	9.2	8.4	1.5×10^{-9}	9.8×10^{-12}	81
Viologen	$(NPr)_2V$	356	23.0	7.4	6.4	5.6×10^{-9}	5.0×10^{-13}	82-84
	$(SPr)_2V$	400	16.4	9.1	6.6	2.9×10^{-8}	9.4×10^{-12}	85

Supplementary Table 4 | Summary of membrane ionic conductivity and selectivity

	Ionic conductivity<br>(mS cm^{-1}; temperature)	Ferrocyanide permeability<br>(cm^2 s^{-1}; temperature)	
***c*PIM-Et**	**38 (1M KCl; 30 ºC)**	**2.8×10^{-9} (pH = 7; 25 ºC)**	**This work**
***c*PIM-Ph**	**14 (1M KCl; 30 ºC)**	**4×10^{-13} (pH = 7; 25 ºC)**	
sCTF-BP	30 (DI water; 30 ºC)	9.4×10^{-11} (pH = 7; 25 ºC)	Zuo[18]
AO-PIM-1	2.8 (1M KCl; 30 ºC)	3.6×10^{-12} (pH = 9; 25 ºC)	Wang[14]
AO-PIM-$1_{80\%}$ |*c*PIM-$1_{20\%}$	7.3 (1M KCl; 30 ºC)	1.2×10^{-11} (pH = 9; 25 ºC)	
AO-PIM-$1_{60\%}$ |*c*PIM-$1_{40\%}$	13.4 (1M KCl; 30 ºC)	2.8×10^{-10} (pH = 9; 25 ºC)	
AO-PIM-$1_{40\%}$ |*c*PIM-$1_{60\%}$	22.7 (1M KCl; 30 ºC)	4.9×10^{-10} (pH = 9; 25 ºC)	
*s*PIM-SBF-0.53	2.7 (1M KCl; 30 ºC)	1.1×10^{-12} (pH = 9; 25 ºC)	Ye[17]
*s*PIM-SBF-0.98	11.3 (1M KCl; 30 ºC)	2.6×10^{-11} (pH = 9; 25 ºC)	
*s*PIM-SBF-1.40	19.5 (1M KCl; 30 ºC)	1.7×10^{-10} (pH = 9; 25 ºC)	
*s*PIM-SBF-1.67	31.2 (1M KCl; 30 ºC)	2.3×10^{-9} (pH = 9; 25 ºC)	
*s*PIM-SBF-1.86	39.4 (1M KCl; 30 ºC)	7.5×10^{-9} (pH = 9; 25 ºC)	
AO-PIM-DBMP	12.0 (1M KOH; 30 ºC)<br>3.5 (1M KCl; 30 ºC)	1.3×10^{-9} (pH = 14; 25 ºC)<br>4.8×10^{-12} (pH = 9; 25 ºC)	Ye[16]
AO-PIM-BTrip	15.0 (1M KOH; 30 ºC)<br>4.8 (1M KCl; 30 ºC)	6.2×10^{-10} (pH = 14; 25 ºC)<br>4.3×10^{-12} (pH = 9; 25 ºC)	
AO-PIM-SBF	13.8 (1M KOH; 30 ºC)<br>4.1 (1M KCl; 30 ºC)	3.5×10^{-10} (pH = 14; 25 ºC)<br>4.3×10^{-12} (pH = 9; 25 ºC)	
PIM-EA-TB	0.44 (1M NaOH; 30 ºC)	1.0×10^{-10} (pH = 14; 25 ºC)	Tan[15]
AO-PIM-1	4.8 (1M NaOH; 30 ºC)	1.68×10^{-9} (pH = 14; 25 ºC)	
AO-PIM-1	7.8 (1M KOH; not provided)	2.6×10^{-10} (pH = 14; not provided)	Baran[86]
SPX-BP-0.95	9.7 (1M KOH; 30 ºC)	7.7×10^{-11} (pH = 14; room temperature)	Zuo[87]
Porous PES/SPEEK	28.7 (0.5M NaCl; not provided)	1.5×10^{-8} (3M NaOH; not provided)[a]	Yuan[88]
	14.4 (0.5M NaCl; not provided)	1.6×10^{-9} (3M NaOH; not provided)[a]	
	36 (0.5M NaCl; not provided)	9.7×10^{-9} (3M NaOH; not provided)[a]	
	43 (0.5M NaCl; not provided)	1.1×10^{-8} (3M NaOH; not provided)[a]	
HGO graphene oxide	1.52 (2M KOH; not provided)<br>0.654 (2M KCl; not provided)	2×10^{-10} (not provided)	Zhang[89]
PBI	3.03 (3M NaOH; not provided)[b]	8.19×10^{-6} (3M NaOH; not provided)[c]	Zhang[90]
Porous PES/PEG	4.1 (3M KOH; not provided)	1.30×10^{-9} (3M NaOH; not provided)	Chen[91]
	5.3 (3M KOH; not provided)	5.56×10^{-9} (3M NaOH; not provided)	
	15.8 (3M KOH; not provided)	2.41×10^{-8} (3M NaOH; not provided)	
SPPO	12.3 (1M KOH; not provided)	2.5×10^{-10} (DI water; not provided)	Li[92]
Celgard 3501	8.66 (1M KOH; not provided)	2.2×10^{-7} (pH = 14; not provided)	Baran[86]
Nafion 212	8.3 (1M KOH; 30 ºC)<br>5.4 (1M KCl; 30 ºC)	2.8×10^{-8} (pH = 14; 25 ºC)<br>2.2×10^{-8} (pH = 9; 25 ºC)	Ye[16]
Nafion 212	11 (1M NaOH; 30 ºC)	1.79×10^{-8} (pH = 14; 25 ºC)	Tan[15]
Nafion 212	6.61 (room temperature)	4.5×10^{-9} (pH = 14; room temperature)	De Porcellinis[93]
Nafion 117	8.6 (1M KOH; 30 ºC)	4.6×10^{-8} (pH = 14; room temperature)	Zuo[87]
Nafion 115	11.9 (1M KCl; 30 ºC)	2.2×10^{-9} (pH = 14; 25 ºC)	Ye[17]
Fumatech E610	~1 (1M K^+; not provided)[d]	4.4×10^{-12} (pH = 9; not provided)	Kwabi[94]
Selemion CSO	3.9 (1M KCl; room temperature)[e]	4.4×10^{-9} (pH = 7; room temperature)[f]	Luo[95]

[a]Permeability of ferricyanide
[b]Conductivity values were calculated by the areal resistance (1.152 Ohm cm^2) and membrane thickness (35 µm)
[c]Permeability is provided in another work published by the same group [86].
[d]Conductivity values were estimated by the areal resistance (~ 1 Ohm cm^2) and membrane thickness (10 µm)
[e]Conductivity values were calculated by the areal resistance (2.57 Ohm cm^2) and membrane thickness (100 µm)
[f]Permeability was estimated based on membrane thickness (100 µm), active area (10 cm^2), electrolyte volume (12 mL), as well as the crossover percentage (14.49%) over a cycling time of 110 h.

Supplementary Table 5 | Analysis of ferrocyanide crossover after cycling tests

Membrane	Capacity decay rate[a] (% per day)	Ferrocyanide crossover rate in operating battery[b] (% per day)
*c*PIM-Ph	0.014	0.007
*s*PEEK	0.21	0.15
Nafion 212	1.36	1.2

[a]Capacity rate was derived from the cycling data.
[b]Ferrocyanide crossover rate was quantified based on the amount of iron in the post-cycling anolyte solution using ICP-MS.

Supplementary Table 6 | Summary of aqueous organic RFB performance.

Catholyte/anolyte[a]	Membrane	Temp. (°C)	pH	Electron conc.[b] (mol L^{-1})	Electron excess factor[c]	**Energy density[d] (Wh L^{-1})**	**Decay rate (% per day)**	Cycling duration (days)	Potentio-static hold	Ref.
2,6-D2PEAQ‖**$Fe(CN)_6^{3+/4+}$**	*c*PIM-Ph	40	7	2.2‖1.5	0.1	**24.5**	**0.014**	61	√	This work
	Nafion 212	40	7	2.2‖1.5	0.1	**24.5**	**1.36**	15	√	
	*s*PEEK	40	7	2.2‖1.5	0.1	**24.5**	**0.21**	42	√	
**2,6-D2PEAQ**‖$Fe(CN)_6^{3+/4+}$	Nafion 212	40	7	2.2‖1	1.57	**8.4**	**0.013**	28	√	32
		r.t.	7	2.2‖1	0.27	**13.6**	**0.02**	14	√	
**2-2PEAQ**‖$Fe(CN)_6^{3+/4+}$	Nafion 212	40		2‖0.3	4	**2.3**	**0.09**	10	√	96
**2,6-DBEAQ**‖$Fe(CN)_6^{3+/4+}$	Fumasep E620	-	12	1‖0.3	0.8	**2.6**	**0.04**	6	√	94
2,6-DPPAQ‖**$K_4Fe(CN)_6$**	Fumasep E620	-	9	1‖0.4	5.4	**1.6**	**0.014**	12	√	97
	Nafion 115	30	9	0.8‖1	1	**6.4**	**2.66**	5		17
	sPIM-SBF-1.40	30	9	0.8‖1	1	**6.9**	**0.127**	5		
	AO-PIM-1	30	9	0.1‖0.1	1	**1.3**	**0.13**	29		16
	AO-PIM-$1_{80\%}$ |*c*PIM-$1_{20\%}$	30	9	0.1‖0.1	1	**1.2**	**0.12**	8	√	14
**PEGAQ**‖$Fe(CN)_6^{3+/4+}$	Fumasep E620	-	7	3‖0.31	1.21	**3.4**	**0.5**	18		98
**DBAQ**‖$Fe(CN)_6^{3+/4+}$	Fumasep E620	20	12	1‖0.3	4.8	**1.44**	**0.0084**	16	√	99
**DPivOHAQ**‖$Fe(CN)_6^{3+/4+}$	Fumasep E620	20	12	1‖0.3	4.8	**1.5**	**0.014**	16	√	
		20	14	1‖0.3		**1.5**	**0.0018**	16	√	
**AQDP**‖$Fe(CN)_6^{3+/4+}$	Fumasep E620	-	12	1‖0.5	4	**1.94**	**0.025**	11	√	100
MV‖4-HO-TEMPO	Selemion AMV	-	7	0.5‖0.5	0	**6.0**	**27.5**	< 1		101
PolyMV‖PolyTEMPO	DialysisMem	-	7	0.37‖0.34	0	**8.0**	**7.5**	< 1		102
MV‖TEMPTMA	Fumasep FAA	-	7	2‖2	0	**27.4**	**0.27**	10		103
MV‖FcN	Selemion AMV	-	7	0.7‖0.7	0	**9.9**	**~1.7**	~11		104
BTMAP-Vi‖BTMAP-Fc	Selemion DSV	-	7	1.3‖1.3	0	**12**	**0.1**	14	√	84
$[(NPr)_2V]Cl_4$‖N^{Me}-TEMPO	Selemion AME	-	7	0.5‖0.5	0	**6.2**	**0.7**	~4		105
$[(Me)(NPr)V]Cl_3$‖FcN	Selemion AME	-	7	0.5‖0.5	0	**7.08**	**8.8**	< 1		82
$[(NPr)_2V]Br_4$‖FcN	Selemion AME	-	7	0.5‖0.5	0	**6.46**	**0.5**	~ 1.5		82
BTMAP-Vi‖**TMAP-TEMPO**	Selemion AMV	-	-	1.5‖1.5	0.5	**15**	**0.55**	7.2		83
**$(SPr)_2V$**‖KI	Selemion CSO	-	7	0.5‖2	3	**6.25**	**0.45**	~ 3		85
$(SPr)_2V$‖$(NH_4)_4Fe(CN)_6$	Selemion CSO	-	7	0.5‖0.5	0	**4.3**	**2.64**	4.6		95
**BPP-Vi**‖$K_4Fe(CN)_6$	Fumasep E620	-	9	1‖0.3	0.94	**3.2**	**0.016**	13	√	81
$(NPr)_2TTZ$‖N^{Me}-TEMPO	Selemion AMV	25	7	0.2‖0.2	0	**3.86**	**2.25**	~1.5		106

[a]Capacity-limiting species is marked out in bold. [b]Electron concentration refers to theoretical electron per litre of electrolyte for catholyte (left) and anolyte (right), respectively. [c]Electron excess factor = Total electron of non-capacity limiting side/Total electron of capacity limiting side – 1. [d]Demonstrated energy density = Demonstrated discharge capacity / total volume of catholyte and anolyte × cell voltage.

Supplementary Table 6 (*Continued*) | Summary of aqueous organic RFB performance.

Catholyte/anolyte[a]	Membrane	Temp. (°C)	pH	Electron conc.[b] (mol L^{-1})	Electron excess factor[c]	**Energy density[d] (Wh L^{-1})**	**Decay rate (% per day)**	Cycling duration (days)	Potentio-static hold	Ref.
Diquat 5||FcNCl	Selemion AMV	25	7	0.5||0.5	0	**4.5**	**0.8**	25		107
exDMeBP||FcNCl	Selemion DSV	-	7	1||0.25	0.5	**4.3**	**~4.5**	~1.8	√	108
1,6-DPAP||$Fe(CN)_6^{3+/4+}$	Nafion 117	r.t.	8	1||0.3	-	**-**	**0.0015**	12		109
BTMAP-Vi||**$Fe(Bhmbpy)_3$**	Selemion DSV	-	7	0.46||0.5	4	**~1.7**	**0.16**	7	√	110
SPr-Bpy|| **$Na_4[Fe^{II}(Dcbpy)_2(CN)_2]$**	Nafion 212	22	7	1.2||1.02	0.02	**6.2**	**2.3**	10.8		111
	Fumasep FKE-50	22	7	1.6||1	3	**12.5**	**0.25**	13.5	√	
**2,6-DHAQ**||$K_4Fe(CN)_6$	Nafion 212	20	14	1||0.4	0.5	**6.8**	**8**	1.3		57
2,6-DHAQ||**$K_4Fe(CN)_6$**	AO-PIM-1	r.t.	14	0.2||0.1	1	**1.5**	**0.5**	4.5		15
	PIM-EA-TB	r.t.	14	0.2||0.1	1	**1.54**	**3.2**	2		
	SPX-BP-0.95	r.t.	14	0.2||0.1	1	**1.5**	**1.2**	11		87
	SCTF-BP	r.t.	14	0.4||0.4	1	**5.2**	**~4**	< 1		18
**DHBQ**||$K_4Fe(CN)_6$	Nafion 115	-	14	1||0.4	0.5	**5.9**	**9**	~3		112
**2,3-HCNQ**||$K_4Fe(CN)_6$	Nafion 212	25	14	1||0.4	0.2	**4.8**	**3.4**	1.5		113
**Bislawsone**||$Fe(CN)_6^{3+/4+}$	Fumasep E620	-	14	2||0.3	0.78	**4.2**	**0.74**	20	√	114
**DCDHAQ**||$Fe(CN)_6^{3+/4+}$	Nafion 212	-	14	1.5||0.3	0.6	**4.9**	**0.03**	9	√	115
**ACA**||$Fe(CN)_6^{3+/4+}$	Nafion 212	-	14	1.0||0.4	0.5	**~5.6**	**2.1**	~ 4		116
FMN||**$Fe(CN)_6^{3+/4+}$**	Nafion 212	-	14	0.48||0.4	0.2	**4.83**	**0.6**	3		78
**DHPS**|$Fe(CN)_6^{3+/4+}$	Nafion 212	r.t.	14	2.8||0.31	0	**10.5**	**0.68**	14.3		77
**BHPC**||$Fe(CN)_6^{3+/4+}$	Nafion 212	-	14	1||0.8	1	**7.3**	**0.08**	23		117
**1,8-PFP**||$Fe(CN)_6^{3+/4+}$	Nafion 117	45	14	2||0.3	1.14	**4.03**	**0**	38	√	118
**4C7SFL**||$K_4Fe(CN)_6^{3+/4+}$	Nafion 212	r.t.	14	2.72||0.6	0.5	**~ 4.3**	**0.00239**	120		80
		50	14	2||0.8	1.2	**~ 8.3**	**0.138**	16		
**AQDS**||HBr	Nafion 212	40	0	2||3	-	**16**	**0.19**	<1		119,120
V^{3+}||**MB**	Fumasep FAP-375	-	-0.5	1.5||3.0	1	**11.8**	**0.76**	~2.3		121

[a]Capacity-limiting species is marked out in bold. [b]Electron concentration refers to the theoretical electron per liter of electrolyte for the catholyte (left) and anolyte (right). [c]Electron excess factor = Total electron of non-capacity limiting side/Total electron of capacity limiting side – 1. [d]Demonstrated energy density = Demonstrated discharge capacity / total volume of catholyte and anolyte × cell voltage.

Of note, RFBs exhibiting high cycling stability (with decay rates < 0.1% per day) under purely galvanostatic cycling conditions are excluded from comparisons. In these systems, the reported ultralow decay rates may not accurately reflect actual capacity loss, as the full capacity is not accessed. During purely galvanostatic cycling, capacity gain (i.e., a negative decay rate) can occur[14,95] due to fluctuations in battery polarization, such as slight increases in environment temperature. A more robust cycling protocol involves incorporating a potentiostatic hold at the end of charging and discharging steps[60]. This ensures 100% depth of discharge and provides a more accurate assessment of capacity decay in highly stable RFB systems.

Reference

64 Mizrahi Rodriguez, K. *et al.* Facile and Time-Efficient Carboxylic Acid Functionalization of PIM-1: Effect on Molecular Packing and Gas Separation Performance. *Macromolecules* **53**, 6220-6234 (2020). https://doi.org/10.1021/acs.macromol.0c00933

65 Yuan, Z. *et al.* Low-cost hydrocarbon membrane enables commercial-scale flow batteries for long-duration energy storage. *Joule* **6**, 884-905 (2022). https://doi.org/10.1016/j.joule.2022.02.016

66 Frisch, M. e. *et al.* (Gaussian, Inc., Wallingford CT, 2016).

67 Abbott, L. J., Hart, K. E. & Colina, C. M. Polymatic: a generalized simulated polymerization algorithm for amorphous polymers. *Theoretical Chemistry Accounts* **132**, 1-19 (2013). https://doi.org/10.1007/s00214-013-1334-z

68 Abbott, L. J. & Frischknecht, A. L. Nanoscale Structure and Morphology of Sulfonated Polyphenylenes via Atomistic Simulations. *Macromolecules* **50**, 1184-1192 (2017). https://doi.org/10.1021/acs.macromol.6b02232

69 Liu, M. *et al.* Barely porous organic cages for hydrogen isotope separation. *Science* **366**, 613-620 (2019). https://doi.org/10.1126/science.aax7427

70 Thompson, K. A. *et al.* N-Aryl-linked spirocyclic polymers for membrane separations of complex hydrocarbon mixtures. *Science* **369**, 310-315 (2020). https://doi.org/doi:10.1126/science.aba9806

71 McKeown, N. B. Polymers of Intrinsic Microporosity (PIMs). *Polymer* **202**, 122736 (2020). https://doi.org/https://doi.org/10.1016/j.polymer.2020.122736

72 Weber, J., Du, N. & Guiver, M. D. Influence of Intermolecular Interactions on the Observable Porosity in Intrinsically Microporous Polymers. *Macromolecules* **44**, 1763-1767 (2011). https://doi.org/10.1021/ma101447h

73 Zheng, Y. *et al.* Water Uptake Study of Anion Exchange Membranes. *Macromolecules* **51**, 3264-3278 (2018). https://doi.org/10.1021/acs.macromol.8b00034

74 Pruppacher, H. R. Self-diffusion coefficient of supercooled water. *The Journal of Chemical Physics* **56**, 101-107 (1972). https://doi.org/10.1063/1.1676831

75 Zhao, E. W. *et al.* In situ NMR metrology reveals reaction mechanisms in redox flow batteries. *Nature* **579**, 224-228 (2020). https://doi.org/10.1038/s41586-020-2081-7

76 Jing, Y. *et al.* In situ electrochemical recomposition of decomposed redox-active species in aqueous organic flow batteries. *Nature Chemistry* **14**, 1103-1109 (2022). https://doi.org/10.1038/s41557-022-00967-4

77 Hollas, A. *et al.* A biomimetic high-capacity phenazine-based anolyte for aqueous organic redox flow batteries. *Nature Energy* **3**, 508-514 (2018). https://doi.org/10.1038/s41560-018-0167-3

78 Orita, A., Verde, M. G., Sakai, M. & Meng, Y. S. A biomimetic redox flow battery based on flavin mononucleotide. *Nature communications* **7**, 13230 (2016). https://doi.org/10.1038/ncomms13230

79 Hey, D. *et al.* Identifying and preventing degradation in flavin mononucleotide-based redox flow batteries via NMR and EPR spectroscopy. *Nature Communications* **14**, 5207 (2023). https://doi.org/10.1038/s41467-023-40649-4

80 Feng, R. *et al.* Reversible ketone hydrogenation and dehydrogenation for aqueous organic redox flow batteries. *Science* **372**, 836-840 (2021). https://doi.org/10.1126/science.abd9795

81 Jin, S. *et al.* Near neutral pH redox flow battery with low permeability and long-lifetime phosphonated viologen active species. *Advanced Energy Materials* **10**, 2000100 (2020). https://doi.org/10.1002/aenm.202000100

82 DeBruler, C. *et al.* Designer two-electron storage viologen anolyte materials for neutral aqueous organic redox flow batteries. *Chem* **3**, 961-978 (2017). https://doi.org/10.1016/j.chempr.2017.11.001

83 Liu, Y. *et al.* A long-lifetime all-organic aqueous flow battery utilizing TMAP-TEMPO radical. *Chem* **5**, 1861-1870 (2019). https://doi.org/10.1016/j.chempr.2019.04.021

84 Beh, E. S. *et al.* A neutral pH aqueous organic-organometallic redox flow battery with extremely high capacity retention. *ACS Energy Letters* **2**, 639-644 (2017). https://doi.org/10.1021/acsenergylett.7b00019

85 DeBruler, C., Hu, B., Moss, J., Luo, J. & Liu, T. L. A sulfonate-functionalized viologen enabling neutral cation exchange, aqueous organic redox flow batteries toward renewable energy storage. *ACS Energy Letters* **3**, 663-668 (2018). https://doi.org/10.1021/acsenergylett.7b01302

86 Baran, M. J. *et al.* Design rules for membranes from polymers of intrinsic microporosity for crossover-free aqueous electrochemical devices. *Joule* **3**, 2968-2985 (2019). https://doi.org/10.1016/j.joule.2019.08.025

87 Zuo, P. *et al.* Sulfonated Microporous Polymer Membranes with Fast and Selective Ion Transport for Electrochemical Energy Conversion and Storage. *Angewandte Chemie International Edition* **59**, 9564-9573 (2020). https://doi.org/10.1002/anie.202000012

88 Yuan, Z. *et al.* Negatively charged nanoporous membrane for a dendrite-free alkaline zinc-based flow battery with long cycle life. *Nature communications* **9**, 3731 (2018). https://doi.org/10.1038/s41467-018-06209-x

89 Zhang, L. *et al.* Enabling graphene-oxide-based membranes for large-scale energy storage by controlling hydrophilic microstructures. *Chem* **4**, 1035-1046 (2018). https://doi.org/10.1016/j.chempr.2018.02.003

90 Yuan, Z., Duan, Y., Liu, T., Zhang, H. & Li, X. Toward a low-cost alkaline zinc-iron flow battery with a polybenzimidazole custom membrane for stationary energy storage. *IScience* **3**, 40-49 (2018). https://doi.org/10.1016/j.isci.2018.04.006

91 Chen, D., Kang, C., Duan, W., Yuan, Z. & Li, X. A non-ionic membrane with high performance for alkaline zinc-iron flow battery. *Journal of Membrane Science* **618**, 118585 (2021). https://doi.org/10.1016/j.memsci.2020.118585

92 Li, Y., Liu, Y., Xu, Z. & Yang, Z. Poly(phenylene oxide)-based ion-exchange membranes for aqueous organic redox flow battery. *Industrial & Engineering Chemistry Research* **58**, 10707-10712 (2019). https://doi.org/10.1021/acs.iecr.9b01377

93 De Porcellinis, D. *et al.* Communication-sulfonated poly (ether ether ketone) as cation exchange membrane for alkaline redox flow batteries. *Journal of The Electrochemical Society* **165**, A1137 (2018). https://doi.org/10.1149/2.1291805jes

94 Kwabi, D. G. *et al.* Alkaline Quinone Flow Battery with Long Lifetime at pH 12. *Joule* **2**, 1894-1906 (2018). https://doi.org/10.1016/j.joule.2018.07.005

95 Luo, J. *et al.* Unprecedented capacity and stability of ammonium ferrocyanide catholyte in pH neutral aqueous redox flow batteries. *Joule* **3**, 149-163 (2019). https://doi.org/10.1016/j.joule.2018.10.010

96 Amini, K. *et al.* An Extremely Stable, Highly Soluble Monosubstituted Anthraquinone for Aqueous Redox Flow Batteries. *Advanced Functional Materials* **33**, 2211338 (2023). https://doi.org/10.1002/adfm.202211338

97 Ji, Y. *et al.* A phosphonate-functionalized quinone redox flow battery at near-neutral pH with record capacity retention rate. *Advanced Energy Materials* **9**, 1900039 (2019). https://doi.org/10.1002/aenm.201900039

98 Jin, S. *et al.* A water-miscible quinone flow battery with high volumetric capacity and energy density. *ACS Energy Letters* **4**, 1342-1348 (2019). https://doi.org/10.1021/acsenergylett.9b00739

99 Wu, M. *et al.* Extremely stable anthraquinone negolytes synthesized from common precursors. *Chem* **6**, 1432-1442 (2020). https://doi.org/10.1016/j.chempr.2020.03.021

100 Jing, Y. *et al.* Anthraquinone flow battery reactants with nonhydrolyzable water-solubilizing chains introduced via a generic cross-coupling method. *ACS Energy Letters* **7**, 226-235 (2021). https://doi.org/10.1021/acsenergylett.1c02504

101 Liu, T., Wei, X., Nie, Z., Sprenkle, V. & Wang, W. A total organic aqueous redox flow battery employing a low cost and sustainable methyl viologen anolyte and 4-HO-TEMPO catholyte. *Advanced Energy Materials* **6**, 1501449 (2016). https://doi.org/10.1002/aenm.201501449

102 Janoschka, T. *et al.* An aqueous, polymer-based redox-flow battery using non-corrosive, safe, and low-cost materials. *Nature* **527**, 78-81 (2015). https://doi.org/10.1038/nature15746

103 Janoschka, T., Martin, N., Hager, M. D. & Schubert, U. S. An Aqueous Redox-Flow Battery with High Capacity and Power: The TEMPTMA/MV System. *Angewandte Chemie International Edition* **55**, 14427-14430 (2016). https://doi.org/10.1002/anie.201606472

104 Hu, B., DeBruler, C., Rhodes, Z. & Liu, T. L. Long-cycling aqueous organic redox flow battery (AORFB) toward sustainable and safe energy storage. *Journal of the American Chemical Society* **139**, 1207-1214 (2017). https://doi.org/10.1021/jacs.6b10984

105 Hu, B. *et al.* Improved radical stability of viologen anolytes in aqueous organic redox flow batteries. *Chemical communications* **54**, 6871-6874 (2018). https://doi.org/10.1039/C8CC02336K

106 Luo, J., Hu, B., Debruler, C. & Liu, T. L. A Pi-conjugation extended viologen as a two-electron storage anolyte for total organic aqueous redox flow batteries. *Angewandte Chemie* **130**, 237-241 (2018). https://doi.org/10.1002/anie.201710517

107 Huang, J. *et al.* Spatially constrained organic diquat anolyte for stable aqueous flow batteries. *ACS Energy Letters* **3**, 2533-2538 (2018). https://doi.org/10.1021/acsenergylett.8b01550

108 Tang, G. *et al.* Designing robust two-electron storage extended bipyridinium anolytes for pH-neutral aqueous organic redox flow batteries. *JACS Au* **2**, 1214-1222 (2022). https://doi.org/10.1021/jacsau.2c00184

109 Pang, S., Wang, X., Wang, P. & Ji, Y. Biomimetic amino acid functionalized phenazine flow batteries with long lifetime at near-neutral pH. *Angewandte Chemie International Edition* **60**, 5289-5298 (2021). https://doi.org/10.1002/anie.202014610

110 Gao, J. *et al.* A High Potential, Low Capacity Fade Rate Iron Complex Posolyte for Aqueous Organic Flow Batteries. *Advanced Energy Materials* **12**, 2202444 (2022). https://doi.org/10.1002/aenm.202202444

111 Li, X. *et al.* Symmetry-breaking design of an organic iron complex catholyte for a long cyclability aqueous organic redox flow battery. *Nature Energy* **6**, 873-881 (2021). https://doi.org/10.1038/s41560-021-00879-6

112 Yang, Z. *et al.* Alkaline benzoquinone aqueous flow battery for large-scale storage of electrical energy. *Advanced Energy Materials* **8**, 1702056 (2018). https://doi.org/10.1002/aenm.201702056

113 Wang, C. *et al.* High-performance alkaline organic redox flow batteries based on 2-hydroxy-3-carboxy-1, 4-naphthoquinone. *ACS Energy Letters* **3**, 2404-2409 (2018). https://doi.org/10.1021/acsenergylett.8b01296

114 Tong, L. *et al.* Molecular engineering of an alkaline naphthoquinone flow battery. *ACS Energy Letters* **4**, 1880-1887 (2019). https://doi.org/10.1021/acsenergylett.9b01321

115 Wu, M., Bahari, M., Fell, E. M., Gordon, R. G. & Aziz, M. J. High-performance anthraquinone with potentially low cost for aqueous redox flow batteries. *Journal of Materials Chemistry A* **9**, 26709-26716 (2021). https://doi.org/10.1039/D1TA08900E

116 Lin, K. *et al.* A redox-flow battery with an alloxazine-based organic electrolyte. *Nature Energy* **1**, 1-8 (2016). https://doi.org/10.1038/nenergy.2016.102

117 Wang, C. *et al.* Molecular design of fused-ring phenazine derivatives for long-cycling alkaline redox flow batteries. *ACS Energy Letters* **5**, 411-417 (2020). https://doi.org/10.1021/acsenergylett.9b02676

118 Xu, J., Pang, S., Wang, X., Wang, P. & Ji, Y. Ultrastable aqueous phenazine flow batteries with high capacity operated at elevated temperatures. *Joule* **5**, 2437-2449 (2021). https://doi.org/10.1016/j.joule.2021.06.019

119 Chen, Q., Eisenach, L. & Aziz, M. J. Cycling analysis of a quinone-bromide redox flow battery. *Journal of The Electrochemical Society* **163**, A5057 (2015). https://doi.org/10.1149/2.0081601jes

120 Huskinson, B. *et al.* A metal-free organic–inorganic aqueous flow battery. *Nature* **505**, 195-198 (2014). https://doi.org/10.1038/nature12909

121 Zhang, C. *et al.* Phenothiazine-based organic catholyte for high-capacity and long-life aqueous redox flow batteries. *Advanced Materials* **31**, 1901052 (2019). https://doi.org/10.1002/adma.201901052
